# Progress and Future Perspectives in Hydrogen Energy and Fuel Cell Technologies for Clean Energy Applications

**DOI:** 10.3390/ma19214441

**Published:** 2026-10-22

**Authors:** Muhammad Waqar, Sumyya Shah, Abid Ullah, Muhammad Humayun

**Affiliations:** 1 Department of Chemistry, University of Malakand Pakistan, Malakand 18800, Pakistan; mwaqarchemistt@gmail.com; 2 Department of Chemistry, University of Peshawar, Peshawar 25000, Pakistan; 3 Department of Advanced Energy and System Engineering, University of Science and Technology (UST), Daejeon 34113, Republic of Korea; 4 Department of Basic Sciences, University of Engineering and Technology, Peshawar 25000, Pakistan; humayunchemist@uetpeshawar.edu.pk

**Keywords:** green hydrogen, electrocatalysts, electrochemical mechanisms, hydrogen storage, renewable energy integration

## Abstract

**Highlights:**

- Comprehensive review of hydrogen production, storage, and fuel cell technologies
- Recent advances in electrocatalysts for hydrogen generation and energy conversion
- Fundamental electrochemical mechanisms governing hydrogen energy systems
- Comparative assessment of PEMFC, SOFC, PCFC, and AEMFC technologies
- Future outlook on green hydrogen, AI-driven materials discovery, and sustainable energy applications

**Abstract:**

Hydrogen energy has emerged as one of the most promising pathways for achieving sustainable, low-carbon, and net-zero energy systems in response to growing concerns regarding climate change, greenhouse gas emissions, and fossil fuel depletion. Owing to its high energy density, environmental compatibility, and versatility across multiple sectors, hydrogen is increasingly recognized as a key energy carrier for future clean energy infrastructures. In particular, hydrogen fuel cells and electrochemical conversion technologies offer highly efficient alternatives to conventional combustion-based systems while enabling significant reductions in carbon emissions across transportation, power generation, industrial manufacturing, and renewable energy integration. This review provides a comprehensive overview of recent advances in hydrogen energy and fuel cell technologies, focusing on material innovations, electrochemical mechanisms, durability, and sustainable applications. State-of-the-art hydrogen production pathways, including water electrolysis, photoelectrochemical hydrogen generation, thermochemical conversion, and biomass-assisted processes, are critically discussed together with emerging hydrogen storage and transportation technologies. Recent developments in advanced electrocatalysts, including noble-metal-free catalysts, high-entropy materials, perovskite oxides, MXene-derived catalysts, single-atom catalysts, and defect-engineered nanostructures, are evaluated in terms of catalytic activity, electronic structure modulation, stability, and scalability. The review further examines the fundamental electrochemical mechanisms governing hydrogen evolution, oxygen evolution, oxygen reduction reactions, proton transport, and oxygen-ion conduction in fuel cell systems. Various fuel cell technologies, including proton exchange membrane fuel cells, solid oxide fuel cells, proton ceramic fuel cells, and anion exchange membrane fuel cells, are comparatively analyzed with respect to operating principles, electrochemical performance, efficiency, degradation behavior, and commercial readiness. In addition, critical degradation pathways involving catalyst dissolution, carbon corrosion, electrolyte instability, and thermal degradation are discussed together with advanced durability enhancement strategies. Beyond materials and electrochemical perspectives, this review highlights sustainable applications of hydrogen technologies in fuel cell electric vehicles, heavy-duty transportation, stationary power generation, industrial decarbonization, and renewable energy storage systems. Techno-economic feasibility, lifecycle sustainability, carbon footprint analysis, infrastructure development, and global hydrogen market trends are also critically assessed. Finally, emerging future directions involving artificial intelligence-assisted materials discovery, digital hydrogen systems, integrated renewable hydrogen infrastructures, low-cost electrolyzers, and advanced hydrogen storage technologies are outlined to provide strategic insights into the future development of sustainable hydrogen economies and next-generation fuel cell technologies.

## 1. Introduction

The rapid growth of global population, industrialization, urbanization, and technological advancement has led to an unprecedented increase in worldwide energy demand over the past few decades [1]. Currently, fossil fuels, including coal, oil, and natural gas, remain the dominant energy sources, accounting for a major fraction of global energy consumption. Although fossil-fuel-based systems have supported industrial and economic development for more than a century, their extensive utilization has resulted in severe environmental and climatic consequences, including greenhouse gas (GHG) emissions, global warming, air pollution, ocean acidification, and ecosystem degradation [2,3]. Carbon dioxide (CO_2_), methane (CH_4_), and nitrous oxide (N_2_O) emissions generated from conventional energy systems are considered primary contributors to anthropogenic climate change and rising global temperatures [4]. The increasing frequency of extreme weather events, melting glaciers, rising sea levels, and environmental instability has intensified international efforts toward sustainable energy transitions and carbon neutrality targets. In response, several countries and international organizations have introduced ambitious net-zero carbon emission policies and renewable energy roadmaps to reduce dependence on fossil fuels and accelerate decarbonization of the energy sector [5,6]. However, despite the rapid deployment of renewable energy technologies such as solar, wind, hydroelectric, and geothermal systems, the intermittent nature of renewable power generation remains a major challenge for reliable and continuous energy supply. Consequently, efficient energy storage and clean energy carrier technologies are urgently required to ensure grid stability, energy security, and sustainable industrial development [7,8].

**Figure 1:**
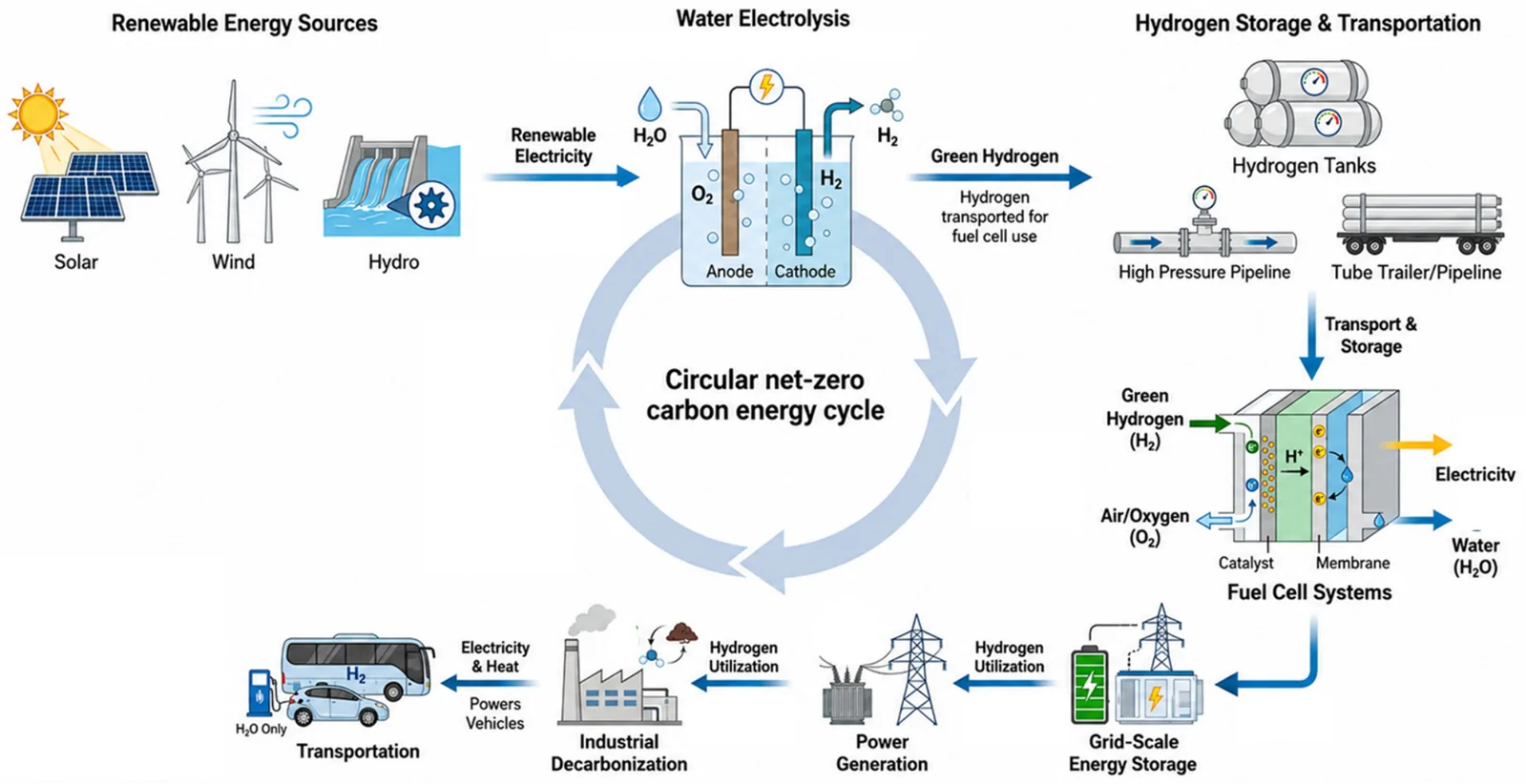
Schematic illustration of the sustainable hydrogen economy and integrated hydrogen energy cycle. The illustration was prepared with the assistance of FigLabel (AI-assisted figure design

In this context, hydrogen has emerged as one of the most promising clean energy carriers for future low-carbon and net-zero energy systems. Hydrogen possesses several attractive characteristics, including high gravimetric energy density, clean combustion products, fuel flexibility, and compatibility with renewable energy resources [7,9]. Unlike fossil fuels, hydrogen utilization in fuel cells produces electricity with water as the primary byproduct, thereby offering substantial environmental advantages [10]. As a result, hydrogen energy technologies are increasingly recognized as key components for achieving sustainable transportation, industrial decarbonization, distributed power generation, and long-duration energy storage. The concept of the hydrogen economy has gained significant global attention due to its potential to transform modern energy infrastructures into cleaner, more sustainable, and highly efficient systems [11,12]. Unlike conventional energy systems that rely heavily on fossil fuels, hydrogen offers a unique opportunity to couple the electricity, heating, mobility, and industrial sectors, thereby facilitating large-scale decarbonization and improving energy system flexibility. However, the successful realization of a hydrogen economy depends not only on sustainable hydrogen production but also on overcoming critical challenges related to production cost, infrastructure development, storage efficiency, transportation, and end-use technologies. Consequently, continued advances in hydrogen production pathways and fuel cell technologies are essential to unlock the full potential of hydrogen as a cornerstone of a carbon-neutral energy future [13,14]. Hydrogen can be produced through various pathways, including steam methane reforming, coal gasification, biomass conversion, and water electrolysis. Among these, green hydrogen produced via renewable-energy-powered water electrolysis has attracted considerable interest because it enables carbon-free hydrogen generation and supports integration with solar and wind energy systems. Advances in electrolysis technologies, including proton exchange membrane electrolyzers, alkaline electrolyzers, and solid oxide electrolysis cells, have significantly improved hydrogen production efficiency and operational flexibility [15,16]. In addition to hydrogen production, substantial progress has been achieved in hydrogen storage and transportation technologies [17,18]. To overcome the inherent challenges associated with hydrogen’s low volumetric energy density, storage safety, and transportation costs, considerable research has focused on developing advanced hydrogen storage and carrier technologies, including compressed hydrogen, cryogenic liquid hydrogen, metal hydrides, liquid organic hydrogen carriers (LOHCs), and ammonia-based systems. Each approach offers distinct advantages but also presents trade-offs in terms of storage capacity, energy efficiency, infrastructure requirements, and economic feasibility, preventing the emergence of a universally optimal solution. Consequently, the selection of an appropriate hydrogen storage strategy remains application-dependent and represents a critical barrier to large-scale hydrogen deployment. Parallel to these technological developments, substantial global investments in hydrogen infrastructure, including production facilities, refueling stations, and industrial utilization networks, have accelerated in recent years, reflecting growing confidence in hydrogen as a key pillar of future low-carbon energy systems while simultaneously highlighting the need for continued technological and economic optimization [19]. The integration of renewable energy sources, hydrogen production, storage, transportation, and fuel-cell applications within a sustainable hydrogen energy system is illustrated in Figure 1.

Hydrogen also plays a crucial role in enabling deep decarbonization of hard-to-abate sectors such as steel manufacturing, heavy-duty transportation, shipping, aviation, and chemical industries. Moreover, hydrogen-based energy systems can facilitate seasonal energy storage and grid balancing for renewable energy integration. Consequently, hydrogen technologies are now considered fundamental pillars for future sustainable energy ecosystems and circular carbon-neutral economies [20,21].

Fuel cells are electrochemical energy conversion devices that directly convert the chemical energy of hydrogen and oxygen into electricity and heat through electrochemical reactions without combustion. Compared with conventional thermal power generation technologies, fuel cells offer higher energy conversion efficiencies, lower pollutant emissions, reduced noise levels, and enhanced operational reliability. Since fuel cells avoid Carnot-cycle limitations associated with combustion engines, they can achieve superior electrical efficiencies and reduced environmental impact. A typical fuel cell consists of an anode, cathode, and electrolyte layer [22,23]. Hydrogen fuel is supplied to the anode, where hydrogen oxidation reactions generate protons and electrons. The electrons travel through an external circuit to produce electrical power, while ions are transported through the electrolyte toward the cathode [24,25]. At the cathode, oxygen reduction reactions combine oxygen, electrons, and transported ions to form water or other reaction products depending on the fuel cell type. Various fuel cell technologies have been developed based on electrolyte materials and operating temperatures, including proton exchange membrane fuel cells (PEMFCs), solid oxide fuel cells (SOFCs), proton ceramic fuel cells (PCFCs), alkaline fuel cells (AFCs), molten carbonate fuel cells (MCFCs), direct methanol fuel cells (DMFCs), and anion exchange membrane fuel cells (AEMFCs) [26,27,28,29]. Among these, PEMFCs and SOFCs have attracted substantial attention for transportation and stationary power applications, respectively, due to their high efficiencies and technological maturity. In recent years, proton ceramic fuel cells have emerged as promising next-generation systems owing to their intermediate operating temperatures, improved proton conductivity, and enhanced fuel flexibility [30]. The performance and commercialization potential of fuel cells strongly depend on the development of advanced materials, including highly active electrocatalysts, durable electrolytes, stable electrodes, and corrosion-resistant interconnects. Significant efforts have therefore been devoted to designing novel nanostructured catalysts, high-entropy materials, perovskite oxides, MXenes, and defect-engineered interfaces to improve catalytic activity, ionic conductivity, durability, and overall electrochemical performance [31].

Although numerous review articles on hydrogen energy and fuel cell technologies have been published in recent years, many reports primarily focus on either hydrogen production methods, fuel cell systems, or catalytic materials independently. A comprehensive review integrating advanced material developments, electrochemical reaction mechanisms, degradation phenomena, sustainable applications, and future commercialization perspectives remains limited. Furthermore, rapid progress in high-entropy materials, defect engineering, operando characterization techniques, artificial intelligence-assisted materials discovery, and integrated hydrogen systems necessitates an updated and critical assessment of recent advancements in the field [32]. Therefore, this review aims to provide a comprehensive and interdisciplinary overview of recent advances in hydrogen energy and fuel cell technologies with emphasis on material innovations, electrochemical mechanisms, and sustainable applications. The review systematically discusses hydrogen production, storage, and transportation technologies alongside various fuel cell systems and their operating principles [33]. Particular attention is devoted to advanced electrocatalysts, proton-conducting ceramics, electrolyte materials, interface engineering strategies, and nanostructured energy materials for enhanced electrochemical performance. In addition, the review critically analyzes fundamental reaction pathways associated with hydrogen evolution reactions (HER), oxygen evolution reactions (OER), oxygen reduction reactions (ORR), proton transport, and oxygen-ion conduction mechanisms [32]. Degradation mechanisms, durability challenges, and mitigation strategies are also comprehensively evaluated to provide insights into long-term operational stability. Furthermore, sustainable applications of hydrogen technologies in transportation, renewable energy integration, industrial decarbonization, and stationary power generation are discussed from both technological and economic perspectives. Finally, emerging trends involving artificial intelligence, machine learning-assisted catalyst discovery, smart hydrogen infrastructures, and integrated renewable hydrogen systems are highlighted to identify future research directions and commercialization opportunities. This review is expected to serve as a valuable reference for researchers, engineers, policymakers, and industrial stakeholders working toward the development of efficient, durable, and sustainable hydrogen energy systems.

### Review Methodology

This review adopts a critical narrative approach to evaluate recent advances in hydrogen energy and fuel cell technologies, with a particular focus on advanced materials for hydrogen production, storage, electrochemical energy conversion, and fuel cell systems. Relevant literature published between 2015 and 2026 was retrieved from major scientific databases, including Web of Science, Scopus, ScienceDirect, IEEE Xplore, and Google Scholar, while seminal earlier studies were included to establish the scientific foundation. Priority was given to high-impact peer-reviewed publications and authoritative reports from organizations such as the International Energy Agency (IEA) and International Renewable Energy Agency (IRENA). Rather than compiling published findings, this review critically compares material classes, electrochemical mechanisms, and technological strategies to elucidate structure–property–performance relationships, identify key scientific and technological bottlenecks, assess scalability and commercialization challenges, and highlight emerging research directions that are essential for advancing next-generation hydrogen and fuel cell technologies.

## 2. Hydrogen Energy Technologies

Hydrogen has emerged as one of the most promising energy carriers for achieving global decarbonization goals. Unlike fossil fuels, hydrogen can be utilized in fuel cells and combustion systems with water as the primary by-product, making it an attractive alternative for clean energy transitions. However, the environmental sustainability of hydrogen largely depends on the method used for its production [34,35,36]. Based on the feedstock and carbon emissions associated with production, hydrogen is generally classified into three major categories: grey hydrogen, blue hydrogen, and green hydrogen. These pathways differ significantly in terms of carbon footprint, technological maturity, production cost, and scalability.

### 2.1. Hydrogen Production Methods

Hydrogen does not naturally exist in pure form in large quantities and must therefore be extracted from compounds such as water, hydrocarbons, or biomass. Currently, the majority of global hydrogen production is derived from fossil fuels, particularly natural gas and coal. Nevertheless, increasing environmental concerns and international climate commitments have accelerated the development of low-carbon and renewable hydrogen technologies. The major hydrogen production pathways include steam methane reforming (SMR), carbon-capture-integrated processes, and renewable-energy-driven electrolysis systems.

#### 2.1.1. Grey, Blue, and Green Hydrogen

**Figure 2:**
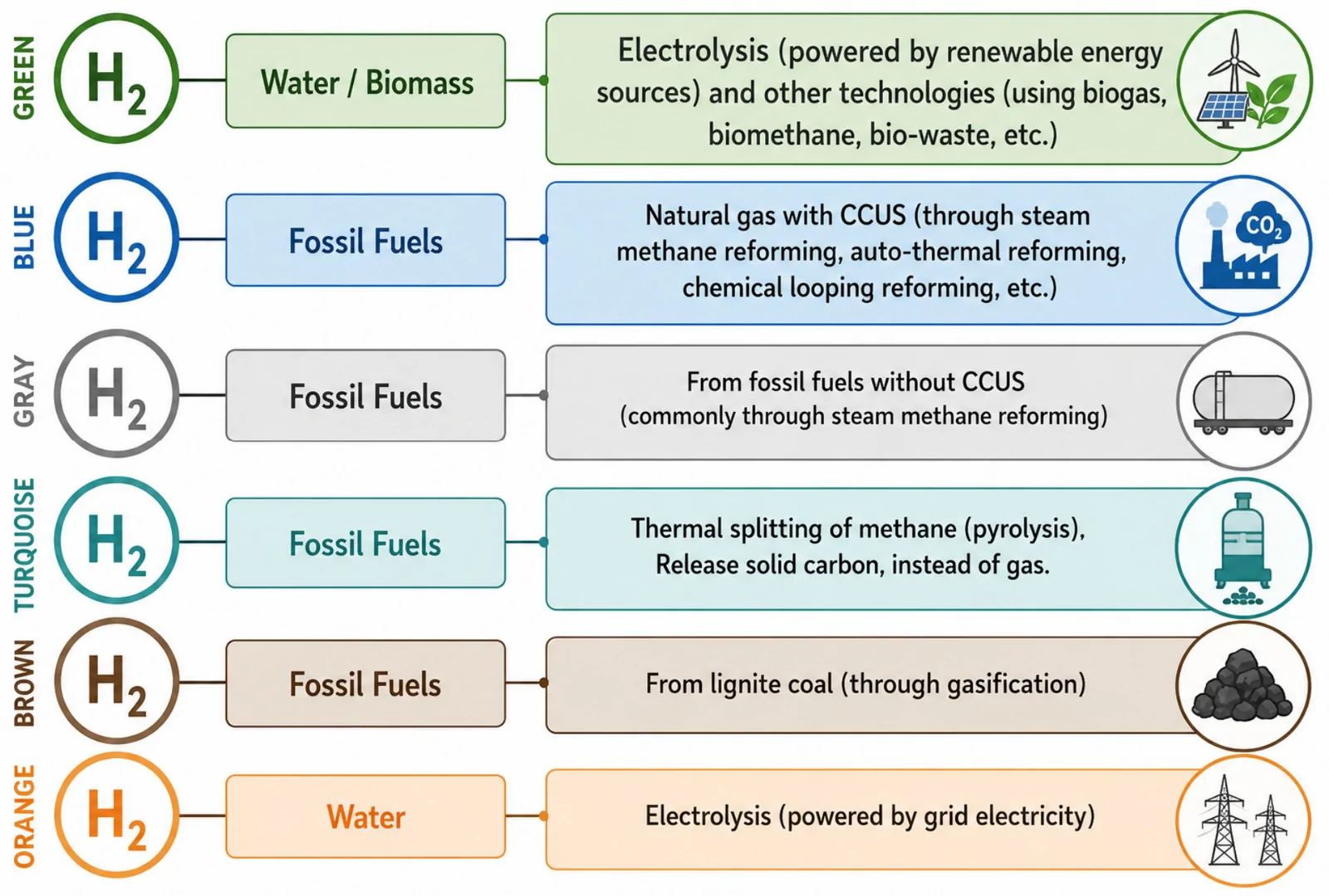
Overview of major hydrogen production pathways and their corresponding feedstocks, production technologies, carbon emissions, and commercial maturity [43].

The terminology “grey,” “blue,” and “green” hydrogen refers to the environmental impact of hydrogen production and the extent of greenhouse gas emissions generated during the process. The major hydrogen production pathways and their associated characteristics are summarized in Figure 2, highlighting the transition from carbon-intensive fossil-fuel-based hydrogen toward renewable and low-carbon hydrogen production technologies.

Grey Hydrogen

Grey hydrogen is the most widely produced form of hydrogen today and is primarily generated through steam methane reforming (SMR) of natural gas. In this process, methane reacts with high-temperature steam to produce hydrogen and carbon monoxide, followed by a water–gas shift reaction that converts carbon monoxide into carbon dioxide and additional hydrogen. The overall process is highly efficient and economically competitive; however, it releases significant amounts of carbon dioxide into the atmosphere. The primary chemical reactions involved in steam methane reforming are:

CH_4_ + H_2_O → CO + 3H_2_ (1)

followed by:

*CO* + *H*_2_*O* → *CO*_2_ + *H*_2_ (2)

Although grey hydrogen currently dominates industrial hydrogen production because of its low production cost and established infrastructure, it contributes substantially to greenhouse gas emissions. According to the International Energy Agency (IEA), hydrogen production from fossil fuels accounts for nearly 900 million tonnes of CO_2_ emissions annually, highlighting the urgent need for cleaner alternatives (IEA, 2023) [37]. Steam methane reforming remains attractive due to its technological maturity, high hydrogen yield, and compatibility with existing natural gas infrastructure. However, the environmental burden associated with carbon emissions has led researchers and policymakers to explore transitional and renewable alternatives such as blue and green hydrogen.

Blue Hydrogen

Blue hydrogen is produced using the same fossil-fuel-based processes as grey hydrogen, primarily steam methane reforming or autothermal reforming, but with the integration of carbon capture, utilization, and storage (CCUS) technologies. The objective of blue hydrogen is to reduce carbon emissions by capturing the CO_2_ generated during hydrogen production before it is released into the atmosphere [37].

Carbon capture technologies generally involve three main stages: CO_2_ separation, transportation, and long-term storage. Captured carbon dioxide can either be stored in deep geological formations or utilized in industrial applications such as enhanced oil recovery, chemical synthesis, and building materials manufacturing. Advanced CCUS systems can capture approximately 85–95% of carbon emissions from hydrogen production facilities, significantly lowering the environmental impact of fossil-fuel-derived hydrogen [38,39]. Blue hydrogen is often viewed as a transitional solution between conventional fossil-fuel-based hydrogen and fully renewable green hydrogen systems. Since it can utilize existing natural gas infrastructure, blue hydrogen offers a relatively rapid pathway for scaling hydrogen economies while renewable technologies continue to mature. However, concerns remain regarding methane leakage during natural gas extraction and transportation, as methane is a potent greenhouse gas with a significantly higher global warming potential than carbon dioxide [40]. Furthermore, the economic feasibility of blue hydrogen strongly depends on the efficiency and cost of carbon capture technologies, as well as the availability of secure geological storage sites. Despite these challenges, many countries consider blue hydrogen an important intermediate strategy for achieving short- to medium-term decarbonization goals.

Green Hydrogen

Green hydrogen is considered the cleanest and most sustainable form of hydrogen because it is produced using renewable energy sources such as solar, wind, hydroelectric, or geothermal power. The most common method for producing green hydrogen is water electrolysis, in which electricity splits water molecules into hydrogen and oxygen without generating direct carbon emissions [9]. The electrolysis reaction can be represented as:

2*H*_2_*O* → 2*H*_2_ + *O*_2_ (3)

When renewable electricity powers the electrolyzer, the resulting hydrogen production process becomes nearly carbon-neutral. Green hydrogen has gained significant attention as a critical component of future sustainable energy systems because it can decarbonize sectors that are difficult to electrify directly, including heavy industry, steel manufacturing, shipping, aviation, and long-duration energy storage. Renewable-energy-based hydrogen production offers several environmental advantages. It reduces dependence on fossil fuels, minimizes greenhouse gas emissions, and enables the integration of intermittent renewable energy sources into power grids by storing excess electricity in chemical form [41]. Moreover, hydrogen can be transported and stored for long periods, making it suitable for seasonal energy storage applications.

Despite its environmental benefits, green hydrogen currently faces economic and technological barriers. Electrolyzers remain relatively expensive, and the cost of renewable electricity significantly influences overall hydrogen production costs. Additionally, large-scale deployment requires extensive infrastructure development for hydrogen storage, transportation, and distribution. Nevertheless, continuous advancements in renewable energy technologies and declining costs of solar and wind power are expected to improve the competitiveness of green hydrogen in the coming decades. According to recent projections by the International Renewable Energy Agency (IRENA), green hydrogen could become cost-competitive with fossil-fuel-based hydrogen in regions with abundant renewable resources by 2030 [42].

#### 2.1.2. Water Electrolysis Technologies

Water electrolysis is one of the most promising technologies for sustainable hydrogen production because it enables the generation of hydrogen using electricity and water without direct carbon emissions. When powered by renewable energy sources such as solar, wind, or hydropower, electrolysis becomes a key pathway for producing green hydrogen and supporting global decarbonization strategies [38]. Electrolysis technologies are receiving increasing attention due to their potential to integrate renewable energy systems, store surplus electricity, and provide clean fuel for transportation, industrial processes, and power generation.

The fundamental principle of water electrolysis involves splitting water molecules into hydrogen and oxygen using an electric current. The overall electrochemical reaction is expressed as:

2*H*_2_*O* → 2*H*_2_ + *O*_2_ (4)

An electrolyzer typically consists of two electrodes (anode and cathode) separated by an electrolyte or membrane that facilitates ion transport. Depending on the type of electrolyte and operating conditions, water electrolysis technologies are classified into four major categories: alkaline water electrolysis (AWE), proton exchange membrane water electrolysis (PEMWE), solid oxide electrolysis cells (SOECs), and anion exchange membrane water electrolysis (AEMWE) [39,44]. The major electrolysis technologies differ substantially in operating conditions, catalyst requirements, efficiency, cost, and commercial maturity. A comparative overview is provided in Table 1.

Alkaline Water Electrolysis (AWE)

Alkaline water electrolysis is the oldest and most commercially mature electrolysis technology. It has been used for hydrogen production for more than a century and remains widely deployed in industrial applications because of its reliability, durability, and relatively low capital cost. AWE systems typically use a liquid alkaline electrolyte, such as potassium hydroxide (KOH) or sodium hydroxide (NaOH), to conduct hydroxide ions between electrodes. The electrochemical reactions in alkaline electrolysis are:

At the cathode:

2*H*_2_*O* + 2*e*^−^ → *H*_2_ + 2*OH*^−^ (5)

At the anode:

2*OH*^−^ → ½ *O*_2_ + *H*_2_*O* + 2*e*^−^ (6)

AWE systems generally operate at temperatures between 60 °C and 90 °C and offer long operational lifetimes with comparatively low maintenance requirements. Another major advantage is the use of non-precious metal catalysts such as nickel, which reduces material costs compared to other advanced electrolysis technologies. However, alkaline electrolyzers also have limitations. They typically exhibit lower current densities and slower dynamic response compared to proton exchange membrane systems, making them less suitable for fluctuating renewable energy inputs. Additionally, the liquid electrolyte can create operational challenges related to corrosion, gas crossover, and system complexity. Despite these limitations, AWE remains one of the most economically viable technologies for large-scale hydrogen production.

Proton Exchange Membrane Water Electrolysis (PEMWE)

Proton exchange membrane water electrolysis (PEMWE) is an advanced electrolysis technology that uses a solid polymer membrane as the electrolyte. The membrane conducts protons (H^+^ ions) from the anode to the cathode while preventing gas mixing. PEM electrolyzers have gained considerable attention because of their compact design, high efficiency, rapid response time, and compatibility with intermittent renewable energy sources. The electrochemical reactions in PEM electrolysis are:

At the anode:

*H*_2_*O* → ½ *O*_2_ + 2*H*^+^ + 2*e*^−^ (7)

At the cathode:

2*H*^+^ + 2*e*^−^ → *H*_2_ (8)

PEM electrolyzers generally operate at lower temperatures, typically around 50–80 °C, and can achieve high current densities and hydrogen purity. Their rapid operational flexibility makes them highly suitable for coupling with renewable electricity sources such as wind and solar energy, where power generation fluctuates continuously. Despite these advantages, PEMWE systems face several challenges. The technology relies heavily on expensive noble metal catalysts such as platinum and iridium, increasing system cost. Additionally, the proton exchange membranes require highly pure water and can experience degradation under prolonged operating conditions. Nevertheless, continuous improvements in catalyst development, membrane durability, and manufacturing processes are expected to enhance the economic viability of PEM electrolysis in the future.

Solid Oxide Electrolysis Cells (SOECs)

Solid oxide electrolysis cells (SOECs) represent a high-temperature electrolysis technology that operates typically between 700 °C and 1000 °C. Unlike low-temperature electrolyzers, SOECs utilize ceramic electrolytes capable of conducting oxygen ions at elevated temperatures. High operating temperatures improve electrochemical reaction kinetics and reduce the electrical energy required for water splitting, thereby increasing overall system efficiency.

The electrochemical reactions in SOECs are:

At the cathode:

*H*_2_*O* + 2*e*^−^ → *H*_2_ + *O*^−2^ (9)

At the anode:

*O*^−2^ → ½ *O*_2_ + 2*e*^−^ (10)

One of the most significant advantages of SOEC technology is its exceptionally high electrical efficiency, especially when integrated with industrial waste heat or concentrated solar thermal systems. SOECs can also co-electrolyze water and carbon dioxide to produce syngas, which can subsequently be converted into synthetic fuels and chemicals. However, the commercialization of SOEC technology remains limited due to several technical challenges. High operating temperatures accelerate material degradation, thermal stress, and corrosion, reducing system durability and increasing maintenance requirements. The development of stable ceramic materials and improved thermal management systems is therefore critical for the large-scale adoption of SOEC technology. Despite these challenges, SOECs are considered highly promising for future industrial-scale hydrogen and synthetic fuel production because of their superior efficiency potential.

Anion Exchange Membrane Water Electrolysis (AEMWE)

Anion exchange membrane water electrolysis (AEMWE) is an emerging technology that combines the advantages of alkaline and PEM electrolysis systems. AEM electrolyzers use a solid anion-conducting membrane to transport hydroxide ions while enabling operation in alkaline conditions. This approach allows the use of non-precious metal catalysts while maintaining a compact and simplified cell structure similar to PEM systems. The electrochemical reactions are comparable to those in alkaline electrolysis:

At the cathode:

2*H*_2_*O* + 2*e*^−^ → *H*_2_ + 2*OH*^−^ (11)

At the anode:

2*OH*^−^ → ½ *O*_2_ + *H*_2_*O* + 2*e*^−^ (12)

AEMWE technology offers several potential advantages, including reduced material costs, improved operational flexibility, and the possibility of achieving high efficiencies without relying on expensive noble metals. As a result, AEM electrolyzers are attracting increasing research interest as a cost-effective alternative for green hydrogen production. However, AEM technology is still in the early stages of development. Challenges related to membrane stability, ionic conductivity, and long-term durability continue to limit commercial deployment. Researchers are actively working to improve membrane materials and electrode architectures to enhance performance and operational lifespan. If these technical barriers are successfully addressed, AEMWE could become a highly competitive hydrogen production technology in the future. In conclusion, water electrolysis technologies are central to the development of sustainable hydrogen economies and future low-carbon energy systems. Alkaline electrolysis offers technological maturity and cost advantages, PEM electrolysis provides operational flexibility and high performance, SOECs deliver superior efficiency through high-temperature operation, and AEMWE presents an emerging low-cost alternative with significant future potential. Continued advancements in materials science, catalyst development, membrane engineering, and renewable energy integration are expected to further improve the efficiency, affordability, and scalability of electrolysis technologies worldwide.

#### 2.1.3. Emerging Hydrogen Production Routes

**Figure 3:**
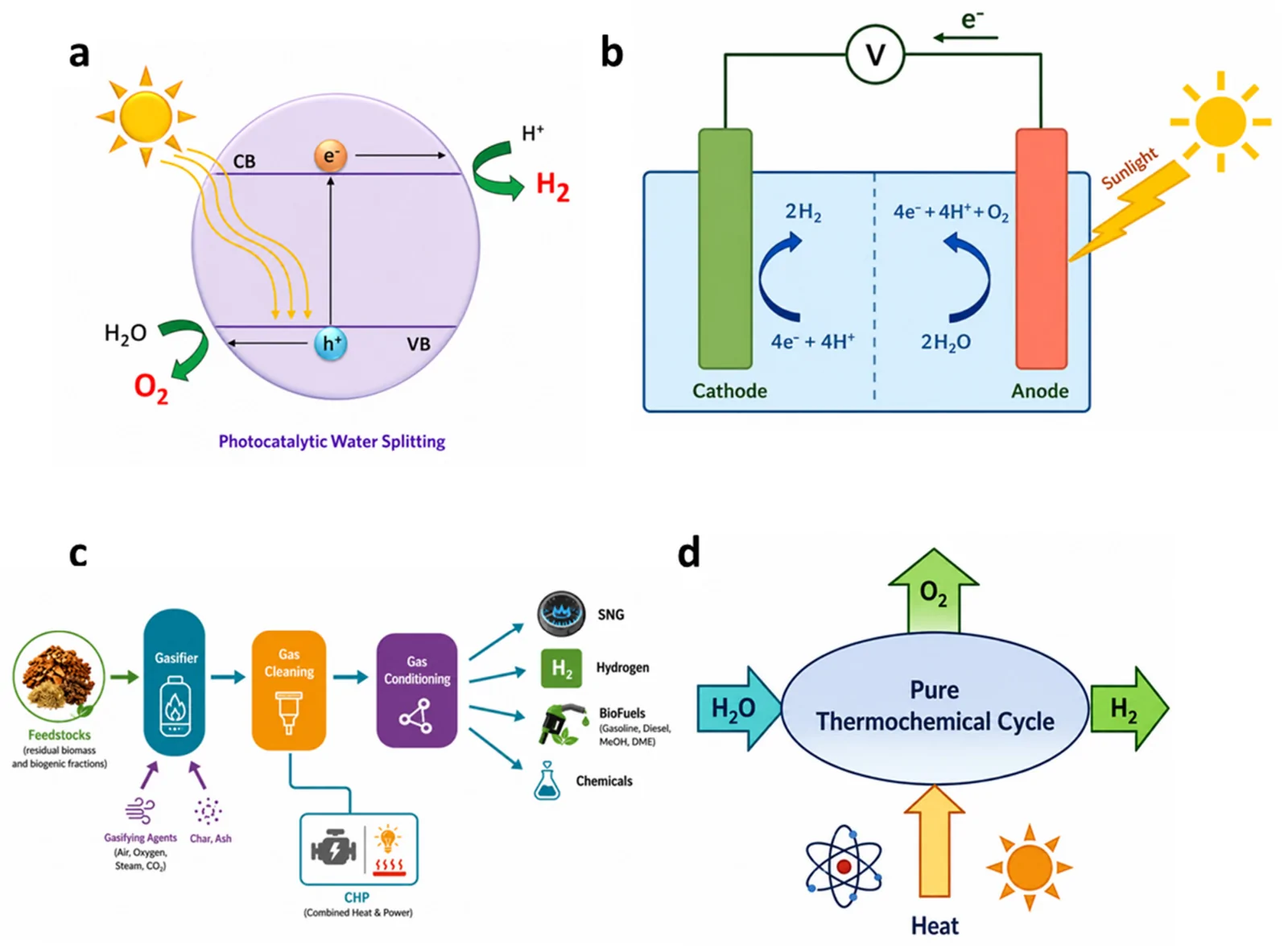
Schematic illustration of major hydrogen production pathways: (**a**) photocatalytic water splitting; (**b**) photoelectrochemical water splitting [45]; (**c**) biomass gasification and subsequent hydrogen production; (**d**) thermochemical water-splitting cycle [46]. Certain components of this figure were prepared with the assistance of OpenAI.

The growing global demand for clean and sustainable energy has accelerated research into advanced hydrogen production technologies beyond conventional fossil-fuel-based and electrolysis methods. While steam methane reforming and water electrolysis currently dominate hydrogen production, emerging hydrogen generation routes are gaining significant attention because of their potential to improve efficiency, reduce greenhouse gas emissions, and utilize abundant renewable or waste resources. These innovative approaches aim to overcome the economic and environmental limitations associated with traditional hydrogen production technologies while supporting long-term decarbonization objectives. Among the most promising emerging hydrogen production pathways are photocatalytic water splitting, photoelectrochemical hydrogen production, biomass-derived hydrogen, and thermochemical cycles. These technologies integrate solar energy, biological resources, and high-temperature chemical processes to produce hydrogen in more sustainable and environmentally friendly ways. Beyond conventional electrolysis, several emerging hydrogen production technologies are attracting significant research interest due to their potential for sustainable and low-carbon hydrogen generation. These approaches utilize diverse energy sources, including solar radiation, biomass resources, and high-temperature heat, to produce hydrogen through distinct conversion pathways. As illustrated in Figure 3, photocatalytic water splitting and photoelectrochemical hydrogen production directly harness solar energy for hydrogen generation, whereas biomass-based processes convert renewable organic feedstocks into hydrogen-rich syngas. Thermochemical cycles employ high-temperature heat from solar, nuclear, or industrial sources to drive water-splitting reactions with potentially high conversion efficiencies. Collectively, these technologies represent promising pathways toward large-scale sustainable hydrogen production.

Photocatalytic Water Splitting

Photocatalytic water splitting is an advanced solar-driven hydrogen production technology that directly converts solar energy into chemical energy using semiconductor photocatalysts. In this process, sunlight excites electrons within the photocatalyst material, generating electron–hole pairs that drive water-splitting reactions to produce hydrogen and oxygen. The overall reaction for photocatalytic water splitting is:

2*H*_2_*O* + *hν* → 2*H*_2_ + *O*_2_ (13)

where *hν* represents the absorbed photon energy from sunlight.

The fundamental mechanism involves three key steps: light absorption, charge separation, and surface catalytic reactions. When photons with sufficient energy strike the semiconductor material, electrons in the valence band become excited to the conduction band, leaving behind positively charged holes. The excited electrons participate in hydrogen evolution reactions, while the holes facilitate oxygen evolution reactions. Common photocatalyst materials include titanium dioxide (TiO_2_), zinc oxide (ZnO), cadmium sulfide (CdS), and graphitic carbon nitride (g-C_3_N_4_). Titanium dioxide is particularly popular because of its chemical stability, low cost, and resistance to corrosion. However, its large bandgap limits visible-light absorption, reducing overall solar conversion efficiency [47].

Photocatalytic water splitting offers several advantages, including direct solar-to-hydrogen conversion, low operating temperatures, and minimal environmental impact. Additionally, the process eliminates the need for external electricity, potentially reducing operational costs in the long term. Despite these benefits, the technology remains largely at the laboratory and pilot scale because of several technical challenges, including low solar conversion efficiency, rapid recombination of electron–hole pairs, limited photocatalyst stability, and insufficient hydrogen production rates. Current research focuses on improving photocatalyst materials through nanotechnology, surface modification, heterojunction engineering, and co-catalyst integration to enhance visible-light absorption and charge separation efficiency. If these challenges are successfully addressed, photocatalytic water splitting could become an economically viable pathway for large-scale green hydrogen production in the future.

**Photoelectrochemical Hydrogen Production**

Photoelectrochemical (PEC) hydrogen production combines principles of photovoltaics and electrochemistry to generate hydrogen directly from sunlight and water. Unlike photocatalytic systems, PEC systems use photoelectrodes immersed in an electrolyte to absorb solar energy and drive electrochemical water-splitting reactions.

The overall PEC water-splitting reaction is:

2*H*_2_*O* + *solar energy* → 2*H*_2_ + *O*_2_ (14)

In a typical PEC system, the semiconductor photoanode absorbs sunlight, generating electron–hole pairs. The holes oxidize water molecules to produce oxygen, while electrons travel through an external circuit to the cathode, where hydrogen evolution occurs. PEC systems can operate with either a photoanode, photocathode, or tandem cell configuration to maximize solar energy utilization. One of the major advantages of PEC hydrogen production is its potential for highly efficient direct solar-to-hydrogen conversion without requiring separate photovoltaic and electrolysis units. This integrated design can reduce system complexity and potentially lower overall production costs. PEC systems also offer opportunities for decentralized hydrogen generation in remote or off-grid areas with abundant solar resources.

However, several technical barriers continue to hinder commercialization. Semiconductor materials used in PEC cells often suffer from photocorrosion, low stability, and insufficient visible-light absorption [48]. Furthermore, achieving efficient charge transport and minimizing energy losses remain major research challenges. Materials such as silicon, hematite (Fe_2_O_3_), tungsten oxide (WO_3_), and gallium indium phosphide are currently being investigated to improve PEC performance. Recent advancements in nanostructured photoelectrodes, tandem PEC devices, and protective catalyst coatings have significantly improved conversion efficiencies and operational stability. Nevertheless, large-scale implementation still requires further progress in material durability, cost reduction, and system integration. PEC hydrogen production is therefore considered one of the most innovative and potentially transformative approaches for future renewable hydrogen generation.

**Biomass-Derived Hydrogen**

Biomass-derived hydrogen production utilizes organic materials such as agricultural residues, forestry waste, municipal solid waste, algae, and industrial biowaste as feedstocks for hydrogen generation. Since biomass absorbs atmospheric carbon dioxide during growth, biomass-based hydrogen production is often regarded as carbon-neutral or low-carbon, depending on the conversion technology and lifecycle emissions. Hydrogen can be produced from biomass through several thermochemical and biological pathways, including gasification, pyrolysis, fermentation, and anaerobic digestion. Among these, biomass gasification is one of the most widely studied methods. In gasification, biomass reacts with steam, oxygen, or air at high temperatures to produce synthesis gas (syngas), which primarily consists of hydrogen, carbon monoxide, methane, and carbon dioxide.

A simplified gasification reaction can be represented as:

*Biomass* + *H*_2_*O* + *heat* → *H*_2_ + *CO* + *CO*_2_ + *CH*_4_ (15)

The produced syngas can subsequently undergo water–gas shift reactions and purification processes to increase hydrogen concentration. Biological hydrogen production methods, such as dark fermentation and photo-fermentation, use microorganisms to convert organic matter into hydrogen under anaerobic conditions. These processes operate under mild temperatures and pressures, making them environmentally attractive alternatives. However, biological hydrogen production generally exhibits lower yields and slower reaction rates compared to thermochemical methods. Biomass-derived hydrogen offers several advantages, including waste utilization, reduced landfill burden, renewable feedstock availability, and potential negative carbon emissions when combined with carbon capture technologies. Nevertheless, challenges such as feedstock variability, transportation logistics, tar formation during gasification, and relatively low conversion efficiencies continue to limit widespread commercialization. Ongoing research focuses on improving catalyst performance, optimizing reactor designs, integrating carbon capture systems, and enhancing microbial activity for biological hydrogen production. With proper technological development and sustainable biomass management, biomass-derived hydrogen could become an important contributor to future renewable energy systems.

**Thermochemical Cycles**

Thermochemical hydrogen production involves a series of chemical reactions driven by high-temperature heat sources to split water into hydrogen and oxygen without direct electricity input. These processes typically utilize solar thermal energy, nuclear heat, or industrial waste heat to achieve the extremely high temperatures required for the reactions.

Among the most studied thermochemical cycles are the sulfur–iodine (S–I) cycle, copper–chlorine (Cu–Cl) cycle, and metal oxide redox cycles. The sulfur–iodine cycle is particularly notable because of its high theoretical efficiency and ability to operate without fossil fuels. A simplified overall thermochemical reaction is:

*H*_2_*O* + *heat* → *H*_2_ + ½ *O*_2_ (16)

Thermochemical cycles divide the water-splitting process into multiple intermediate chemical reactions, allowing hydrogen production at lower energy losses than direct thermal decomposition of water. One of the primary advantages of thermochemical hydrogen production is its potential for very high energy efficiency, especially when integrated with concentrated solar power plants or next-generation nuclear reactors. Additionally, these systems can produce large quantities of hydrogen continuously, making them attractive for industrial-scale applications. However, thermochemical cycles face substantial engineering and material challenges. High operating temperatures often exceed 800 °C, requiring advanced corrosion-resistant materials and sophisticated heat management systems. The complexity of multi-step chemical processes, system integration difficulties, and high capital costs also remain major barriers to commercialization. Despite these challenges, thermochemical hydrogen production continues to receive significant research interest because of its potential to achieve large-scale, carbon-free hydrogen generation using abundant thermal energy resources. Advances in materials science, reactor engineering, and solar thermal technologies are expected to improve the practicality and efficiency of thermochemical cycles in the future. In summary, emerging hydrogen production routes represent critical innovations for the future development of sustainable energy systems. Photocatalytic and photoelectrochemical technologies harness solar energy directly for hydrogen generation, biomass-derived hydrogen utilizes renewable organic resources and waste materials, and thermochemical cycles exploit high-temperature heat sources for efficient water splitting. Although these technologies remain at various stages of research and development, continued advancements in materials engineering, catalyst design, renewable energy integration, and process optimization are expected to significantly enhance their commercial viability and contribution to global clean hydrogen economies.

### 2.2. Hydrogen Storage Technologies

Hydrogen storage is a critical component of the hydrogen energy system because it directly influences the efficiency, safety, transportation, and practical implementation of hydrogen as a clean energy carrier. Although hydrogen possesses one of the highest gravimetric energy densities among fuels, its extremely low volumetric density under ambient conditions creates significant storage challenges. Efficient storage technologies are therefore essential for enabling large-scale hydrogen utilization in transportation, stationary power generation, industrial applications, and renewable energy integration. Hydrogen can be stored using physical, material-based, and chemical storage methods. Among these, physical storage technologies are currently the most commercially developed and widely implemented due to their relative technological maturity and compatibility with existing infrastructure. Physical hydrogen storage primarily includes compressed gaseous hydrogen and cryogenic liquid hydrogen systems. These methods aim to increase hydrogen density either through high-pressure compression or ultra-low-temperature liquefaction. Hydrogen storage technologies exhibit substantial differences in storage density, operating conditions, safety, and scalability. A comparative assessment of major hydrogen storage approaches is summarized in Table 2.

#### 2.2.1. Physical Storage

Physical storage technologies store hydrogen either as a compressed gas or as a cryogenic liquid. These approaches rely on modifying the thermodynamic state of hydrogen to improve volumetric energy density while maintaining safe and efficient storage conditions.

Compressed Hydrogen

Compressed hydrogen storage is the most widely used and technologically mature method for hydrogen storage and transportation. In this approach, hydrogen gas is stored at very high pressures, typically ranging from 350 bar to 700 bar, within specially designed pressure vessels. Compression significantly increases the volumetric density of hydrogen, making it suitable for mobile and stationary applications such as fuel cell electric vehicles (FCEVs), hydrogen refueling stations, and industrial hydrogen supply systems. The behavior of compressed hydrogen can be understood using the ideal gas relationship:

*PV* = *nRT* (17)

where P represents pressure, V is volume, n is the number of moles, R is the universal gas constant, and T is temperature. According to this relationship, increasing pressure reduces the storage volume required for hydrogen gas.

Compressed hydrogen storage systems are generally categorized into four major tank types based on material composition and structural design:

Type I: Fully metallic cylinders, usually made of steel or aluminum.

Type II: Metal cylinders reinforced with composite materials.

Type III: Aluminum liners fully wrapped with carbon-fiber composites.

Type IV: Polymer liners fully reinforced with carbon-fiber composites.

Modern hydrogen vehicles primarily utilize Type III and Type IV tanks because of their lightweight structure, high strength, and improved storage capacity. Carbon-fiber-reinforced composite tanks are particularly advantageous because they can withstand extremely high pressures while minimizing vehicle weight. Compressed hydrogen storage offers several important advantages. First, the technology is relatively simple and commercially available. Second, gaseous hydrogen can be rapidly filled and discharged, making it suitable for transportation applications requiring fast refueling. Third, compressed hydrogen systems can operate at ambient temperatures, eliminating the need for complex cryogenic cooling systems. Despite these advantages, compressed hydrogen storage also faces several technical and economic limitations. Hydrogen compression requires substantial energy input, which reduces overall system efficiency. Additionally, high-pressure tanks are expensive because of the advanced composite materials required for safety and durability. Another major challenge involves hydrogen embrittlement, a phenomenon in which hydrogen atoms diffuse into metals and weaken structural integrity over time. Safety considerations are also critical because hydrogen is highly flammable and stored under extremely high pressures. Consequently, storage vessels must comply with rigorous engineering standards, leak detection systems, and safety protocols to minimize explosion risks. Although compressed hydrogen storage currently dominates hydrogen mobility applications, researchers continue to develop advanced lightweight materials, improved tank architectures, and higher-pressure systems to enhance storage efficiency and reduce costs.

Cryogenic Liquid Hydrogen

Cryogenic liquid hydrogen storage involves cooling hydrogen gas to extremely low temperatures until it liquefies. Hydrogen becomes liquid at approximately −253 °C (20 K) under atmospheric pressure. Liquefaction significantly increases hydrogen density, enabling much larger quantities of hydrogen to be stored within smaller volumes compared to compressed gas storage. The liquefaction process can be represented conceptually as:

H_2(g)_ → H_2(l)_

Liquid hydrogen possesses a volumetric energy density nearly four times greater than compressed gaseous hydrogen at 700 bar, making it particularly attractive for applications requiring high energy storage capacity, such as aerospace systems, maritime transport, heavy-duty transportation, and long-distance hydrogen distribution. Cryogenic hydrogen is stored in highly insulated double-walled tanks equipped with vacuum layers and reflective materials to minimize heat transfer from the surroundings. These tanks are specifically designed to maintain ultra-low temperatures and reduce hydrogen evaporation losses. One of the major advantages of liquid hydrogen storage is its superior energy density, which allows larger amounts of hydrogen to be transported and stored more efficiently. Liquid hydrogen has therefore been extensively used in aerospace applications, particularly as rocket fuel in space exploration programs because of its high specific energy and lightweight properties.

However, cryogenic hydrogen storage also presents significant technical and economic challenges. The liquefaction process itself is highly energy-intensive, consuming approximately 30–40% of the energy content of the stored hydrogen. This reduces overall energy efficiency and increases operational costs. Another major issue is hydrogen boil-off, which occurs when heat gradually enters the storage tank and causes a portion of the liquid hydrogen to evaporate. Continuous evaporation leads to pressure buildup and hydrogen losses during long-term storage. Managing boil-off gas requires sophisticated insulation systems, pressure control mechanisms, and venting technologies. Additionally, the extremely low operating temperatures create material compatibility challenges. Many conventional materials become brittle under cryogenic conditions, necessitating the use of specialized alloys and insulation materials capable of maintaining structural integrity at ultra-low temperatures. Safety is also a crucial consideration in cryogenic storage systems. Liquid hydrogen can rapidly expand upon vaporization, and accidental leaks may create highly flammable hydrogen–air mixtures. Therefore, advanced monitoring systems, thermal insulation technologies, and strict safety standards are essential for safe operation.

Despite these challenges, cryogenic liquid hydrogen storage remains highly promising for future large-scale hydrogen transportation and high-energy-demand applications. Ongoing research focuses on improving liquefaction efficiency, minimizing boil-off losses, developing advanced insulation materials, and integrating cryogenic storage with renewable hydrogen production systems. In summary, physical hydrogen storage technologies play a fundamental role in enabling hydrogen-based energy systems. Compressed hydrogen storage provides a technologically mature and practical solution for transportation and distributed applications, while cryogenic liquid hydrogen offers superior energy density for large-scale and long-distance energy storage needs. Although both technologies face challenges related to energy efficiency, material durability, cost, and safety, continuous advancements in storage materials, tank engineering, thermal insulation, and system integration are expected to significantly enhance the feasibility and commercialization of hydrogen storage infrastructure in the future.

#### 2.2.2. Material-Based Storage

Material-based hydrogen storage technologies have emerged as highly promising alternatives to conventional compressed and cryogenic hydrogen storage systems. Unlike physical storage methods that rely on high pressure or extremely low temperatures, material-based storage involves storing hydrogen within the internal structure or chemical composition of solid or liquid materials. These technologies offer significant advantages in terms of volumetric hydrogen density, operational safety, and potential energy efficiency, making them attractive for future hydrogen transportation, stationary energy systems, and portable applications. The fundamental principle of material-based hydrogen storage is the reversible interaction between hydrogen and storage materials through physical adsorption, chemical bonding, or absorption mechanisms. Depending on the nature of hydrogen interaction, material-based storage systems are broadly categorized into metal hydrides, chemical hydrides, and porous materials such as metal–organic frameworks (MOFs) and carbon-based nanostructures. Although these technologies are still under active research and development, they are considered crucial for overcoming the storage limitations associated with conventional hydrogen systems.

Metal Hydrides

Metal hydrides are among the most extensively studied solid-state hydrogen storage materials. In these systems, hydrogen is stored through reversible chemical reactions between hydrogen molecules and metals or metal alloys. When exposed to hydrogen gas under appropriate pressure and temperature conditions, the metal lattice absorbs hydrogen atoms to form metal hydrides. Hydrogen can later be released through controlled heating or pressure reduction. A general metal hydride formation reaction is represented as:

(18) $$M+\frac{x}{2}H_{2}⇌\mathrm{MHx}$$

where M represents the metal or alloy and MH_x_ denotes the formed metal hydride.

Metal hydrides offer several important advantages over conventional storage technologies. One of their primary benefits is high volumetric hydrogen density, which can exceed that of compressed gaseous hydrogen. Additionally, hydrogen is stored at relatively low pressures, significantly improving operational safety and reducing explosion risks. Since hydrogen is chemically bonded within the material structure, leakage and rapid combustion hazards are minimized. Several classes of metal hydrides have been investigated for hydrogen storage applications, including: Intermetallic hydrides (LaNi_5_H_6_, TiFeH_2_), Light metal hydrides (MgH_2_, AlH_3_), Complex hydrides (NaAlH_4_, LiBH_4_), Transition metal hydrides. Among these, magnesium hydride (MgH_2_) is particularly attractive because of its high hydrogen storage capacity and low cost. However, it requires relatively high temperatures for hydrogen desorption, which limits practical application. The hydrogen absorption and desorption process in metal hydrides is strongly influenced by thermodynamic and kinetic factors. Efficient hydrogen release often requires elevated temperatures because the metal–hydrogen bond strength can be substantial. Researchers are therefore developing catalysts, nanostructured materials, and alloy modifications to improve hydrogen sorption kinetics and reduce operating temperatures.

Despite their advantages, metal hydrides also face several limitations. Many hydride materials exhibit slow hydrogen charging and discharging rates, high material weight, and degradation after repeated hydrogen cycling. Additionally, some hydrides require complex thermal management systems to control heat generated during hydrogen absorption and release reactions. Nevertheless, metal hydrides remain highly promising for stationary hydrogen storage systems, portable power devices, and niche transportation applications where safety and compact storage are critical considerations.

Chemical Hydrides

Chemical hydrides store hydrogen through stable chemical compounds that release hydrogen via chemical reactions such as hydrolysis, thermolysis, or catalytic decomposition. Unlike metal hydrides, many chemical hydride systems are not fully reversible under practical operating conditions, meaning that the spent material often requires regeneration in external industrial facilities. Chemical hydrides generally possess exceptionally high gravimetric hydrogen storage capacities, making them attractive for portable and mobile energy applications. Common chemical hydride materials include sodium borohydride (NaBH_4_), ammonia borane (NH_3_BH_3_), lithium hydride (LiH), and hydrazine-based compounds. One widely studied hydrogen release reaction involves sodium borohydride hydrolysis:

*NaBH*_4_ + 2*H*_2_*O* → *NaBO*_2_ + 4*H*_2_ (19)

Similarly, ammonia borane decomposition can release significant quantities of hydrogen:

*NH*_3_*BH*_3_ → *BN* + 3*H*_2_ (20)

Chemical hydrides offer several important advantages. First, they provide very high hydrogen storage capacities by weight, often exceeding those of compressed gas systems. Second, hydrogen release can occur at relatively low temperatures and moderate pressures. Third, many chemical hydrides exist as stable solids or liquids, simplifying storage and transportation logistics. These materials are particularly attractive for portable electronics, military applications, backup power systems, and small-scale fuel cell devices where compact energy storage is essential. However, chemical hydride systems also face significant technical and economic challenges. One of the major limitations is the irreversibility of many hydrogen release reactions, which necessitates external regeneration processes that are often energy-intensive and expensive. Additionally, by-products generated during decomposition reactions may require careful handling and disposal. Another challenge involves controlling hydrogen release rates and ensuring stable reaction kinetics. In some cases, catalysts are required to improve hydrogen generation efficiency and reduce unwanted side reactions. Furthermore, the long-term commercial viability of chemical hydrides depends heavily on reducing material costs and improving regeneration technologies. Despite these challenges, chemical hydrides continue to attract strong research interest because of their exceptional hydrogen storage density and potential for compact energy storage systems.

Porous Materials (MOFs and Carbon Materials)

Porous materials represent a rapidly growing category of advanced hydrogen storage technologies that rely on physical adsorption of hydrogen molecules onto materials with extremely high surface areas. These materials contain nanoscale pores capable of trapping large amounts of hydrogen through weak intermolecular interactions known as physisorption. Two of the most extensively studied porous storage materials are metal–organic frameworks (MOFs) and carbon-based materials.

Metal-Organic Frameworks (MOFs)

MOFs are crystalline porous materials composed of metal ions or clusters connected by organic ligands to form highly ordered three-dimensional structures. One of the defining characteristics of MOFs is their exceptionally high internal surface area, which can exceed several thousand square meters per gram. Hydrogen storage in MOFs occurs primarily through adsorption within the porous structure:

*H*_2(g)_ → *H*_2(adsorbed)_ (21)

The tunable pore size, large surface area, and adjustable chemical functionality of MOFs make them highly attractive for hydrogen storage applications. Researchers can tailor MOF structures to optimize hydrogen adsorption capacity, adsorption energy, and gas selectivity. MOFs offer several advantages, including lightweight structure, high porosity, and potential for reversible hydrogen storage. Additionally, hydrogen adsorption and desorption occur rapidly, enabling efficient charging and discharge cycles. However, practical hydrogen storage capacities at room temperature remain relatively low because physisorption interactions are weak. Many MOF systems therefore require cryogenic temperatures to achieve high hydrogen uptake. Improving room-temperature hydrogen storage performance remains a major research challenge.

Carbon-Based Materials

Carbon-based materials such as activated carbon, carbon nanotubes (CNTs), graphene, and fullerene structures have also been extensively investigated for hydrogen storage. These materials possess high surface areas, low density, excellent thermal conductivity, and good chemical stability. Hydrogen adsorption in carbon materials similarly occurs through physisorption mechanisms on the material surface and within nanopores. Carbon nanotubes and graphene-based structures are particularly attractive because of their unique nanostructures and exceptional mechanical properties. Carbon materials offer several advantages, including lightweight design, fast adsorption kinetics, low toxicity, and relatively low production costs. Additionally, nanostructured carbon materials can potentially be modified chemically or structurally to enhance hydrogen adsorption capacity.

Despite these benefits, hydrogen storage capacities under ambient conditions are still insufficient for many commercial applications. Researchers are therefore exploring advanced nanostructuring techniques, metal doping, surface functionalization, and hybrid composite materials to improve adsorption performance. Overall, porous materials represent one of the most innovative areas of hydrogen storage research because they offer the possibility of safe, lightweight, and reversible hydrogen storage systems without requiring extremely high pressures or severe cryogenic conditions. In conclusion, material-based hydrogen storage technologies provide promising alternatives to conventional physical storage methods by enabling safer, denser, and potentially more energy-efficient hydrogen storage solutions. Metal hydrides offer high volumetric density and operational safety, chemical hydrides provide exceptional gravimetric storage capacity, and porous materials such as MOFs and carbon nanostructures present innovative pathways for reversible hydrogen adsorption. Although challenges related to cost, operating conditions, reversibility, and storage efficiency remain, ongoing advancements in nanotechnology, materials engineering, and catalyst development are expected to significantly accelerate the commercialization and practical deployment of these advanced hydrogen storage systems in future hydrogen economies.

### 2.3. Hydrogen Transportation and Infrastructure

The successful development of a hydrogen-based energy economy depends not only on efficient hydrogen production and storage technologies but also on the establishment of reliable transportation networks and supporting infrastructure. Hydrogen transportation and infrastructure systems are essential for connecting production facilities with end users in industrial, transportation, residential, and power-generation sectors. Since hydrogen possesses unique physical and chemical properties, including low volumetric density, high diffusivity, and flammability, specialized infrastructure and strict safety measures are required for its large-scale distribution and utilization. Hydrogen infrastructure encompasses pipelines, storage terminals, liquefaction plants, transport vehicles, refueling stations, monitoring systems, and regulatory frameworks. The expansion of hydrogen infrastructure is considered one of the most significant challenges for the commercialization of hydrogen energy systems because it requires substantial investments, technological adaptation, and coordinated policy support.

Pipelines and Transport Systems

Hydrogen can be transported in gaseous or liquid form using pipelines, road tankers, rail systems, and maritime shipping technologies. The choice of transportation method depends on factors such as transportation distance, hydrogen quantity, infrastructure availability, energy efficiency, and economic feasibility.

Hydrogen Pipelines

Pipelines are considered one of the most efficient and cost-effective methods for transporting large quantities of hydrogen over long distances. Hydrogen pipelines operate similarly to natural gas pipelines but require specialized materials and engineering solutions because hydrogen molecules are significantly smaller and more diffusive than methane molecules.

Hydrogen transportation through pipelines generally involves compressed gaseous hydrogen flowing under high pressure. The flow characteristics of hydrogen can be described using fluid transport principles, where pressure gradients drive gas movement through the pipeline system. Hydrogen pipeline infrastructure can be categorized into:

- Dedicated hydrogen pipelines
- Blended hydrogen–natural gas pipelines
- Retrofitted natural gas pipelines

Dedicated hydrogen pipelines are specifically designed for pure hydrogen transport and are already used extensively in industrial regions for supplying refineries and chemical plants. Several countries, particularly in Europe and North America, are planning large-scale hydrogen pipeline networks to support future clean energy transitions. One of the major advantages of hydrogen pipelines is their ability to continuously transport large hydrogen volumes with relatively low operating costs once infrastructure is established. Pipelines also provide higher energy efficiency compared to road transportation methods for long-distance distribution However, hydrogen pipelines face several technical challenges. One of the most critical issues is hydrogen embrittlement, a process in which hydrogen atoms penetrate metallic pipeline materials, causing cracks, reduced ductility, and structural weakening over time. Pipeline materials must therefore be carefully selected to withstand prolonged hydrogen exposure. Hydrogen leakage is another important concern because hydrogen molecules are extremely small and can diffuse through seals and joints more easily than natural gas. Furthermore, hydrogen has a wide flammability range and low ignition energy, increasing the importance of leak detection and ventilation systems.

Researchers and industries are currently investigating the feasibility of blending hydrogen with natural gas in existing pipeline systems as an intermediate solution for reducing carbon emissions without requiring entirely new infrastructure. Hydrogen blending percentages typically range from 5% to 20%, depending on pipeline materials and end-use compatibility. In addition to pipelines, hydrogen can also be transported using high-pressure tube trailers, cryogenic liquid hydrogen tankers, rail transport systems, and ships. Compressed gaseous hydrogen is commonly transported by tube trailers for short to medium distances, whereas liquid hydrogen transport is preferred for large-scale distribution because of its higher volumetric density. Emerging transportation approaches include hydrogen carriers such as ammonia (NH_3_) and liquid organic hydrogen carriers (LOHCs), which enable easier long-distance transportation and storage. These carriers chemically bind hydrogen and release it at the destination through catalytic processes. The development of efficient hydrogen transportation systems is therefore critical for establishing integrated hydrogen supply chains and enabling large-scale commercialization of hydrogen technologies worldwide.

Hydrogen Refueling Stations

Hydrogen refueling stations (HRS) are essential components of hydrogen transportation infrastructure, particularly for fuel cell electric vehicles (FCEVs). These stations are designed to safely store, compress, dispense, and monitor hydrogen fuel supplied to vehicles and other mobile applications. A typical hydrogen refueling station consists of several major subsystems:

- Hydrogen supply or production unit
- Compression systems
- High-pressure storage tanks
- Cooling systems
- Dispensing units
- Monitoring and safety control systems

Hydrogen supplied to refueling stations may originate from centralized production facilities or be generated onsite through electrolysis or steam methane reforming. Onsite electrolysis using renewable electricity is increasingly considered an attractive solution for decentralized green hydrogen production. Hydrogen dispensing generally occurs at standardized pressures of 350 bar or 700 bar depending on vehicle type and storage tank design. Heavy-duty vehicles such as buses and trucks often use 350-bar systems, whereas passenger fuel cell vehicles commonly operate at 700 bar to maximize driving range. One of the major advantages of hydrogen refueling technology is the rapid fueling time. Fuel cell vehicles can typically be refueled within 3–5 min, making hydrogen transportation highly competitive with conventional gasoline vehicles in terms of convenience and operational flexibility. However, hydrogen refueling infrastructure remains limited in many regions because of high capital costs, low hydrogen demand in early market stages, and the need for standardized fueling protocols. The establishment of widespread refueling networks requires significant investments in compressors, storage systems, safety equipment, and distribution logistics.

Safety considerations are particularly important in hydrogen refueling stations due to the high pressures involved and hydrogen’s flammability characteristics. Advanced leak detection systems, emergency shutdown systems, ventilation mechanisms, and explosion-resistant equipment are therefore integrated into station designs. International standards. Governments and private industries are actively investing in hydrogen refueling networks as part of broader decarbonization and clean transportation initiatives.

The expansion of hydrogen refueling infrastructure is expected to play a crucial role in accelerating the adoption of fuel cell vehicles and supporting the commercialization of hydrogen mobility systems globally.

Safety Considerations and Regulations

Safety is one of the most critical aspects of hydrogen transportation and infrastructure development. Although hydrogen offers substantial environmental benefits, its physical properties create unique safety challenges that require specialized engineering designs, operational procedures, and regulatory oversight. Hydrogen is highly flammable and possesses a wide flammability range in air, approximately 4–75% by volume. It also has very low ignition energy, meaning that even small sparks or static electricity can ignite hydrogen–air mixtures. Furthermore, hydrogen flames are nearly invisible under daylight conditions, complicating fire detection. Because hydrogen molecules are extremely small and light, they diffuse rapidly and can leak through tiny openings or imperfections in storage and transportation systems. Rapid dispersion can reduce localized explosion risks in open environments; however, hydrogen accumulation in confined spaces may create severe explosion hazards. Several key safety considerations in hydrogen systems include:

- Leak detection and monitoring
- Ventilation and dispersion management
- Pressure relief systems
- Material compatibility and embrittlement prevention
- Fire suppression systems
- Emergency shutdown protocols
- Explosion-resistant equipment

Hydrogen leak detection technologies often utilize electrochemical sensors, catalytic sensors, thermal conductivity sensors, or optical detection systems. These monitoring systems are integrated into pipelines, storage facilities, refueling stations, and industrial hydrogen plants to provide real-time safety monitoring. Material compatibility is another major concern because prolonged hydrogen exposure can weaken metals through hydrogen embrittlement. Engineers must therefore use specialized alloys, composites, and protective coatings to ensure structural reliability. Cryogenic hydrogen systems additionally require protection against extreme cold temperatures, pressure buildup from hydrogen boil-off, and thermal stresses. Proper insulation and venting systems are essential for maintaining safe operation in liquid hydrogen infrastructure. To ensure public safety and industrial reliability, numerous international organizations and regulatory agencies have established hydrogen safety standards and guidelines. Important standards and regulatory frameworks include:

- ISO (International Organization for Standardization) standards
- IEC (International Electrotechnical Commission) safety regulations
- NFPA 2 Hydrogen Technologies Code
- SAE International hydrogen fueling standards
- European Hydrogen Safety Panel guidelines

These regulations define technical requirements for hydrogen production plants, transportation systems, storage facilities, fueling stations, and end-use applications. Compliance with these standards is critical for minimizing operational risks and promoting public confidence in hydrogen technologies.

Governments worldwide are increasingly developing hydrogen roadmaps, infrastructure strategies, and safety frameworks to support the transition toward hydrogen economies. Public acceptance and large-scale commercialization of hydrogen technologies will strongly depend on maintaining high safety standards, robust regulatory oversight, and continuous technological improvements. In summary, hydrogen transportation and infrastructure systems are fundamental to the practical implementation of hydrogen energy technologies. Pipelines and advanced transport systems enable efficient hydrogen distribution, refueling stations support hydrogen mobility applications, and comprehensive safety regulations ensure secure operation across the hydrogen value chain. Although substantial technical, economic, and regulatory challenges remain, ongoing investments in infrastructure development, safety engineering, and international standardization are expected to significantly accelerate the growth of global hydrogen energy systems in the coming decades.

## 3. Fundamentals of Fuel Cell Technologies

Fuel cells are advanced electrochemical devices that directly convert the chemical energy of a fuel into electrical energy through electrochemical reactions. Unlike conventional power generation systems that rely on combustion processes, fuel cells generate electricity without burning fuel, thereby avoiding many of the efficiency limitations and environmental problems associated with fossil-fuel-based technologies. Because of their high efficiency, low emissions, quiet operation, and fuel flexibility, fuel cells are increasingly considered one of the most promising technologies for future sustainable energy systems. They are being actively explored for applications in transportation, stationary power generation, portable electronics, aerospace systems, and renewable energy integration. Hydrogen is the most commonly used fuel in fuel cells because it possesses high energy content and produces water as the primary reaction by-product. When hydrogen reacts electrochemically with oxygen, electricity and heat are generated continuously as long as reactants are supplied to the system. This ability to provide continuous power generation distinguishes fuel cells from conventional batteries, which store a limited amount of chemical energy internally.

### 3.1. Basic Working Principles

The operation of fuel cells is based on electrochemical energy conversion processes involving oxidation and reduction reactions. In a typical hydrogen fuel cell, hydrogen fuel is supplied to the anode, while oxygen or air is supplied to the cathode. These reactants participate in separate electrochemical reactions at the two electrodes, allowing the direct conversion of chemical energy into electrical energy with high efficiency and minimal environmental impact. The overall electrochemical reaction occurring in a hydrogen fuel cell can be expressed as:

2*H*_2_ + *O*_2_ → 2*H*_2_*O* + *electrical energy* + *heat* (22)

This reaction demonstrates that hydrogen and oxygen combine to produce water, electricity, and heat. Unlike combustion engines, where chemical energy is first converted into thermal energy and then into mechanical energy, fuel cells bypass intermediate thermal processes and directly generate electricity. This direct conversion significantly improves overall efficiency and reduces energy losses. At the anode, hydrogen molecules undergo oxidation and split into hydrogen ions and electrons:

*H*_2_ → 2*H*^+^ + 2*e*^−^ (23)

The electrons generated at the anode cannot pass through the electrolyte and are therefore forced to travel through an external electrical circuit. This movement of electrons creates electric current that can be used to power electrical devices. Simultaneously, hydrogen ions move through the electrolyte toward the cathode. At the cathode, oxygen molecules react with the incoming electrons and hydrogen ions to produce water:

½ *O*_2_ + 2*H*^+^ + 2*e*^−^ → *H*_2_*O* (24)

These electrochemical reactions continue continuously as long as fuel and oxidant are supplied, enabling stable electricity generation. Since the primary by-products are water and heat, fuel cells are regarded as environmentally friendly energy conversion systems, especially when hydrogen is produced from renewable energy sources. Fuel cells generally exhibit electrical efficiencies ranging from 40% to 70%, which is significantly higher than many conventional internal combustion engines and thermal power plants. Furthermore, when waste heat generated during operation is recovered and utilized in combined heat and power (CHP) systems, total system efficiencies can exceed 80%. This high efficiency makes fuel cells highly attractive for both stationary and mobile energy applications.

A typical fuel cell consists of several important components, including the anode, cathode, electrolyte, catalyst layers, gas diffusion layers, and bipolar plates. Each component performs a specialized role that contributes to the efficient operation of the fuel cell system. The anode serves as the negative electrode where hydrogen oxidation takes place. Hydrogen gas supplied to the anode diffuses through porous gas diffusion layers and reaches the catalyst surface, where it dissociates into protons and electrons. The catalyst facilitates this reaction by lowering the activation energy required for hydrogen oxidation. Platinum-based catalysts are commonly used in low-temperature fuel cells because of their excellent catalytic activity and high reaction efficiency. The cathode functions as the positive electrode where oxygen reduction reactions occur. Oxygen supplied from air reacts with electrons arriving through the external circuit and ions transported through the electrolyte to form water. The oxygen reduction reaction is generally slower than hydrogen oxidation and often represents one of the major causes of energy losses within fuel cells. Consequently, considerable research efforts are focused on improving cathode catalysts and enhancing oxygen reduction kinetics.

The electrolyte is one of the most critical fuel cell components because it selectively conducts ions between electrodes while preventing direct mixing of fuel and oxidant gases. Different fuel cell technologies utilize different electrolytes depending on operating conditions and intended applications. For example, proton exchange membrane fuel cells (PEMFCs) use polymer membranes that conduct hydrogen ions, whereas solid oxide fuel cells (SOFCs) use ceramic electrolytes capable of conducting oxygen ions at high temperatures. Catalyst layers are deposited on the electrode surfaces to accelerate electrochemical reactions without being consumed during operation. Although platinum-based catalysts provide high performance, their high cost and limited availability significantly increase fuel cell expenses. Researchers are therefore developing alternative catalyst materials based on transition metals, nanostructures, and carbon-supported composites to reduce costs while maintaining high catalytic activity.

Gas diffusion layers are porous conductive materials positioned between the catalyst layers and bipolar plates. These layers facilitate uniform gas distribution, electron conduction, water management, and mechanical support. Proper gas diffusion is essential for maintaining efficient reactant transport and preventing localized concentration losses within the fuel cell. Bipolar plates separate individual fuel cells within a stack and provide pathways for gas flow, heat transfer, and electrical conduction. Since a single fuel cell produces relatively low voltage, multiple cells are connected in series to form a fuel cell stack capable of delivering practical power outputs for commercial applications. Bipolar plates therefore play an essential role in maintaining structural integrity and ensuring efficient stack operation.

The overall operation mechanism of a fuel cell involves a coordinated sequence of processes including fuel supply, electrochemical oxidation, ion transport, electron flow, oxygen reduction, and water formation. Hydrogen enters the anode side of the cell and dissociates into ions and electrons at the catalyst surface. Electrons travel through the external circuit to generate electricity, while ions pass through the electrolyte toward the cathode. At the cathode, oxygen reacts with electrons and ions to produce water and heat. This continuous electrochemical process enables stable and efficient power generation without combustion. In summary, fuel cells are highly efficient electrochemical energy conversion systems that directly transform chemical energy into electrical energy through controlled oxidation–reduction reactions. Their operation depends on the interaction of specialized components such as electrodes, electrolytes, catalysts, and gas diffusion layers to facilitate ion transport and electron flow. Because of their high efficiency, low emissions, and versatility, fuel cells are expected to play a major role in future clean energy systems and global decarbonization efforts.

### 3.2. Classification of Fuel Cells

Fuel cells are classified according to the type of electrolyte they employ, the ionic species transported through the electrolyte, operating temperature range, and fuel compatibility. The electrolyte plays a fundamental role in determining the electrochemical reactions, efficiency, catalyst requirements, durability, and practical applications of the fuel cell system. Different fuel cell technologies have therefore been developed to satisfy the diverse energy requirements of transportation, stationary power generation, portable electronics, aerospace systems, and industrial applications. Some fuel cells are designed for rapid startup and low-temperature operation suitable for automotive use, while others operate at very high temperatures to maximize efficiency and fuel flexibility for stationary power production. A comparative overview of the major fuel cell technologies, including their electrolytes, operating conditions, advantages, limitations, and commercial applications, is summarized in Table 3.

#### 3.2.1. Proton Exchange Membrane Fuel Cells (PEMFCs)

**Figure 4:**
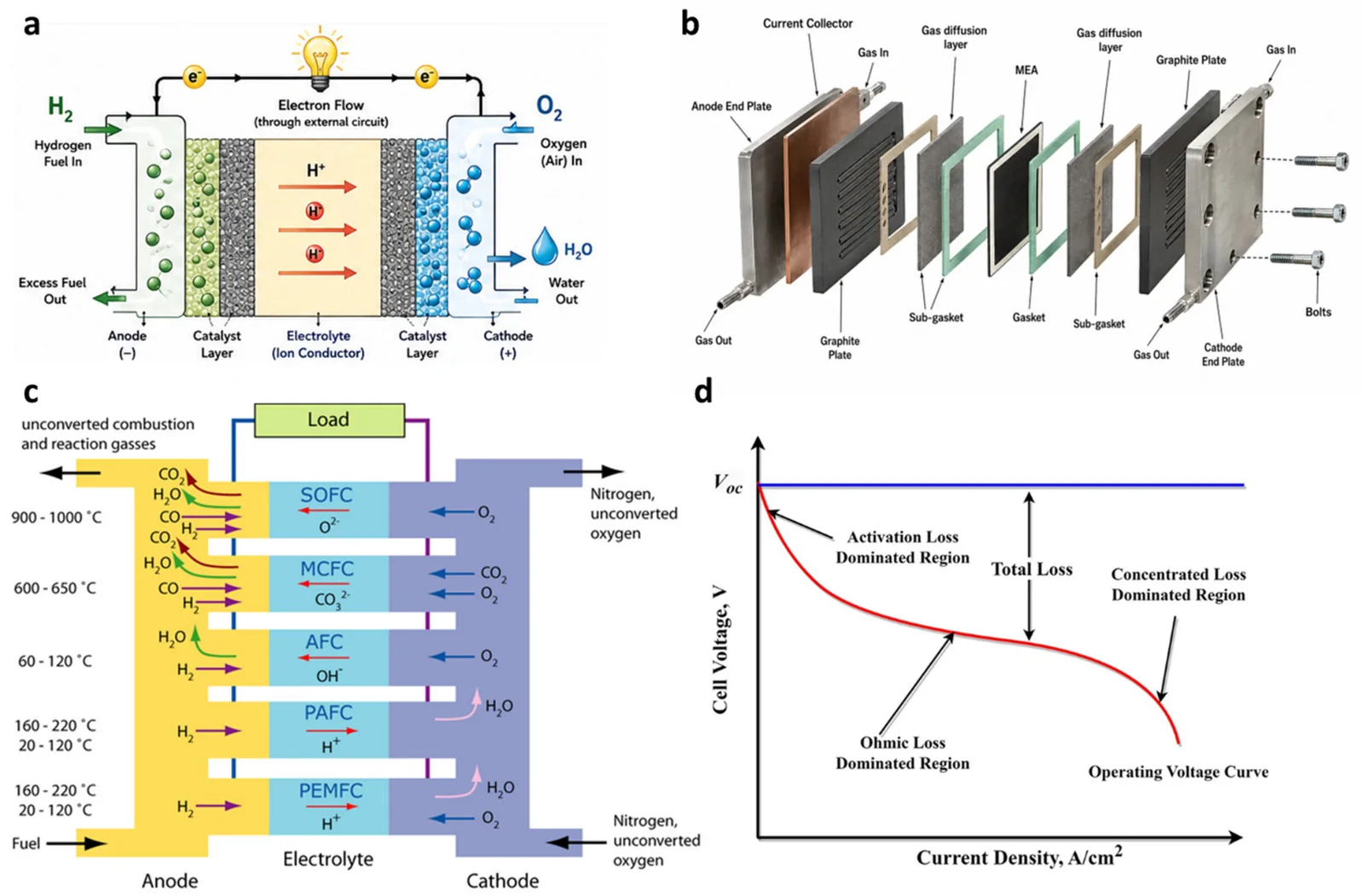
(**a**) Basic working principle of a hydrogen fuel cell; (**b**) major fuel cell stack components [49]; (**c**) classification of fuel cell technologies [50]; (**d**) typical polarization losses affecting fuel cell performance. The illustration was generated with the assistance of OpenAI.

Proton Exchange Membrane Fuel Cells (PEMFCs), also known as Polymer Electrolyte Membrane Fuel Cells, are among the most commercially advanced and widely researched fuel cell technologies. PEMFCs utilize a solid polymer membrane as the electrolyte, which selectively conducts protons from the anode to the cathode while blocking electrons and preventing the direct mixing of hydrogen and oxygen gases. These fuel cells generally operate at relatively low temperatures, typically between 60 °C and 80 °C, allowing rapid startup, compact system design, and high-power density. As shown in Figure 4a–d, fuel cell performance is governed by a combination of electrochemical reaction mechanisms, component architecture, electrolyte selection, and polarization losses that collectively determine efficiency and durability.

In PEMFCs, hydrogen supplied to the anode undergoes oxidation to produce protons and electrons:

*H*_2_ → 2*H*^+^ + 2*e*^−^ (25)

The released electrons travel through an external electrical circuit, generating electric current, while protons migrate through the proton-conducting membrane toward the cathode. At the cathode, oxygen reacts with protons and electrons to produce water:

½ *O*_2_ + 2*H*^+^ + 2*e*^−^ → *H*_2_*O* (26)

The overall reaction occurring in PEMFCs is:

2*H*_2_ + *O*_2_ → 2*H*_2_*O* (27)

PEMFCs offer several important advantages, including high electrical efficiency, low operating temperature, rapid dynamic response, and environmentally friendly operation with water as the primary by-product. These characteristics make PEMFCs highly suitable for fuel cell electric vehicles, portable electronics, backup power systems, and distributed energy applications. However, PEMFCs rely heavily on expensive platinum-based catalysts to facilitate electrochemical reactions, significantly increasing system cost. Furthermore, the membranes require careful water management because insufficient hydration reduces proton conductivity, whereas excessive water accumulation may flood the electrodes and hinder gas transport. PEMFCs are also highly sensitive to fuel impurities such as carbon monoxide, which can poison catalysts and degrade performance.

#### 3.2.2. Solid Oxide Fuel Cells (SOFCs)

Solid Oxide Fuel Cells (SOFCs) are high-temperature fuel cells that employ solid ceramic electrolytes capable of conducting oxygen ions. SOFCs typically operate at temperatures between 600 °C and 1000 °C, which enables rapid electrochemical reaction kinetics and eliminates the need for precious metal catalysts. The high operating temperature also allows internal fuel reforming and the direct utilization of various hydrocarbon fuels such as natural gas, methane, biogas, and syngas. In SOFCs, oxygen molecules at the cathode gain electrons and form oxygen ions:

½ *O*_2_ + 2*e*^−^ → *O*^−2^ (28)

The oxygen ions migrate through the ceramic electrolyte toward the anode, where they react with hydrogen fuel to produce water and release electrons:

*H*_2_ + *O*^−2^ → *H*_2_*O* + 2*e*^−^ (29)

SOFCs possess exceptionally high electrical efficiencies and are particularly attractive for stationary power generation and combined heat and power (CHP) systems. Because waste heat generated during operation can be recovered and utilized, total system efficiencies may exceed 80%. Another important advantage is fuel flexibility, which reduces dependence on pure hydrogen infrastructure. Despite these benefits, SOFCs face several technical challenges associated with high-temperature operation. Thermal expansion mismatch among components can create mechanical stresses and material degradation. Long startup times, sealing difficulties, and electrode degradation also limit durability and increase system complexity. Nevertheless, continuous advancements in ceramic materials, electrode engineering, and thermal management technologies are improving SOFC performance and reliability.

#### 3.2.3. Proton Ceramic Fuel Cells (PCFCs)

Proton Ceramic Fuel Cells (PCFCs) are an emerging class of intermediate-temperature fuel cells that combine favorable characteristics of both PEMFCs and SOFCs. PCFCs employ ceramic electrolytes capable of conducting protons at temperatures generally ranging between 400 °C and 700 °C. These intermediate operating temperatures allow faster electrochemical kinetics than low-temperature PEMFCs while reducing the severe thermal degradation problems associated with traditional SOFCs. The electrochemical reactions in PCFCs resemble those in PEMFCs. At the anode, hydrogen is oxidized into protons and electrons:

*H*_2_ → 2*H*^+^ + 2*e*^−^ (30)

At the cathode, oxygen reacts with protons and electrons to form water:

½ *O*_2_ + 2*H*^+^ + 2*e*^−^ → *H*_2_*O* (31)

PCFCs offer promising advantages such as high efficiency, improved fuel flexibility, reduced thermal stress, and potentially lower material costs compared to conventional SOFCs. The generation of water at the cathode side also minimizes fuel dilution and can improve fuel utilization efficiency. However, PCFC technology is still under active development, and challenges remain regarding electrolyte conductivity, chemical stability, electrode compatibility, and long-term durability. Continued research into proton-conducting ceramic materials is expected to accelerate the commercialization of PCFC systems.

#### 3.2.4. Alkaline Fuel Cells (AFCs)

Alkaline Fuel Cells (AFCs) are among the earliest developed fuel cell technologies and have historically been used in aerospace applications, particularly in spacecraft and space missions. AFCs utilize alkaline electrolytes such as potassium hydroxide (KOH), which conduct hydroxide ions between the electrodes. At the anode, hydrogen reacts with hydroxide ions to produce water and electrons:

*H*_2_ + 2*OH*^−^ → 2*H*_2_*O* + 2*e*^−^ (32)

At the cathode, oxygen reacts with water and electrons to regenerate hydroxide ions:

½ *O*_2_ + *H*_2_*O* + 2*e*^−^ → 2*OH*^−^ (33)

AFCs exhibit high electrical efficiency and relatively fast oxygen reduction kinetics under alkaline conditions. Another advantage is the possibility of using non-precious metal catalysts, reducing overall system cost. However, AFCs are extremely sensitive to carbon dioxide contamination because CO_2_ reacts with the alkaline electrolyte to form carbonates, decreasing ionic conductivity and degrading performance. Consequently, AFC systems require highly purified hydrogen and oxygen supplies, which limits their broader commercial deployment.

#### 3.2.5. Molten Carbonate Fuel Cells (MCFCs)

Molten Carbonate Fuel Cells (MCFCs) are high-temperature fuel cells that employ molten carbonate salts as electrolytes and operate at temperatures around 650 °C. At these elevated temperatures, carbonate ions serve as the charge carriers within the electrolyte.

At the cathode, oxygen and carbon dioxide combine with electrons to form carbonate ions:

½ *O*_2_ + *CO*_2_ + 2*e*^−^ → *CO*_3_^2−^ (34)

The carbonate ions migrate through the electrolyte to the anode, where they react with hydrogen fuel:

*H*_2_ + *CO*_3_^2−^ → *H*_2_*O* + *CO*_2_ + 2*e*^−^ (35)

MCFCs offer high efficiency and excellent fuel flexibility, allowing the direct utilization of natural gas and other hydrocarbon fuels. Their high operating temperature enables internal reforming and efficient waste heat recovery for cogeneration systems. However, the corrosive molten electrolyte and elevated temperatures accelerate material degradation and create sealing and durability challenges. Consequently, MCFCs are primarily suited for large-scale stationary power generation applications.

#### 3.2.6. Direct Methanol Fuel Cells (DMFCs)

Direct Methanol Fuel Cells (DMFCs) are specialized low-temperature fuel cells that directly utilize liquid methanol as fuel without requiring external reforming systems. This feature simplifies fuel storage and handling compared to hydrogen-based systems, making DMFCs attractive for portable electronics and small-scale power devices. At the anode, methanol reacts with water to produce carbon dioxide, protons, and electrons:

*CH*_3_*OH* + *H*_2_*O* → *CO*_2_ + 6*H*^+^ + 6*e*^−^ (36)

At the cathode, oxygen reacts with protons and electrons to form water:

(37) $$\frac{3}{2}O_{2}+6H^{+}+6e^{-}\to 3H_{2}O$$

DMFCs offer advantages such as simple fuel storage, high energy density, and convenient liquid fuel handling. However, methanol crossover through the membrane significantly reduces efficiency and causes catalyst poisoning. Additionally, DMFCs generally exhibit lower power densities than PEMFCs, limiting their use in high-power applications.

#### 3.2.7. Anion Exchange Membrane Fuel Cells (AEMFCs)

Anion Exchange Membrane Fuel Cells (AEMFCs) are emerging fuel cell technologies that combine features of alkaline fuel cells and PEMFCs. These systems utilize solid polymer membranes capable of conducting hydroxide ions under alkaline conditions. At the cathode, oxygen reacts with water and electrons to form hydroxide ions:

*O*_2_ + 2*H*_2_*O* + 4*e*^−^ → 4*OH*^−^ (38)

The hydroxide ions migrate through the membrane to the anode, where hydrogen reacts to form water and electrons:

2*H*_2_ + 4*OH*^−^ → 4*H*_2_*O* + 4*e*^−^ (39)

AEMFCs offer several promising advantages, including reduced dependence on platinum catalysts, lower material costs, and improved oxygen reduction kinetics in alkaline environments. Nevertheless, membrane stability, carbonate formation, and long-term durability remain major technical challenges that must be overcome before widespread commercialization.

### 3.3. Thermodynamics and Electrochemical Kinetics

The operation and performance of fuel cells are fundamentally governed by thermodynamic principles and electrochemical reaction kinetics. Thermodynamics determines the maximum theoretical energy conversion efficiency and reversible cell voltage, whereas electrochemical kinetics controls actual reaction rates, polarization losses, and practical operating performance. One of the most important thermodynamic relationships in fuel cell analysis is the Nernst equation, which describes how cell voltage varies with temperature, pressure, and reactant concentration [51]. The generalized Nernst equation is expressed as:

(40) $$E=E^{0}-\frac{\mathrm{RT}}{\mathrm{nF}}\mathrm{lnQ}$$

where E is the cell potential, E^0^ is the standard electrode potential, R is the universal gas constant, T is temperature, n is the number of electrons transferred, F is Faraday’s constant, and Q is the reaction quotient. The Nernst equation demonstrates that fuel cell voltage increases with higher reactant concentrations and pressures while being influenced by temperature changes. In practical operation, the actual fuel cell voltage is always lower than the theoretical voltage because of irreversible losses known as polarization losses or overpotentials. These losses are generally categorized into activation losses, ohmic losses, and concentration losses. Activation losses arise from the energy barrier associated with electrochemical reactions at the electrode surfaces. These losses are especially significant at low current densities and are strongly dependent on catalyst activity and reaction kinetics. The oxygen reduction reaction at the cathode is generally much slower than hydrogen oxidation and therefore contributes substantially to activation losses. Ohmic losses result from resistance to ion transport within the electrolyte and electron flow through electrodes and interconnects [52]. These losses increase linearly with current and can be represented by Ohm’s law:

*V*_ohmic_ = *IR* (41)

where I is current and R is resistance. Reducing membrane thickness and improving material conductivity are common strategies for minimizing ohmic losses.

Concentration losses occur at high current densities when reactant transport becomes insufficient to sustain electrochemical reactions. Inadequate diffusion of hydrogen or oxygen toward the catalyst surface reduces reactant concentration and lowers cell voltage [53]. Electrochemical reaction mechanisms are also critically important in determining fuel cell and electrolyzer performance. The Hydrogen Oxidation Reaction (HOR) occurs at the fuel cell anode:

*H*_2_ → 2*H*^+^ + 2*e*^−^ (42)

The Hydrogen Evolution Reaction (HER), which is the reverse process, occurs during electrolysis:

2*H*^+^ + 2*e*^−^ → *H*_2_ (43)

The Oxygen Reduction Reaction (ORR), occurring at the fuel cell cathode, is expressed as:

*O*_2_ + 4*H*^+^ + 4*e*^−^ → 2*H*_2_*O* (44)

Meanwhile, the Oxygen Evolution Reaction (OER) occurs during water electrolysis:

2*H*_2_*O* → *O*_2_ + 4*H*^+^ + 4*e*^−^ (45)

Among these reactions, ORR and OER are kinetically sluggish and require highly active catalysts to improve reaction rates and reduce energy losses. Consequently, catalyst development remains one of the most important research areas in hydrogen energy and fuel cell technologies [54]. Overall, fuel cell technologies exhibit diverse operational characteristics depending on electrolyte type, operating temperature, and ionic conduction mechanisms. Their performance is fundamentally governed by thermodynamics, electrochemical kinetics, and reaction mechanisms that collectively determine efficiency, durability, and commercial viability in future clean energy systems.

## 4. Advanced Materials for Hydrogen Energy and Fuel Cells

The rapid advancement of hydrogen energy technologies and fuel cell systems is fundamentally dependent on the development of highly efficient, durable, and economically viable functional materials. Although substantial progress has been achieved in hydrogen production, storage, and electrochemical conversion technologies, material-related limitations continue to represent one of the most significant barriers to large-scale commercialization [55]. In practical systems, the performance of electrolyzers and fuel cells is strongly governed by the catalytic activity of electrode materials, ionic conductivity of electrolytes, interfacial charge-transfer characteristics, thermal and chemical stability, corrosion resistance, and long-term degradation behavior under realistic operating conditions [56]. In recent years, major breakthroughs in nanotechnology, computational materials science, operando characterization, defect engineering, and interface tailoring have accelerated the discovery of advanced materials for hydrogen energy systems. In particular, the emergence of high-entropy oxides, MXenes, single-atom catalysts, heterostructured nanomaterials, proton-conducting ceramics, and hybrid composite electrolytes has created new opportunities for improving electrochemical efficiency while reducing dependence on scarce noble metals [57]. Nevertheless, despite remarkable laboratory-scale catalytic activities reported for many advanced materials, several critical challenges remain unresolved. Many catalyst systems suffer from poor long-term durability, structural instability, limited scalability, high synthesis complexity, and insufficient performance under industrial current densities. Furthermore, discrepancies between half-cell laboratory measurements and full-cell operational conditions often complicate realistic evaluation of material performance. Another major issue concerns the growing gap between academic research and practical commercialization. A large number of studies focus primarily on achieving low overpotentials and high current densities under idealized laboratory conditions, whereas industrial implementation requires simultaneous optimization of cost, durability, manufacturability, catalyst loading, thermal stability, and operational reliability [58]. Consequently, next-generation hydrogen materials research increasingly emphasizes integrated activity–stability–scalability relationships rather than isolated electrochemical metrics.

In addition, the electrochemical behavior of hydrogen technologies is strongly influenced by dynamic surface reconstruction, interfacial charge redistribution, defect chemistry, grain-boundary conduction, and multiscale transport phenomena occurring during operation. Advanced operando and in situ characterization tools such as synchrotron X-ray spectroscopy, transmission electron microscopy (TEM), neutron diffraction, Raman spectroscopy, and X-ray photoelectron spectroscopy (XPS) have therefore become indispensable for understanding catalytic mechanisms and degradation pathways at the atomic scale [59]. This section critically discusses recent advances in advanced materials for hydrogen energy and fuel cells, including electrocatalysts, electrolytes, electrodes, interconnect materials, and interface-engineering strategies. Particular attention is devoted to structure–property relationships, degradation mechanisms, scalability limitations, and future research directions for commercially viable hydrogen technologies.

### 4.1. Electrocatalysts for Hydrogen Production and Fuel Cells

**Figure 5:**
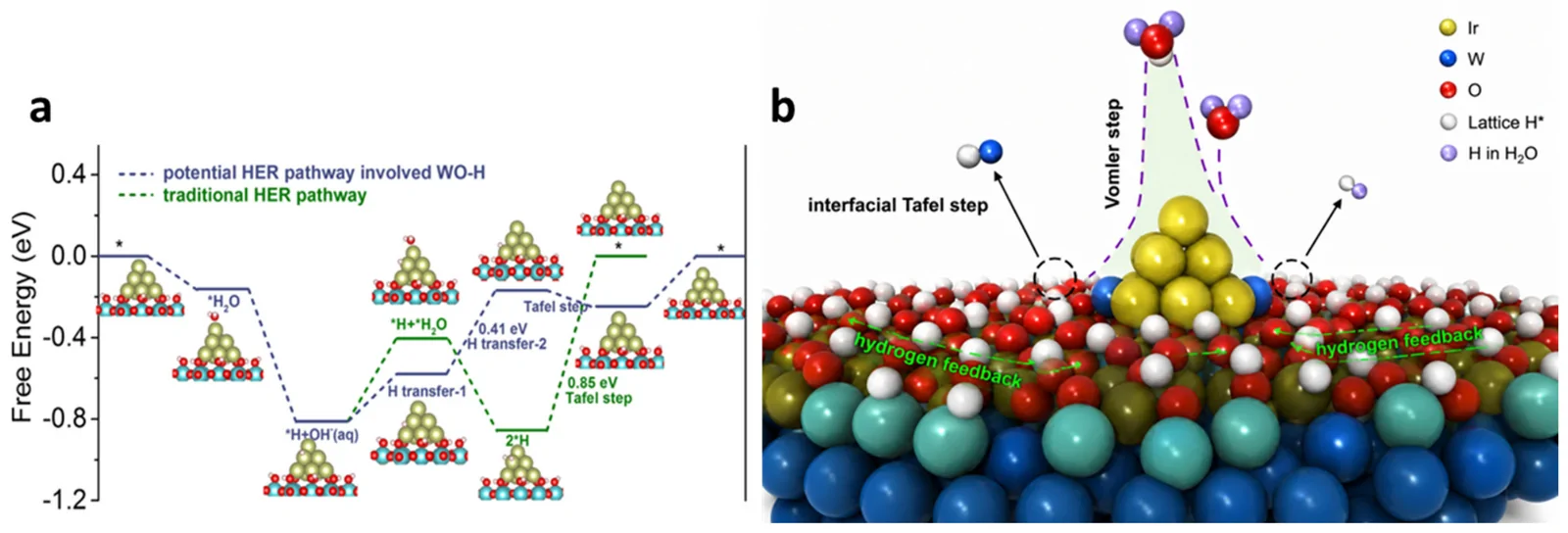
(**a**) Free-energy profiles of the WO-H mediated and traditional HER pathways. (**b**) Schematic illustration of interfacial hydrogen transfer and HER steps [63].

Electrocatalysts are among the most critical components in hydrogen energy systems because they directly determine reaction kinetics, energy efficiency, operational stability, and overall system cost. Electrochemical reactions such as hydrogen evolution reaction (HER), hydrogen oxidation reaction (HOR), oxygen reduction reaction (ORR), and oxygen evolution reaction (OER) involve complex multistep proton-coupled electron-transfer mechanisms associated with substantial activation energy barriers [60]. Consequently, highly active catalysts are required to accelerate charge-transfer processes and minimize polarization losses. Among these reactions, ORR and OER are particularly sluggish because they involve multiple intermediate adsorption and desorption steps. In practical fuel cell and electrolyzer systems, these kinetic limitations significantly reduce energy-conversion efficiency and increase operational voltage losses. Therefore, the development of highly active electrocatalysts remains one of the central challenges in hydrogen energy research. Traditionally, noble-metal-based catalysts such as Pt, IrO_2_, and RuO_2_ have demonstrated the highest catalytic activity because of their optimized adsorption energies and favorable electronic structures [61]. However, their widespread commercialization is constrained by scarcity, high cost, susceptibility to degradation, and limited long-term sustainability. As a result, recent research efforts increasingly focus on developing earth-abundant alternatives capable of delivering comparable catalytic performance with improved durability and reduced cost. Importantly, catalytic activity alone is insufficient for practical implementation. Many recently reported catalysts exhibit excellent half-cell electrochemical performance but fail under realistic industrial operating conditions due to catalyst dissolution, particle agglomeration, carbon-support corrosion, or interfacial instability [62]. Therefore, future catalyst development requires a comprehensive balance between activity, stability, conductivity, scalability, and manufacturability. As illustrated in Figure 5. Advanced electrocatalysts including noble metal catalysts and non-noble metal catalysts, perovskite oxides, high-entropy oxides, MXenes, single atom catalysts, and defect engineered catalysts, play crucial rule in enhancing the kinetics of HER, OER, ORR, and HOR for efficient hydrogen conversion [63].

#### 4.1.1. Noble Metal Catalysts

Noble-metal catalysts remain the benchmark materials for hydrogen-energy-related electrochemical reactions because of their exceptional catalytic activity and favorable reaction kinetics. Platinum-based catalysts exhibit nearly ideal hydrogen adsorption energies, making them highly efficient for HER and HOR processes. Similarly, iridium oxide (IrO_2_) and ruthenium oxide (RuO_2_) are considered state-of-the-art catalysts for oxygen evolution reactions under acidic conditions, particularly in proton exchange membrane water electrolyzers [45]. Pt/C catalysts continue to dominate commercial PEM fuel cell systems because of their high ORR activity, excellent electrical conductivity, and superior low-temperature performance. The exceptional catalytic activity of platinum originates from its optimized d-band electronic structure, which provides near-optimal adsorption strength for hydrogen and oxygen-containing intermediates. However, despite these advantages, platinum-group metals face several severe economic and technical limitations. First, noble metals are among the rarest elements on Earth, resulting in extremely high material costs and vulnerable supply chains. Platinum and iridium production is geographically concentrated in a limited number of countries, creating potential geopolitical and economic risks for large-scale hydrogen deployment. Second, noble-metal catalysts are highly susceptible to degradation under realistic operating conditions [64]. Platinum nanoparticles often undergo dissolution, agglomeration, Ostwald ripening, and carbon-support corrosion during fuel cell startup–shutdown cycles and potential fluctuations. Furthermore, IrO_2_ and RuO_2_ catalysts exhibit significant instability during long-term OER operation because of catalyst dissolution under highly oxidative anodic potentials [65]. Ru-based catalysts generally possess higher intrinsic activity than iridium but suffer from inferior corrosion resistance and structural durability.

To address these limitations, extensive efforts have been devoted to improving atomic utilization efficiency through nanoscale engineering strategies such as alloying, strain engineering, core–shell architectures, hollow nanostructures, and single-atom dispersion. Pt-Ni, Pt-Co, and Pt-Fe alloys have demonstrated enhanced ORR performance due to modified electronic structures and optimized oxygen-binding energies [66]. Likewise, ultrathin Ir nanoframes and atomically dispersed Ru catalysts have shown improved catalytic efficiency with reduced noble-metal loading. Nevertheless, many advanced noble-metal nanostructures remain difficult to synthesize reproducibly at industrial scale. In addition, laboratory-reported performance metrics are often obtained under idealized conditions that differ significantly from practical membrane-electrode assembly (MEA) environments. Consequently, the scalability and industrial reproducibility of many advanced Pt-group catalysts remain uncertain. Another critical challenge involves catalyst–support interactions [67]. Conventional carbon supports used in PEM fuel cells suffer severe corrosion under high anodic potentials, leading to catalyst detachment and performance degradation. Alternative conductive supports such as doped metal oxides, graphitized carbons, and titanium-based materials have therefore attracted increasing attention. Although noble-metal catalysts continue to provide the highest electrochemical performance, their long-term sustainability and economic feasibility remain major obstacles for global hydrogen commercialization.

#### 4.1.2. Non-Noble Metal Catalysts

The high cost and limited availability of platinum-group metals have motivated extensive research into non-noble-metal electrocatalysts composed of earth-abundant transition metals such as Ni, Fe, Co, Mn, and Cu. These materials offer substantial economic advantages and possess highly tunable electronic structures that can be engineered for improved catalytic performance. Transition-metal oxides, sulfides, phosphides, nitrides, carbides, and hydroxides have demonstrated considerable promise for HER, OER, and ORR applications. In particular, nickel–iron layered double hydroxides (NiFe-LDHs) are currently regarded among the most active alkaline OER catalysts because of synergistic electronic interactions between Ni and Fe active sites [68]. Despite these advances, non-noble catalysts generally suffer from lower intrinsic conductivity and inferior acidic stability compared with noble-metal systems. Many transition-metal compounds exhibit severe dissolution under acidic environments, limiting their application in PEM electrolyzers and PEM fuel cells. Perovskite oxides with the general formula ABO3 have attracted enormous research interest because of their flexible crystal structures and tunable oxygen-vacancy chemistry. Their catalytic performance can be adjusted by manipulating B-site transition-metal composition, oxygen nonstoichiometry, and orbital occupancy. Perovskites such as LaNiO_3_, LaCoO_3_, and Ba_0.5_Sr_0.5_Co_0.8_Fe_0.2_O_3−δ_ have demonstrated promising ORR and OER activities [69]. However, despite excellent laboratory-scale performance, many perovskites experience surface amorphization, cation segregation, and structural instability during prolonged electrochemical operation. Surface reconstruction phenomena often alter the actual catalytic active sites, complicating mechanistic interpretation.

Spinel oxides such as Co_3_O_4_, NiCo_2_O_4_, and MnCo_2_O_4_ also exhibit favorable catalytic properties because of mixed valence states and improved electron-transfer pathways. Nevertheless, spinels frequently suffer from limited conductivity and insufficient stability under industrial operating conditions [70]. High-entropy oxides (HEOs) have recently emerged as a highly promising catalyst family because their multicomponent compositions create unique configurational entropy effects that stabilize crystal structures and enhance catalytic durability [71]. The synergistic interaction among multiple cations can improve oxygen-vacancy formation, electronic conductivity, and corrosion resistance. However, the catalytic mechanisms governing HEO performance remain poorly understood because of their structural complexity. Moreover, reproducible large-scale synthesis of compositionally homogeneous high-entropy materials remains technologically challenging [72]. Importantly, many non-noble catalysts demonstrate excellent performance only in alkaline environments, whereas acidic electrolysis systems still heavily rely on noble metals. Bridging this alkaline–acidic stability gap remains one of the most important challenges for future catalyst development.

#### 4.1.3. Emerging Catalytic Materials

Emerging catalytic materials such as MXenes, single-atom catalysts (SACs), carbon-based nanomaterials, and defect-engineered structures have recently attracted exceptional attention because they provide unprecedented opportunities for atomic-level catalyst engineering. MXenes are a rapidly growing family of two-dimensional transition-metal carbides and nitrides with the general formula M_n+1_X_n_T_x_ [73]. These materials possess exceptional electrical conductivity, hydrophilic surfaces, tunable terminations, and layered structures that facilitate rapid charge transport and enhanced electrolyte accessibility. Among MXenes, Ti_3_C_2_T_x_ has been extensively investigated for HER and electrocatalytic support applications. Surface functional groups such as -OH, -F, and -O strongly influence catalytic behavior and interfacial interactions [74]. However, MXenes suffer from severe oxidation instability under ambient and electrochemical conditions, which significantly limits long-term operational durability.

Single-atom catalysts represent another transformative development in electrocatalysis because isolated metal atoms maximize atomic utilization efficiency and create highly uniform active sites. Fe-N-C and Co-N-C catalysts have shown ORR activity approaching that of Pt-based catalysts in alkaline fuel cells. Nevertheless, several critical challenges remain unresolved [75]. Atomically dispersed metals are often thermodynamically unstable and tend to aggregate under prolonged electrochemical operation. Furthermore, identifying the true catalytic active sites in SAC systems remains controversial because of dynamic coordination changes during operation. Carbon-based nanomaterials including graphene, carbon nanotubes, porous carbons, and heteroatom-doped carbon frameworks continue to attract major attention because of their high conductivity, large surface area, and tunable electronic structures. Nitrogen-doped graphene and carbon nanotubes can significantly improve ORR activity by modifying charge distribution and creating catalytically active defect sites.

Defect engineering has emerged as one of the most powerful approaches for enhancing catalytic activity. Oxygen vacancies, sulfur vacancies, lattice strain, heterointerfaces, and edge defects can strongly alter adsorption energies and reaction pathways. In many cases, defect-rich catalysts exhibit significantly improved HER and OER kinetics [76]. However, excessive defect concentrations may destabilize crystal structures and accelerate degradation processes. Additionally, controlled synthesis of defect-rich materials with reproducible defect distributions remains technically challenging.

### 4.2. Electrolyte Materials

Electrolyte materials play a decisive role in determining ionic conductivity, operating temperature, fuel compatibility, electrochemical efficiency, and long-term durability of hydrogen energy systems and fuel cells [77]. The electrolyte functions as an ion-conducting medium while simultaneously preventing direct mixing of fuel and oxidant gases. Consequently, electrolyte properties strongly influence internal resistance, thermal stability, chemical compatibility, and overall device lifetime. An ideal electrolyte should possess high ionic conductivity, negligible electronic conductivity, excellent chemical and mechanical stability, low gas permeability, and strong compatibility with electrode materials. However, achieving all these properties simultaneously remains extremely challenging because improvements in one parameter often compromise another. Recent research has therefore focused on developing advanced polymer membranes, proton-conducting ceramics, oxygen-ion-conducting electrolytes, and hybrid composite systems capable of operating efficiently under diverse electrochemical environments [78]. Overall, emerging catalytic materials demonstrate extraordinary potential for next-generation hydrogen technologies. Nevertheless, future research must increasingly prioritize stability, industrial scalability, catalyst loading reduction, and practical device integration rather than focusing exclusively on record-breaking electrochemical metrics.

#### 4.2.1. Polymer Electrolyte Membranes

Polymer electrolyte membranes are widely used in PEM fuel cells and PEM electrolyzers because of their high proton conductivity and low-temperature operation. Nafion remains the most commercially successful membrane owing to its excellent chemical stability and proton transport properties. However, Nafion-based systems suffer from several important limitations. Their proton conductivity strongly depends on hydration level, making water management a critical operational challenge. Membrane dehydration at elevated temperatures significantly reduces conductivity, whereas excessive water accumulation can flood catalyst layers and block gas diffusion [79]. Furthermore, Nafion membranes are expensive because of fluorinated polymer synthesis and exhibit limited operating temperatures below approximately 80–90 °C. Low-temperature operation also increases catalyst poisoning sensitivity and limits electrode kinetics. Alternative hydrocarbon-based membranes such as sulfonated polyether ether ketone (SPEEK), polybenzimidazole (PBI), and polyarylene ether membranes have therefore attracted increasing attention because of lower cost and improved thermal stability [80]. Nevertheless, many hydrocarbon membranes suffer from inferior chemical durability and mechanical stability compared with perfluorinated systems. Membrane degradation caused by radical attack, swelling, and mechanical fatigue remains a major challenge for long-term operation.

#### 4.2.2. Ceramic Proton Conductors

Proton-conducting ceramic electrolytes have emerged as highly promising materials for intermediate-temperature fuel cells and electrolyzers because they combine high thermal stability with efficient proton transport. Perovskite-type proton conductors such as BaZrO_3_, BaCeO_3_, and BaCeZrO_3_ based ceramics exhibit substantial proton conductivity at intermediate temperatures between 400 and 700 °C [81]. Compared with conventional SOFC electrolytes, proton-conducting ceramics enable lower operating temperatures while maintaining favorable electrochemical kinetics. However, proton-conducting ceramics face significant challenges related to grain-boundary resistance, sintering difficulty, chemical instability in CO_2_-containing atmospheres, and electrode compatibility [82]. BaCeO^−3^ based materials often exhibit superior proton conductivity but poor chemical stability, whereas BaZrO_3_ systems possess excellent stability but inferior sintering characteristics and grain-boundary conductivity. Current research therefore focuses on compositional engineering, grain-boundary modification, and interface optimization to balance conductivity and stability.

#### 4.2.3. Oxygen-Ion Conducting Electrolytes

Oxygen-ion-conducting electrolytes are essential components in conventional SOFC systems. Yttria-stabilized zirconia (YSZ) remains the benchmark oxygen-ion conductor because of its excellent thermal stability and mechanical durability. Nevertheless, YSZ requires very high operating temperatures above 800 °C to achieve sufficient ionic conductivity, leading to severe thermal degradation, material incompatibility, and high system costs [83]. Alternative electrolytes such as gadolinium-doped ceria (GDC) and lanthanum gallate-based materials have therefore been investigated for intermediate-temperature SOFCs [84]. Although ceria-based electrolytes exhibit superior ionic conductivity at lower temperatures, they suffer from partial electronic conduction under reducing conditions, decreasing open-circuit voltage and efficiency. Reducing SOFC operating temperature without sacrificing ionic conductivity remains one of the central challenges in electrolyte research.

#### 4.2.4. Composite and Hybrid Electrolytes

Composite and hybrid electrolytes combine multiple phases to simultaneously improve ionic conductivity, mechanical stability, and interfacial compatibility. Polymer–ceramic composite electrolytes are particularly attractive because ceramic fillers can enhance water retention, thermal stability, and proton transport. Similarly, hybrid ceramic–carbonate electrolytes have demonstrated improved low-temperature ionic conductivity in solid oxide systems [85]. However, multiphase systems often suffer from interfacial incompatibility, thermal expansion mismatch, and phase segregation during long-term operation [86]. Therefore, future research should prioritize interface engineering and long-term durability assessment rather than merely maximizing conductivity values.

### 4.3. Electrode and Interconnect Materials

**Figure 6:**
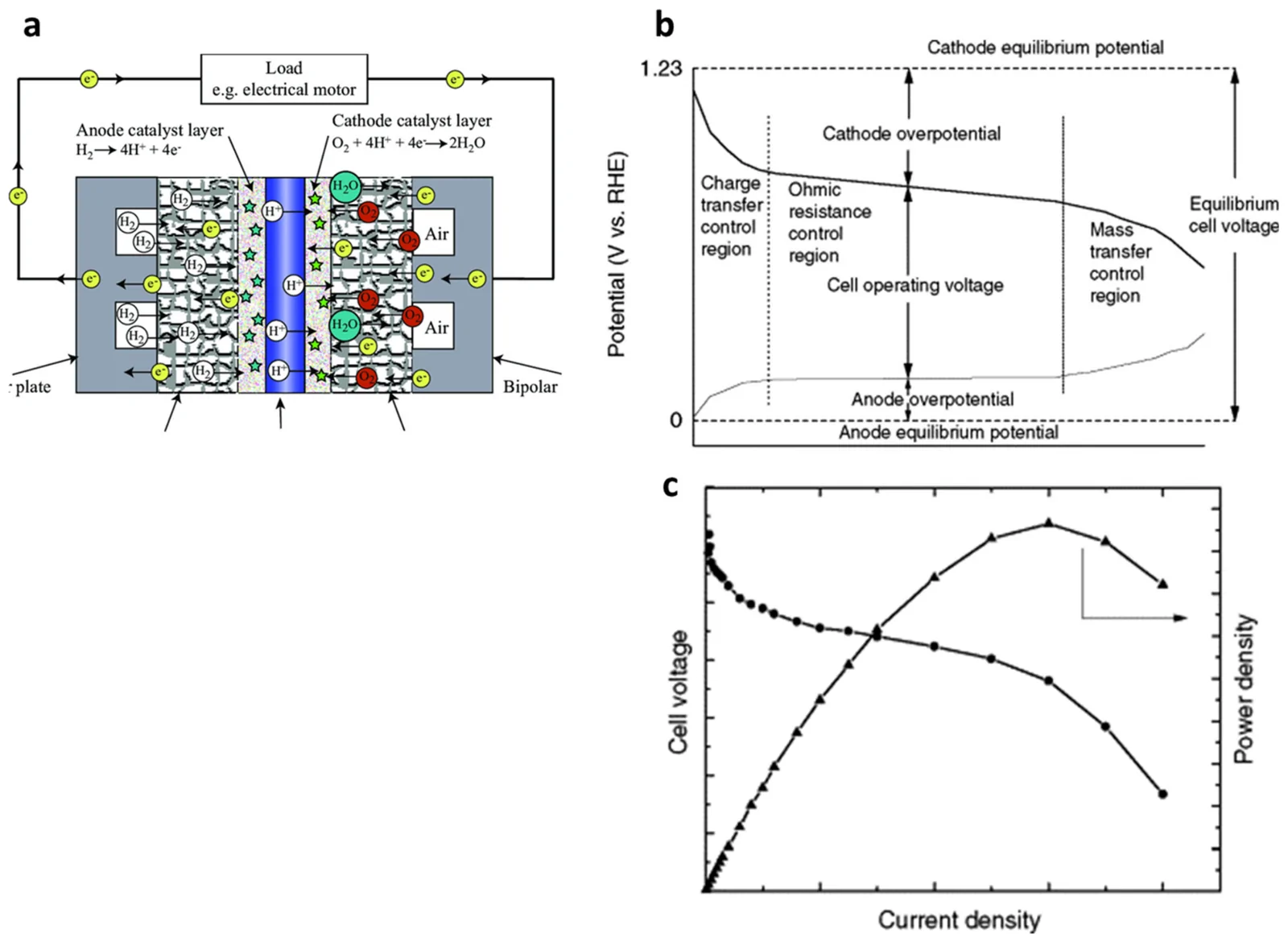
(**a**) Schematic of PEMFC operation. (**b**) Contributions of activation, ohmic, and mass-transfer losses to cell voltage. (**c**) Typical polarization and power-density curves as a function of current density [91].

Electrode materials strongly influence electrochemical activity, gas diffusion, thermal stability, and long-term durability in fuel cells and electrolyzers. Cathode materials are particularly critical because ORR kinetics are inherently sluggish. Perovskite cathodes such as LSM, LSCF, and BSCF exhibit high mixed ionic–electronic conductivity and improved oxygen transport properties. However, cation segregation and chromium poisoning from interconnects frequently degrade cathode performance. Anode materials must simultaneously provide catalytic activity, electrical conductivity, fuel tolerance, and structural stability [87]. Ni-based cermets remain widely used in SOFCs because of excellent conductivity and catalytic activity. Nevertheless, carbon deposition and sulfur poisoning remain major operational challenges [88]. Interconnect materials are equally important because they electrically connect adjacent cells while separating fuel and oxidant gases. Metallic interconnects offer lower cost and better machinability than ceramic interconnects but suffer from oxidation and chromium evaporation. Protective coatings and advanced ferritic stainless steels have therefore become important research areas for improving interconnect durability [89]. The architecture of a typical fuel cell and the functions of its major components are illustrated in Figure 6. The anode, cathode, electrolyte, catalyst layers, gas diffusion layers, and bipolar plates operate synergistically to facilitate fuel oxidation, oxygen reduction, ion transport, electron conduction, and efficient electrochemical energy conversion [90]. Material selection for these components significantly influences catalytic activity, ionic conductivity, durability, corrosion resistance, and overall cell performance.

### 4.4. Nanostructuring and Interface Engineering

**Figure 7:**
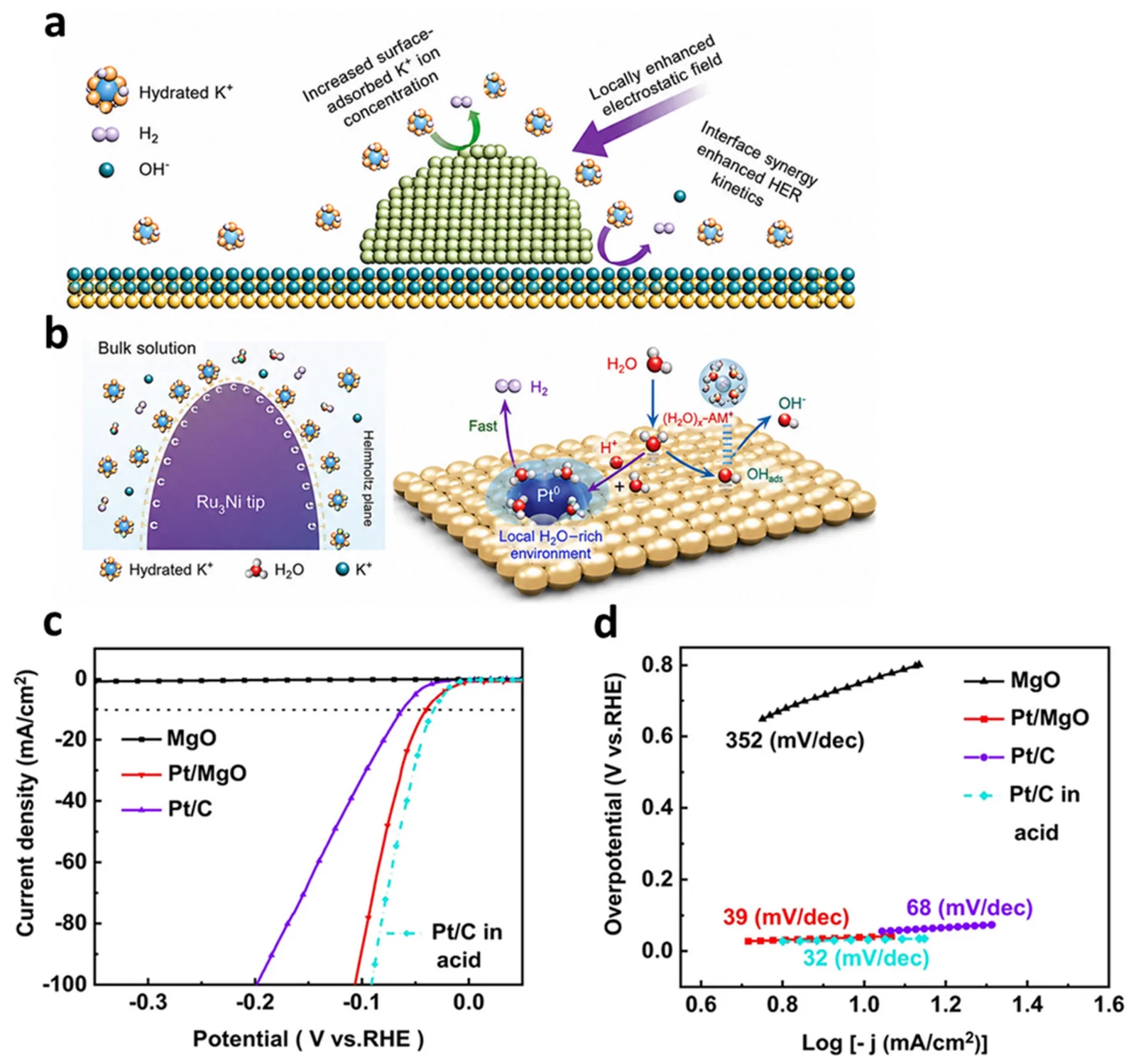
(**a**) Interfacial enhancement of HER kinetics. (**b**) Proposed HER mechanism at the catalyst interface. (**c**) HER polarization curves. (**d**) Corresponding Tafel plots [96]. Minor graphical elements of this figure were prepared with the assistance of OpenAI.

Nanostructuring and interface engineering have become essential strategies for enhancing electrochemical activity, ionic transport, and durability in hydrogen energy systems. Reducing catalyst dimensions to the nanoscale increases active surface area and shortens ion/electron transport pathways. Nanostructured catalysts frequently exhibit enhanced catalytic activity because of quantum confinement effects, altered surface coordination environments, and increased defect concentrations [92]. However, nanostructured materials are often thermodynamically unstable and prone to agglomeration during prolonged operation. Consequently, maintaining nanoscale architectures under industrial conditions remains a major challenge. Heterointerface engineering has emerged as a highly effective approach for improving charge redistribution and adsorption characteristics [93]. Interfaces between dissimilar materials can generate built-in electric fields that facilitate charge transfer and optimize intermediate adsorption energies. Grain-boundary engineering is particularly important in ceramic electrolytes because grain boundaries frequently dominate proton and oxygen-ion transport resistance. Surface modification strategies such as atomic-layer deposition, heteroatom doping, plasma treatment, and protective coatings have also demonstrated substantial improvements in corrosion resistance and electrochemical stability. Importantly, future materials engineering must increasingly transition from empirical trial-and-error approaches toward data-driven rational design assisted by artificial intelligence, machine learning, and high-throughput computational screening. Recent advances in nanostructuring and interface engineering strategies have significantly enhanced the electrochemical performance of hydrogen energy systems [94]. As illustrated in Figure 7, approaches such as nanoparticle design, core–shell architectures, heterostructure formation, grain-boundary engineering, protective coatings, defect engineering, atomic layer deposition (ALD), surface passivation, strong metal–support interactions (SMSI), and heterointerface construction can effectively improve catalytic activity, charge-transfer kinetics, structural stability, and long-term durability while suppressing degradation processes [95].

## 5. Electrochemical Mechanisms and Reaction Pathways

Electrochemical reaction mechanisms fundamentally govern the efficiency, durability, energy conversion capability, and commercialization potential of hydrogen energy and fuel cell technologies. The hydrogen evolution reaction (HER), oxygen reduction reaction (ORR), and oxygen evolution reaction (OER) represent the three most critical electrochemical processes in water electrolysis, fuel cells, and regenerative electrochemical systems [97]. In addition, ionic transport phenomena, including proton conduction and oxygen-ion diffusion, strongly influence the electrochemical performance of proton exchange membrane fuel cells (PEMFCs), solid oxide fuel cells (SOFCs), and proton ceramic fuel cells (PCFCs) [98]. A comprehensive understanding of reaction pathways, surface intermediates, catalyst reconstruction, defect chemistry, and ion transport behavior is therefore essential for the rational design of next-generation electrocatalysts and electrochemical energy devices. Recent developments in operando and in situ characterization techniques have further enabled direct observation of catalytic surfaces, reaction intermediates, oxidation-state evolution, and structural transformations under realistic operating conditions, thereby significantly advancing mechanistic understanding of electrochemical systems [99].

### 5.1. Hydrogen Evolution Reaction (HER)

The hydrogen evolution reaction (HER) is the cathodic half-reaction responsible for hydrogen generation during water electrolysis and plays a central role in alkaline electrolyzers, proton exchange membrane electrolyzers, photoelectrochemical cells, and microbial electrochemical systems [100]. HER kinetics critically determine the energy efficiency and overpotential requirements of electrochemical hydrogen production technologies. Despite the thermodynamically favorable equilibrium potential of hydrogen evolution, sluggish reaction kinetics and large activation barriers often limit practical hydrogen production rates, particularly in alkaline environments. The HER mechanism differs depending on electrolyte conditions, catalyst surface properties, proton availability, and adsorption energetics. In acidic electrolytes, proton reduction dominates the reaction pathway, whereas water dissociation becomes the rate-limiting step in alkaline media because of the relatively low concentration of free protons [101]. Platinum-based catalysts exhibit near-optimal hydrogen adsorption energies and therefore demonstrate the highest HER activity; however, their scarcity and high cost have motivated extensive research into noble-metal-free alternatives including transition-metal phosphides, sulfides, carbides, nitrides, MXenes, and defect-engineered perovskites [102].

Mechanistic Pathways

The HER mechanism generally proceeds through sequential adsorption and desorption steps involving hydrogen intermediates adsorbed on catalytic surfaces (H*). The overall reaction in acidic media is:

2*H*^+^ + 2*e*^−^ → *H*_2_ (46)

In alkaline media, the reaction proceeds via water dissociation:

2*H*_2_*O* + 2*e*^−^ → *H*_2_ + 2*OH*^−^ (47)

The catalytic efficiency strongly depends on the hydrogen adsorption free energy (ΔGH*). According to the Sabatier principle, optimal catalysts should bind hydrogen neither too strongly nor too weakly [103]. If hydrogen adsorption is excessively strong, desorption becomes kinetically hindered; conversely, weak adsorption limits proton reduction efficiency. Volcano plot analyses have demonstrated that Pt lies near the top of the HER activity curve because of its near-zero ΔGH* value. Recent density functional theory (DFT) investigations have revealed that electronic structure engineering, d-band center tuning, heterointerface formation, and vacancy engineering can significantly optimize hydrogen adsorption behavior. For instance, heterostructured Ni_2_P/MoS_2_ interfaces have demonstrated enhanced HER activity because of synergistic charge redistribution and accelerated water dissociation kinetics [104]. Similarly, defect-rich MXenes and single-atom catalysts exhibit improved HER activity due to unsaturated coordination environments and increased active-site density.

Volmer–Heyrovsky–Tafel Mechanisms

HER typically proceeds through combinations of three elementary reaction steps known as the Volmer, Heyrovsky, and Tafel mechanisms.

Volmer Step (Electrochemical Hydrogen Adsorption)

In acidic media:

*H*^+^ + *e*^−^ + ∗ → H* (48)

In alkaline media:

*H*_2_*O* + *e*^−^ + ∗ → H* + *OH*^−^ (49)

The Volmer step involves proton or water reduction and adsorption of atomic hydrogen on the catalyst surface [105]. In alkaline systems, water dissociation introduces additional activation barriers, making HER significantly slower than in acidic media.

Heyrovsky Step (Electrochemical Desorption)

H* + *H*^+^ + *e*^−^ → *H*_2_ + ∗ (50)

or in alkaline media:

H* + *H*_2_*O* + *e*^−^ → *H*_2_ + *OH*^−^ + ∗ (51)

Tafel Step (Chemical Recombination)

2H* → *H*_2_ + 2 ∗ (52)

Depending on the dominant rate-limiting step, HER may follow either the Volmer–Heyrovsky or Volmer–Tafel pathway [106]. Tafel slope analysis is widely employed to determine the governing reaction mechanism. Typical Tafel slopes include approximately 120 mV dec^−1^ for Volmer-limited kinetics, 40 mV dec^−1^ for Heyrovsky-limited kinetics, and 30 mV dec^−1^ for Tafel-controlled hydrogen recombination [107].

Recent operando spectroscopic studies have shown that dynamic surface reconstruction, electrolyte interactions, local pH gradients, and adsorbate-induced electronic perturbations significantly influence HER pathways under realistic operating conditions [108]. Advanced catalysts with hierarchical porosity, strain engineering, and conductive heterointerfaces have therefore become important strategies for improving HER kinetics and reducing overpotentials.

**Figure 8:**
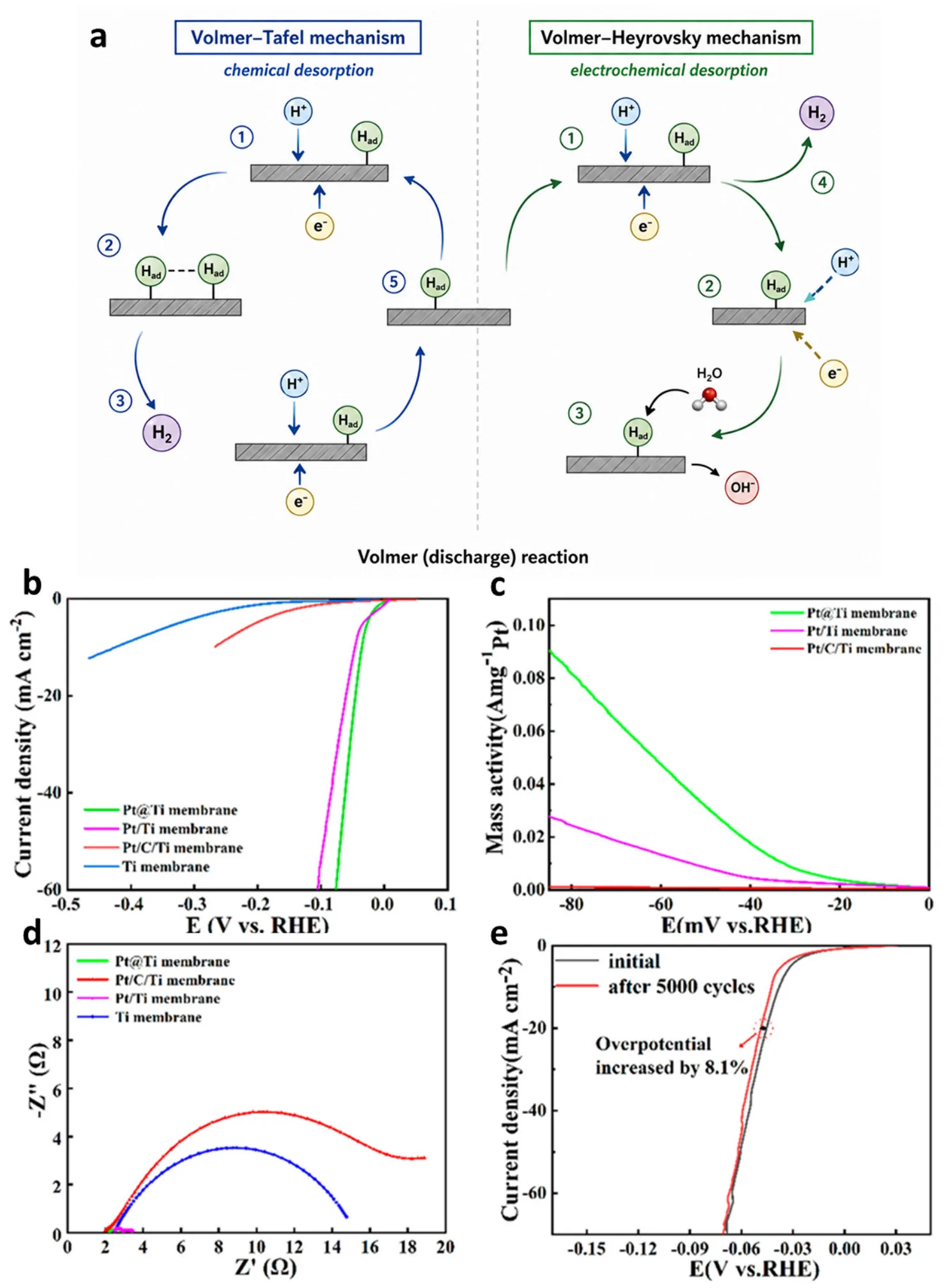
(**a**) Hydrogen Evolution Reaction (HER) Mechanisms. (**b**) HER Polarization Curves (LSV). (**c**) Mass Activity Evaluation. (**d**) Electrochemical Impedance Spectroscopy (EIS) Analysis. (**e**) Durability and Cycling Stability [112,113].

Several studies have demonstrated that transition-metal phosphides such as CoP and Ni_2_P exhibit HER activities approaching those of Pt due to favorable hydrogen adsorption energies and efficient charge transfer [109]. Similarly, MoS_2_ edge sites have been identified as highly active HER centers, whereas basal planes are comparatively inert, motivating extensive defect-engineering approaches to expose additional catalytic edge sites. Single-atom catalysts anchored on conductive carbon supports have also emerged as promising HER catalysts because of their maximized atomic utilization efficiency and tunable electronic coordination environments [110]. The fundamental HER pathways and associated reaction intermediates are illustrated in Figure 8. The reaction proceeds through hydrogen adsorption on the catalyst surface (Volmer step), followed by either electrochemical desorption (Heyrovsky step) or chemical recombination (Tafel step). The dominant pathway depends on catalyst properties, electrolyte conditions, and reaction kinetics, which collectively determine hydrogen evolution activity and overpotential requirements [111].

### 5.2. Oxygen Reduction Reaction (ORR)

The oxygen reduction reaction (ORR) is one of the most kinetically sluggish electrochemical reactions in fuel cells and metal–air batteries. ORR occurs at the cathode and largely determines the voltage efficiency, power density, and long-term performance of PEMFCs, alkaline fuel cells, and Zn–air batteries [114]. Despite extensive catalyst development efforts, ORR remains associated with substantial activation losses because of its complex multi-electron transfer pathways and strong oxygen–surface interactions. The ORR process may proceed through either a direct four-electron pathway or a two-electron peroxide pathway, depending on catalyst selectivity and electrolyte conditions.

Two-Electron vs. Four-Electron Pathways

The desired four-electron ORR pathway in acidic media is:

*O*_2_ + 4*H*^+^ + 4*e*^−^ → 2*H*_2_*O* (53)

In alkaline media:

*O*_2_ + 2*H*_2_*O* + 4*e*^−^ → 4*OH*^−^ (54)

The alternative two-electron pathway generates peroxide intermediates:

*O*_2_ + 2*H*^+^ + 2*e*^−^ → *H*_2_*O*_2_ (55)

**Figure 9:**
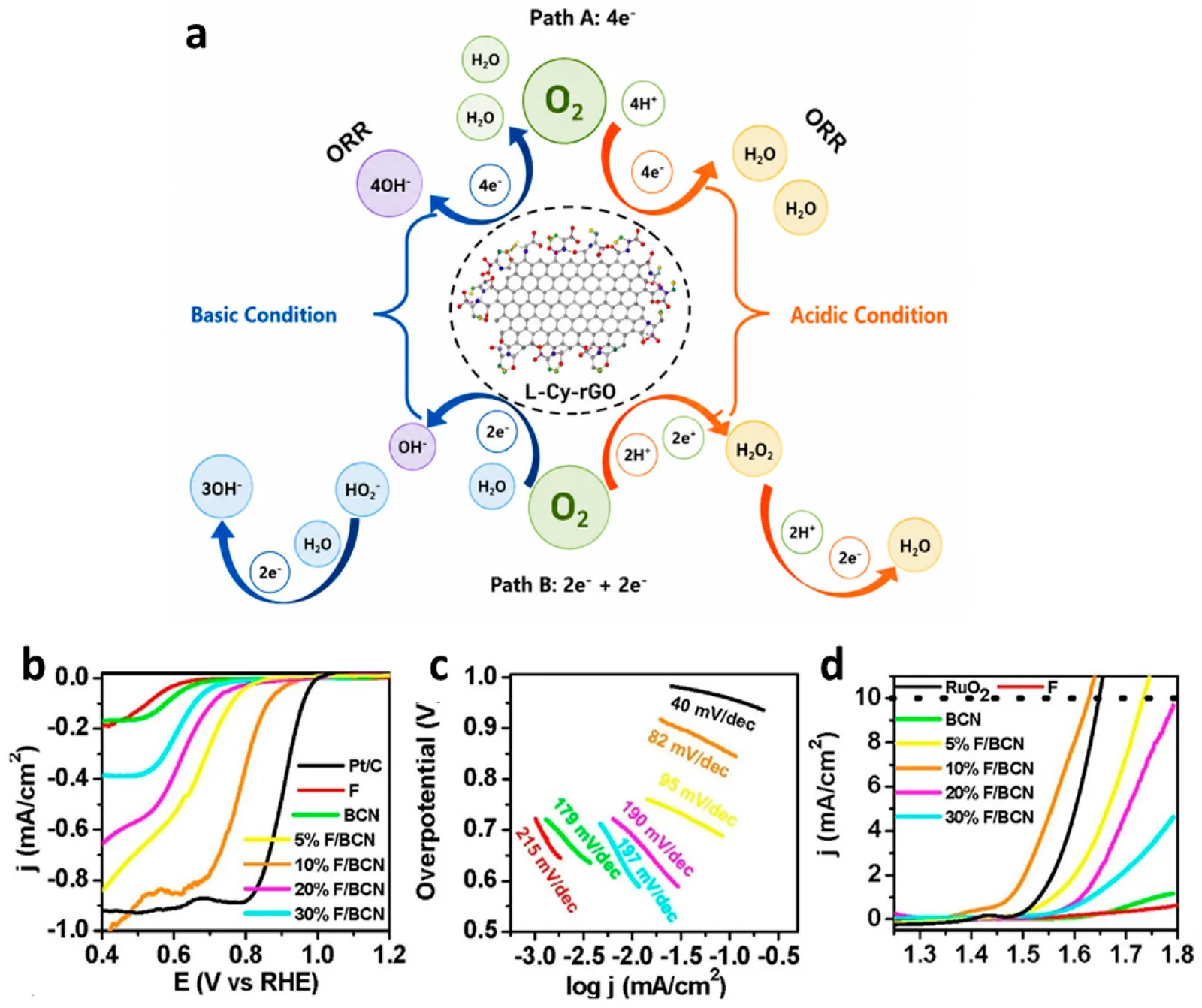
(**a**) Proposed ORR Mechanism under Acidic and Alkaline Conditions. (**b**) Linear Sweep Voltammetry (LSV) Curves. (**c**) Tafel Slope Analysis. (**d**) Oxygen Reduction Reaction (ORR) Polarization Curves [120].

Hydrogen peroxide formation is generally undesirable because peroxide species can attack polymer electrolytes, accelerate membrane degradation, corrode catalysts, and reduce fuel cell durability. Therefore, highly selective four-electron ORR catalysts are essential for practical electrochemical systems. The ORR mechanism typically involves oxygen adsorption, O-O bond activation, proton/electron transfer, and desorption of hydroxyl or water intermediates. The adsorption strength of oxygen-containing intermediates such as O*, OH*, and OOH* critically determines catalytic activity [115]. The oxygen reduction reaction can proceed through either a direct four-electron pathway or an indirect two-electron pathway, depending on catalyst selectivity and reaction conditions. As illustrated in Figure 9, the four-electron pathway directly converts oxygen to water (or hydroxide ions in alkaline media) and is highly desirable because of its superior energy efficiency and durability [116]. In contrast, the two-electron pathway produces peroxide intermediates that can accelerate catalyst degradation and reduce fuel cell performance.

Catalyst Activity and Selectivity

Pt-based catalysts remain the benchmark ORR catalysts because of their favorable oxygen-binding characteristics and excellent catalytic kinetics. However, Pt suffers from high cost, dissolution, Ostwald ripening, carbon-support corrosion, and susceptibility to poisoning, which severely limit large-scale commercialization.

Consequently, substantial efforts have focused on developing Pt-alloy catalysts, atomically dispersed catalysts, heteroatom-doped carbons, and transition-metal–nitrogen–carbon (M-N-C) catalysts. Fe-N-C catalysts have emerged as particularly promising ORR materials because of their high intrinsic activity and reduced precious-metal dependence [117]. Advanced spectroscopic studies have identified Fe–N_4_ coordination centers as major active ORR sites. Electronic structure engineering significantly affects ORR performance. Alloying Pt with transition metals such as Co, Ni, and Fe can downshift the Pt d-band center, thereby weakening oxygen adsorption and improving ORR kinetics. Core–shell nanostructures, strain engineering, and surface defect modulation have also demonstrated substantial improvements in catalytic activity and durability. The ORR process is additionally influenced by electrolyte pH, catalyst morphology, mass transport behavior, and interfacial water structure. Rotating disk electrode (RDE) studies combined with Koutecky–Levich analysis are commonly employed to determine electron transfer numbers and reaction kinetics [118]. Recent advances in operando X-ray absorption spectroscopy (XAS) and ambient-pressure XPS have revealed dynamic oxidation-state fluctuations and adsorbate-induced surface restructuring during ORR operation. These findings suggest that catalyst surfaces are highly dynamic rather than static under realistic electrochemical conditions. Furthermore, heterointerface engineering has emerged as an effective strategy for improving ORR activity [119]. Interfaces between transition metals and conductive carbons facilitate charge redistribution and modify oxygen adsorption behavior. Similarly, oxygen-vacancy-rich perovskites and spinel oxides exhibit enhanced ORR kinetics because of improved oxygen mobility and electronic conductivity.

### 5.3. Oxygen Evolution Reaction (OER)

The oxygen evolution reaction (OER) is the anodic half-reaction in water electrolysis and regenerative electrochemical systems. OER is generally considered the major kinetic bottleneck in electrolyzers because it involves a complex four-electron transfer process with multiple adsorbed oxygen intermediates.

The overall OER reaction in acidic media is:

2*H*_2_*O* → *O*_2_ + 4*H*^+^ + 4*e*^−^ (56)

4*OH*^−^ → *O*_2_ + 2*H*_2_*O* + 4*e*^−^ (57)

OER requires substantial overpotential because of sluggish reaction kinetics and high activation barriers associated with O-O bond formation. IrO_2_ and RuO_2_ remain the most active OER catalysts under acidic conditions; however, their scarcity and instability limit widespread commercialization.

Surface Reconstruction Mechanisms

Recent studies have revealed that many OER catalysts undergo dynamic surface reconstruction during operation. Transition-metal oxides, hydroxides, phosphides, sulfides, and nitrides frequently transform into oxyhydroxide-rich surfaces under anodic potentials, and these reconstructed phases often represent the actual active catalytic species [121].

For example, NiFe layered double hydroxides (LDHs) undergo oxidation into high-valence NiOOH structures during OER operation. Similarly, cobalt phosphides and sulfides reconstruct into CoOOH-like surfaces under alkaline anodic conditions [122]. Operando Raman spectroscopy and XAS studies have demonstrated that catalyst surfaces dynamically evolve during electrochemical operation rather than remaining chemically static. Lattice oxygen participation mechanisms (LOM) have also attracted significant attention [123]. In conventional adsorbate evolution mechanisms (AEM), oxygen evolves solely from adsorbed intermediates. In contrast, LOM involves direct participation of lattice oxygen atoms in O_2_ formation, which can reduce energy barriers and improve catalytic activity [124]. However, excessive lattice oxygen participation may also accelerate catalyst degradation through oxygen-vacancy accumulation and structural destabilization.

Stability and Degradation

Although many transition-metal catalysts exhibit excellent initial OER activity, long-term stability remains a major challenge. Catalyst degradation mechanisms include dissolution, phase segregation, surface amorphization, metal leaching, carbon corrosion, and oxygen-vacancy-induced structural collapse. Under acidic conditions, catalyst stability becomes particularly problematic because highly oxidizing anodic environments accelerate metal dissolution. Ru-based catalysts exhibit excellent activity but often suffer from severe instability due to Ru dissolution at high potentials. Ir-based catalysts demonstrate superior stability but remain prohibitively expensive [125]. Several strategies have been developed to improve OER durability, including:

- Heterostructure engineering
- Surface passivation
- Doping with stabilizing cations
- Core–shell architectures
- Oxygen-vacancy optimization
- Conductive support integration

Advanced computational studies combined with operando spectroscopy have further enabled atomic-level understanding of catalyst degradation pathways. These investigations have revealed that dynamic electronic structure evolution, surface hydroxylation, and local coordination changes strongly influence long-term catalyst durability. Perovskite oxides such as Ba_0.5_Sr_0.5_Co_0.8_Fe_0.2_O_3−δ_ and layered double hydroxides have shown particularly promising OER activity because of their tunable electronic structures, oxygen-vacancy mobility, and adaptable surface chemistry [126]. Similarly, high-entropy oxides have recently emerged as promising OER materials because of entropy-stabilized crystal structures and compositional flexibility.

### 5.4. Proton and Oxygen Ion Transport Mechanisms

Ion transport mechanisms critically determine the electrochemical efficiency of fuel cells, electrolyzers, and solid-state electrochemical devices. Proton and oxygen-ion conduction strongly influence polarization resistance, operating temperature, fuel utilization, and power density.

Bulk Diffusion

Bulk ionic diffusion occurs through crystal lattices via defect-mediated hopping mechanisms. In proton-conducting ceramics, proton conduction typically occurs through hydration and proton hopping processes. For proton-conducting perovskites:

*H*_2_*O* + *VO*^∙∙^ + *OO*^x^ → 2*OHO*^∙^ (58)

where oxygen vacancies incorporate water molecules to generate mobile protonic defects.

Proton transport generally proceeds through the Grotthuss mechanism involving rotational reorientation and proton hopping between neighboring oxygen ions. Materials such as BaZrO_3_, BaCeO_3_, and Ba(Zr,Ce)O_3_-based perovskites exhibit high proton conductivity because of favorable oxygen-vacancy formation energies and flexible lattice structures. In oxygen-ion-conducting SOFC electrolytes such as yttria-stabilized zirconia (YSZ), ionic transport occurs through oxygen-vacancy diffusion [127]:

*O*_O_^x^ + *VO*^∙∙^ → *VO*^∙∙^ + *O*_O_^x^ (59)

High oxygen-vacancy concentration and low migration barriers are therefore essential for efficient oxygen-ion conductivity.

Grain Boundary Conduction

Grain boundaries strongly influence ionic transport in ceramic electrolytes. In many proton-conducting materials, grain boundaries exhibit higher resistance than grain interiors because of impurity segregation, space-charge effects, and defect accumulation. In nanocrystalline electrolytes, grain-boundary density increases substantially, making interfacial conduction particularly important. Dopant segregation and secondary phase formation at grain boundaries may block ionic transport and reduce total conductivity [128]. Conversely, engineered grain boundaries can sometimes enhance ionic conductivity by facilitating defect accumulation or reducing activation barriers. Advanced sintering strategies, A-site deficiency engineering, and grain-boundary modification approaches have therefore become important for optimizing electrochemical performance in PCFC and SOFC electrolytes.

Defect Chemistry

Defect chemistry fundamentally governs ionic conductivity, catalytic activity, and electrochemical stability in energy materials. Oxygen vacancies, protonic defects, cation non-stoichiometry, and electronic defects strongly influence charge transport and reaction kinetics. Acceptor doping is widely employed to generate oxygen vacancies and enhance ionic conductivity. For example, Y^3+^ substitution into Zr^+4^ sites in YSZ creates oxygen vacancies that facilitate oxygen-ion transport [129]. Similarly, Y-doped BaZrO_3_ and Gd-doped ceria exhibit improved proton or oxygen-ion conductivity because of optimized defect concentrations. However, excessive defect concentrations may also promote electronic leakage, chemical instability, and phase decomposition. Consequently, defect engineering requires careful optimization between conductivity enhancement and structural stability. Recent computational investigations using density functional theory and molecular dynamics simulations have significantly improved understanding of defect migration pathways, hydration thermodynamics, and ion transport energetics in proton-conducting ceramics and oxide electrolytes.

### 5.5. Operando and In Situ Characterization

The development of advanced operando and in situ characterization techniques has revolutionized mechanistic understanding of electrochemical energy materials. Unlike ex situ methods, operando techniques enable direct observation of catalysts under realistic electrochemical operating conditions, thereby capturing transient intermediate states, dynamic reconstruction processes, and reaction-induced structural evolution.

XPS, XRD, and Raman Spectroscopy

Operando X-ray photoelectron spectroscopy (XPS) provides valuable insights into oxidation-state evolution, surface composition changes, adsorbate interactions, and electronic structure modifications during electrochemical reactions. Ambient-pressure XPS has become particularly important for investigating gas–solid interfaces under realistic catalytic environments [130]. Operando X-ray diffraction (XRD) enables real-time monitoring of phase transitions, lattice expansion, crystallographic evolution, and structural reconstruction during electrochemical cycling. Several studies have demonstrated that transition-metal catalysts dynamically transform into oxyhydroxide phases during OER operation, which are often undetectable under ex situ conditions [130]. Operando Raman spectroscopy is highly sensitive to surface vibrational modes and has therefore become a powerful technique for identifying reaction intermediates, oxygen-vacancy evolution, and surface hydroxylation processes. Raman investigations have revealed dynamic formation of NiOOH and CoOOH species during alkaline OER [131].

Neutron Diffraction

Neutron diffraction provides unique advantages for investigating hydrogen-containing materials because neutrons interact strongly with light elements such as hydrogen and oxygen. This technique has therefore become critically important for studying proton positions, hydration behavior, oxygen-vacancy distributions, and ionic transport pathways in proton-conducting ceramics. Neutron scattering studies have significantly advanced understanding of proton migration mechanisms in BaZrO_3_-based electrolytes and hydration-induced lattice distortions in PCFC materials.

TEM and Synchrotron Analysis

Advanced transmission electron microscopy (TEM) techniques including environmental TEM (ETEM), high-angle annular dark-field scanning TEM (HAADF-STEM), and electron energy-loss spectroscopy (EELS) enable atomic-scale visualization of catalyst structures, defect distributions, and interface evolution [132]. In situ TEM investigations have directly observed nanoparticle coarsening, catalyst dissolution, phase reconstruction, and oxygen-vacancy migration under electrochemical conditions. Similarly, synchrotron-based techniques such as X-ray absorption spectroscopy (XAS), near-edge X-ray absorption fine structure (NEXAFS), and pair distribution function (PDF) analysis provide detailed electronic and local structural information with exceptional temporal and spatial resolution [133]. Recent synchrotron investigations have revealed that many electrocatalysts exhibit highly dynamic electronic structures during operation, challenging conventional assumptions regarding static active sites. These findings are reshaping catalyst design strategies toward adaptive and dynamically reconfigurable catalytic systems. Overall, operando and in situ characterization techniques have become indispensable tools for understanding electrochemical reaction mechanisms, catalyst evolution, ionic transport pathways, and degradation phenomena in hydrogen energy technologies [134]. Their integration with computational modeling, machine learning, and multiscale simulations is expected to significantly accelerate the discovery of next-generation electrocatalysts and electrochemical materials for sustainable hydrogen energy systems.

## 6. Performance, Durability, and Degradation Mechanisms

The practical commercialization and long-term deployment of fuel cell technologies strongly depend on achieving high electrochemical performance together with exceptional operational durability and degradation resistance. Although remarkable advancements have been achieved in electrocatalyst engineering, electrolyte development, electrode architecture optimization, and interface tailoring, long-term stability remains one of the most critical barriers limiting the widespread adoption of hydrogen-based electrochemical systems. Fuel cells and electrolyzers continuously operate under highly aggressive physicochemical environments involving elevated temperatures, humidified atmospheres, redox cycling, electrochemical polarization, mechanical stress, fuel impurities, and thermal gradients [135]. These harsh operating conditions progressively deteriorate catalyst activity, electrolyte conductivity, interfacial integrity, and structural stability, ultimately leading to performance decay and shortened device lifetime. Consequently, understanding electrochemical performance characteristics and degradation mechanisms has become fundamentally important for the rational design of durable next-generation hydrogen energy systems [136]. Performance degradation in electrochemical devices is highly complex and multiscale in nature, involving coupled electrochemical, chemical, thermal, and mechanical processes. At the atomic and nanoscale, catalyst dissolution, nanoparticle agglomeration, carbon corrosion, ionic defect redistribution, and grain-boundary segregation contribute to the gradual loss of electrochemical activity. Simultaneously, macroscopic degradation phenomena such as thermal stress, electrode delamination, electrolyte cracking, and sealing failure further accelerate system deterioration. Recent advances in operando characterization techniques, accelerated stress testing protocols, multiscale computational modeling, and machine-learning-assisted degradation prediction have significantly improved the mechanistic understanding of these failure pathways [137]. In parallel, emerging strategies involving protective coatings, defect engineering, interface stabilization, nanostructured architectures, and self-healing materials are increasingly being explored to improve electrochemical durability and long-term operational stability.

### 6.1. Fuel Cell Performance Parameters

**Figure 10:**
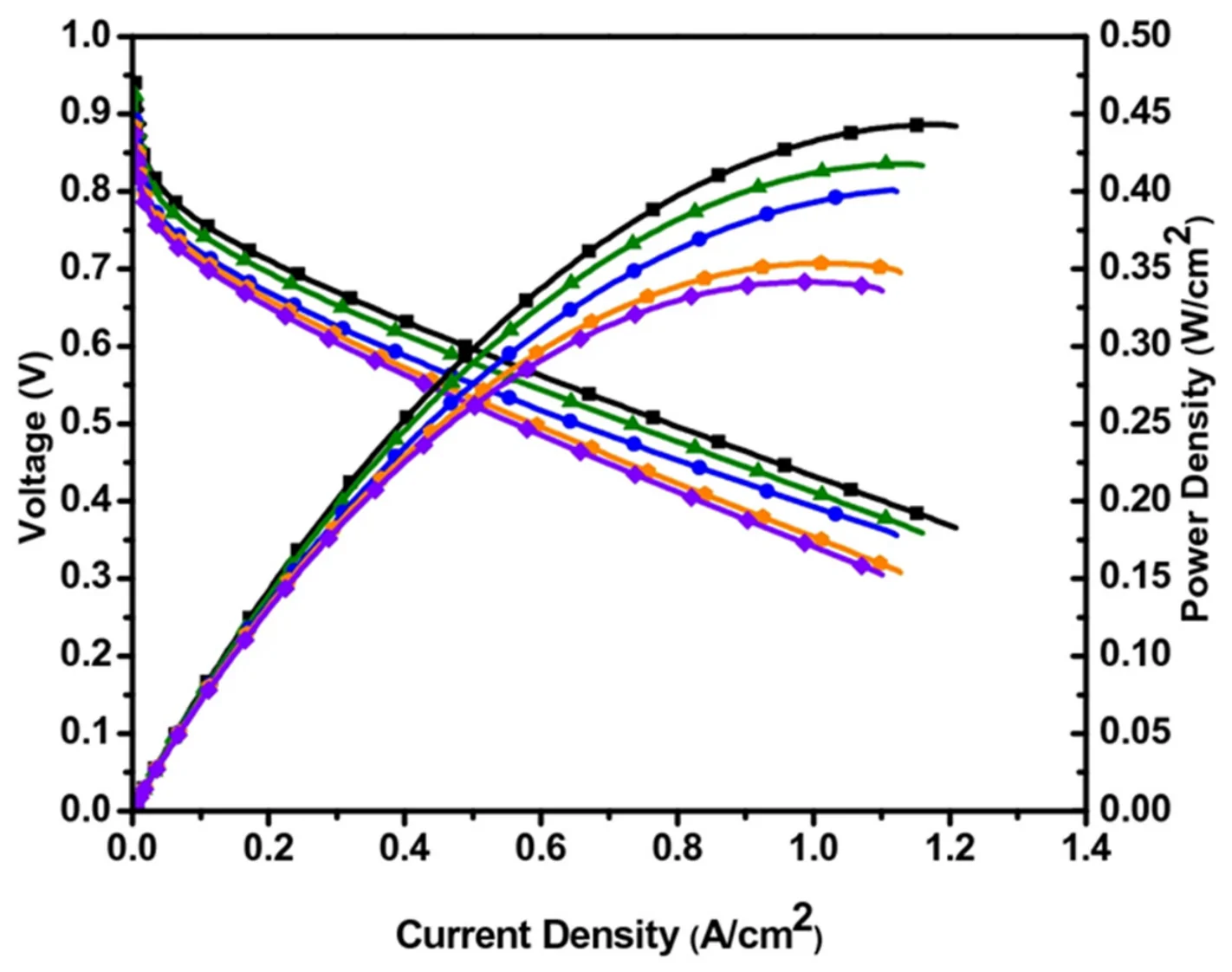
IVP curve of fuel cells under different operating conditions [145,146].

The electrochemical performance of fuel cells is commonly evaluated using several important parameters, including power density, efficiency, polarization characteristics, and electrochemical impedance behavior. These parameters provide critical insights into electrochemical kinetics, ionic conductivity, charge-transfer behavior, mass transport phenomena, and overall energy conversion capability [138]. The principal electrochemical performance parameters used to evaluate fuel cell operation, including polarization behavior, power density, efficiency, impedance characteristics, and degradation trends, are schematically illustrated in Figure 10.

Power Density

Power density is one of the most important performance indicators because it determines the amount of electrical power generated per unit electrode area or cell volume. High power density is especially critical for transportation systems, portable electronic devices, aerospace applications, and compact energy conversion technologies where space and weight limitations are significant considerations [139]. The power output of a fuel cell is governed by the relationship between current density and cell voltage:

*P* = *IVP* (60)

where *P* represents power density, *I* denotes current density, and *V* corresponds to cell voltage.

In practical fuel cell systems, voltage gradually decreases with increasing current density because of activation losses, ohmic resistance, and concentration polarization. Therefore, maximizing power density requires simultaneous optimization of catalytic activity, ionic conductivity, gas diffusion, water management, and electrode microstructure. In PEMFCs, high power densities exceeding 1 W cm^−2^ have been achieved through the use of Pt-alloy nanocatalysts, ultrathin proton-conducting membranes, and highly optimized gas diffusion layers [140]. Similarly, recent advances in SOFCs and PCFCs involving nanostructured electrodes, mixed ionic–electronic conductors, and engineered triple-phase boundaries have substantially improved electrochemical performance at intermediate operating temperatures. Electrode microstructure also plays a critical role in determining achievable power density. Hierarchically porous electrode architectures facilitate efficient gas diffusion, electron transport, and ionic conduction while simultaneously maximizing electrochemically active surface area [141]. Consequently, advanced microstructural engineering strategies are increasingly employed to minimize transport limitations and improve electrochemical utilization efficiency.

Efficiency

Fuel cell efficiency describes the ability of the electrochemical system to convert chemical energy directly into electrical energy. Unlike conventional combustion-based systems, fuel cells bypass thermodynamic Carnot-cycle limitations because electricity is produced through direct electrochemical reactions rather than thermal conversion processes [142]. This direct conversion mechanism enables significantly higher energy efficiencies and lower environmental impact. The theoretical efficiency of a hydrogen fuel cell can be expressed using the Gibbs free-energy relationship:

(61) $$\eta =\frac{\Delta G}{\Delta H}$$

where ΔG represents the Gibbs free-energy change and ΔH corresponds to the total enthalpy change of the reaction.

The practical operating efficiency of fuel cells is often expressed as:

(62) $$\eta =\frac{\mathrm{Vcell}}{\mathrm{Vthermoneutral}}$$

PEMFC systems typically exhibit electrical efficiencies between 40% and 60%, whereas SOFCs can exceed 65% electrical efficiency because of improved reaction kinetics and efficient utilization of high-temperature waste heat. When integrated into combined heat and power (CHP) systems, total energy efficiencies may surpass 80% [143]. Several factors influence overall fuel cell efficiency, including activation overpotential, ionic resistance, gas crossover, thermal losses, water flooding, fuel utilization, and catalyst degradation. Under high-current-density operation, efficiency generally decreases because polarization losses increase substantially. Consequently, balancing high power output with long-term energy efficiency remains a major challenge in practical fuel cell system design.

Polarization Curves

Polarization curves are among the most important electrochemical diagnostic tools used for evaluating fuel cell performance. These curves describe the relationship between cell voltage and current density and provide valuable insights into various electrochemical loss mechanisms occurring during operation.

The practical fuel cell voltage can be represented as:

*V* = *E* − *η*_act_ − *η*_ohmic_ − *η*_conc_ (63)

where *E* represents equilibrium voltage, *η_act_* corresponds to activation polarization, *η_ohmic_* denotes ohmic losses, and *η_conc_* represents concentration polarization.

At low current densities, activation polarization dominates because of sluggish electrochemical reaction kinetics, particularly the oxygen reduction reaction occurring at the cathode. At intermediate current densities, ohmic losses associated with ionic and electronic transport become significant. At high current densities, concentration polarization becomes dominant because reactant diffusion cannot adequately sustain electrochemical reaction rates. Polarization analysis is extensively employed to optimize catalyst composition, electrode microstructure, operating conditions, and gas transport properties. Furthermore, long-term shifts in polarization behavior are commonly used to evaluate degradation rates and operational durability.

Electrochemical Impedance Spectroscopy

Electrochemical impedance spectroscopy (EIS) has become one of the most powerful techniques for investigating electrochemical processes in fuel cells and electrolyzers. EIS enables separation of different resistance contributions associated with ionic conduction, charge transfer, gas diffusion, and interfacial polarization.

The impedance response is expressed as:

*Z*(*ω*) = *Z*′ + *jZ*″ (64)

where *Z*′ and *Z*″ represent the real and imaginary impedance components, respectively.

Nyquist and Bode plots generated from impedance measurements provide detailed information regarding electrolyte resistance, grain-boundary conductivity, electrode polarization, and mass transport limitations. High-frequency impedance arcs are generally associated with electrolyte and contact resistance, whereas low-frequency arcs correspond to gas diffusion and electrode kinetics [144]. In SOFCs and PCFCs, EIS is particularly valuable for distinguishing bulk ionic conductivity from grain-boundary resistance. In PEMFCs, impedance analysis is widely used to investigate membrane hydration, water flooding, catalyst degradation, and electrode aging. Recent developments involving distribution of relaxation time (DRT) analysis and machine-learning-assisted impedance interpretation have further improved mechanistic understanding of electrochemical processes and degradation phenomena [145].

### 6.2. Degradation Mechanisms

Electrochemical degradation in fuel cells involves multiple coupled physicochemical processes that progressively reduce catalytic activity, ionic conductivity, structural integrity, and overall electrochemical performance. Degradation pathways strongly depend on operating temperature, electrochemical potential, humidity, fuel composition, thermal cycling, and current load conditions. Because several degradation mechanisms often occur simultaneously, long-term operational stability remains one of the most critical challenges limiting commercial fuel cell deployment.

#### 6.2.1. Catalyst Degradation

Catalyst degradation is among the most severe causes of fuel cell performance loss, particularly in PEMFC systems employing Pt-based electrocatalysts. Under prolonged electrochemical operation, catalyst nanoparticles experience dissolution, agglomeration, Ostwald ripening, surface oxidation, and poisoning, all of which reduce electrochemically active surface area and catalytic efficiency [147]. Repeated potential cycling accelerates Pt dissolution and subsequent redeposition onto larger particles, causing significant nanoparticle coarsening. Simultaneously, particle migration and agglomeration decrease the density of active catalytic sites available for electrochemical reactions. Alloy catalysts additionally suffer from selective transition-metal leaching under acidic operating conditions, resulting in compositional instability and structural degradation. Catalyst poisoning by impurities such as CO, sulfur-containing compounds, ammonia, and chloride species further suppresses catalytic activity by blocking active sites and altering surface adsorption behavior. In SOFCs and PCFCs, catalyst degradation often involves redox instability, phase segregation, cation migration, and coarsening of Ni-based electrodes [148]. Repeated redox cycling can induce severe microstructural damage because of volumetric expansion and contraction associated with Ni oxidation and reduction processes [149]. Recent operando TEM and synchrotron studies have demonstrated that catalyst surfaces undergo continuous dynamic reconstruction during electrochemical operation. Consequently, understanding transient catalyst evolution has become critically important for designing highly durable electrocatalytic systems.

#### 6.2.2. Carbon Corrosion

Carbon corrosion represents a major degradation mechanism in PEMFC catalyst supports and gas diffusion layers because conventional carbon materials are thermodynamically unstable under highly oxidizing electrochemical conditions [150]. Carbon oxidation becomes particularly severe during startup–shutdown cycles, fuel starvation events, and high-potential excursions. The carbon corrosion reaction is expressed as:

*C* + 2*H*_2_*O* → *CO*_2_ + 4*H*^+^ + 4*e*^−^ (65)

Carbon corrosion leads to catalyst detachment, conductivity loss, electrode thinning, pore collapse, and severe mass transport limitations. High operating potentials above approximately 1.2 V significantly accelerate carbon oxidation kinetics [151]. Furthermore, oxygen crossover and localized hydrogen starvation create highly corrosive electrochemical environments that intensify structural degradation. To address these issues, researchers are increasingly developing graphitized carbons, graphene-based supports, carbon nanotubes, conductive ceramics, MXenes, and corrosion-resistant oxide supports. Although conductive oxide supports provide improved chemical stability, their relatively low electrical conductivity remains a challenge requiring further optimization [152].

#### 6.2.3. Electrolyte Degradation

Electrolyte degradation critically affects ionic conductivity, gas separation capability, and electrochemical efficiency. In PEMFCs, membrane degradation is primarily caused by radical-induced polymer decomposition, dehydration, mechanical fatigue, and chemical attack from peroxide-derived reactive oxygen species. Hydroxyl radicals generated during peroxide decomposition attack polymer backbones and sulfonic acid groups, leading to membrane thinning, pinhole formation, and proton conductivity loss. Repeated hydration–dehydration cycles further induce mechanical stress and structural damage [153]. In SOFCs and PCFCs, electrolyte degradation commonly involves grain-boundary impurity segregation, phase instability, interfacial reactions, and thermal mismatch. For example, YSZ electrolytes may react with cathode materials to form resistive interfacial compounds, whereas Ba-based proton-conducting electrolytes may undergo carbonation or hydroxylation under CO^−2^ and H_2_O-containing atmospheres. These degradation processes reduce ionic conductivity and accelerate electrochemical failure.

#### 6.2.4. Thermal and Mechanical Instability

Thermal and mechanical degradation significantly influence the long-term stability of fuel cells operating under elevated temperatures and dynamic thermal conditions. Repeated thermal cycling generates severe stresses because different fuel cell components often possess dissimilar thermal expansion coefficients [154]. These stresses can induce crack formation, interfacial delamination, seal failure, and electrode spallation. SOFCs are particularly vulnerable because ceramic materials exhibit brittle mechanical behavior under thermal stress. Rapid startup and shutdown cycles create large temperature gradients that may lead to catastrophic structural failure. In PEMFCs, repeated membrane swelling and contraction during hydration cycles cause mechanical fatigue, crack propagation, and gas crossover. Recent multiscale modeling investigations have demonstrated that coupled thermo-electrochemical–mechanical interactions strongly govern long-term degradation behavior. Consequently, advanced thermal management strategies and mechanically robust materials are critically important for improving operational reliability [155].

### 6.3. Strategies for Enhancing Durability

Improving fuel cell durability requires integrated approaches involving catalyst stabilization, interface engineering, defect optimization, thermal management, and advanced protective architectures. Considerable research efforts are currently focused on suppressing degradation pathways while maintaining high electrochemical performance under realistic operating conditions. Protective coatings have emerged as highly effective approaches for suppressing catalyst dissolution, corrosion, and chemical instability [56]. Thin oxide coatings deposited using techniques such as atomic layer deposition can significantly enhance chemical durability while preserving catalytic activity. Materials including TiO_2_, CeO_2_, ZrO_2_, and Al_2_O_3_ are frequently employed to improve corrosion resistance and thermal stability [156]. In SOFC systems, spinel protective coatings are extensively used to suppress chromium evaporation and minimize cathode poisoning.

Material optimization strategies are also critically important for improving intrinsic electrochemical durability. Advanced doping engineering, defect chemistry control, alloy formation, nanostructuring, and high-entropy material design have demonstrated substantial improvements in catalytic stability and structural robustness [157]. For instance, Pt-alloy catalysts with optimized electronic structures exhibit reduced dissolution rates and enhanced ORR durability. Similarly, entropy-stabilized oxides and defect-engineered proton-conducting ceramics have shown remarkable thermal and chemical stability under harsh electrochemical environments. Interface stabilization has become another essential strategy for improving long-term electrochemical reliability because electrode/electrolyte interfaces often represent the most chemically unstable regions within fuel cell systems [57]. During prolonged operation, elemental interdiffusion, thermal mismatch, phase segregation, and interfacial reactions can generate highly resistive secondary phases and accelerate structural degradation. To mitigate these effects, advanced barrier-layer deposition techniques are increasingly employed to suppress undesirable interdiffusion and prevent the formation of insulating interfacial compounds. Functionally graded electrodes are also being developed to gradually vary composition and microstructure across the electrode thickness, thereby minimizing abrupt mechanical and thermal mismatches while simultaneously improving charge transport continuity [158]. In addition, carefully engineered buffer layers can enhance interfacial adhesion and accommodate thermal expansion differences between neighboring materials. Controlled interdiffusion strategies have further demonstrated that optimizing rather than completely suppressing elemental diffusion can generate chemically stable and electrochemically active interfaces that facilitate efficient charge-transfer kinetics [159]. Surface passivation approaches involving ultrathin oxide overlayers and chemically stable coatings are similarly utilized to protect reactive interfaces from corrosion, poisoning, and chemical decomposition under aggressive electrochemical conditions. Collectively, these interface engineering strategies significantly improve electrochemical durability by stabilizing ionic transport pathways, reducing interfacial resistance growth, and suppressing long-term structural degradation [160].

Recently, self-healing materials have emerged as highly innovative candidates for future electrochemical energy systems. These materials possess the capability to autonomously repair microcracks, restore ionic transport pathways, and reconstruct damaged interfaces during operation. Several self-healing approaches involving defect migration, redox-assisted reconstruction, thermally activated healing, and mobile secondary phases are currently under investigation. In ceramic electrolytes, oxygen vacancies and mobile dopants can facilitate diffusion-assisted crack healing and structural recovery under elevated temperatures. Although self-healing electrochemical materials remain at an early stage of development, they offer transformative potential for dramatically extending the operational lifetime and reliability of next-generation hydrogen energy technologies.

## 7. Sustainable Applications of Hydrogen and Fuel Cells

Hydrogen energy and fuel cell technologies are increasingly recognized as critical pillars for achieving global decarbonization, sustainable industrial transformation, and long-term energy security. The growing urgency to reduce greenhouse gas emissions, mitigate climate change, and transition away from fossil-fuel-based energy systems has accelerated worldwide investment in hydrogen production, fuel cell deployment, and renewable energy integration [161]. Unlike conventional combustion technologies, hydrogen-based systems enable clean electrochemical energy conversion with high efficiency and minimal pollutant emissions, making them highly attractive for transportation, stationary power generation, industrial decarbonization, portable electronics, and renewable energy storage applications [162].

The versatility of hydrogen as an energy carrier arises from its compatibility with multiple energy sectors, including mobility, electricity generation, chemical manufacturing, and heavy industry. Furthermore, hydrogen technologies can facilitate sector coupling by linking renewable electricity generation with transportation, industrial processing, and long-duration energy storage [163]. Recent advancements in electrocatalysts, electrolyzers, proton-conducting materials, and fuel cell architectures have significantly accelerated the practical deployment of hydrogen technologies across both developed and emerging economies. However, achieving widespread commercialization still requires overcoming challenges related to infrastructure development, production cost, storage efficiency, durability, and large-scale system integration.

### 7.1. Transportation Sector

**Figure 11:**
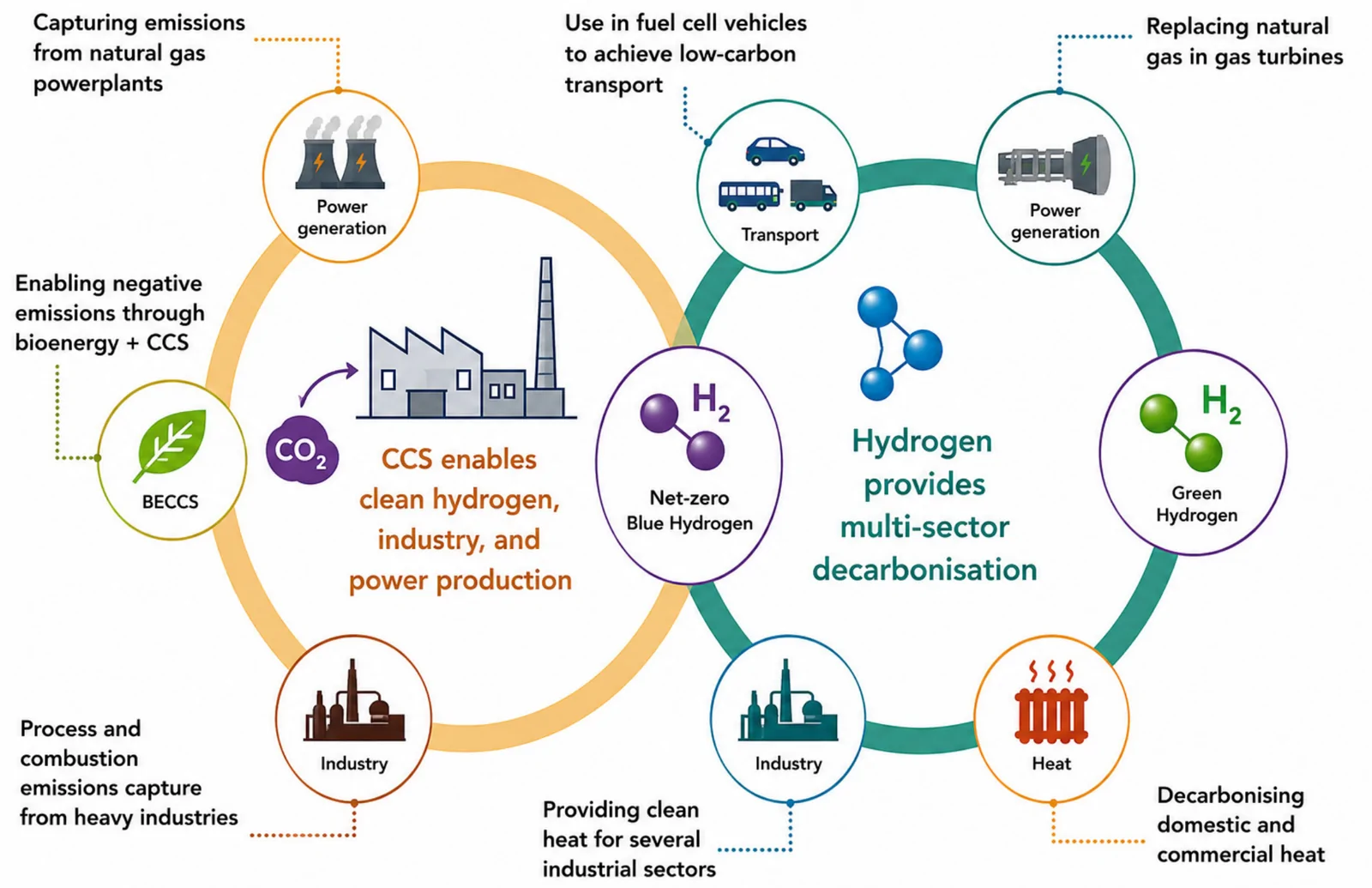
Hydrogen-Based Strategies for Industrial and Energy Sector Decarbonization [172,173].

The transportation sector is one of the largest contributors to global greenhouse gas emissions and fossil fuel consumption. Consequently, hydrogen fuel cell technologies are increasingly being explored as sustainable alternatives to conventional internal combustion engines because they offer zero tailpipe emissions, high efficiency, rapid refueling capability, and long driving range. Fuel cell-based transportation systems are particularly attractive for applications where battery–electric technologies face limitations associated with charging time, energy density, or payload constraints [164]. The major transportation applications of hydrogen and fuel cell technologies are illustrated in Figure 11. Hydrogen-powered systems are increasingly being deployed across passenger vehicles, heavy-duty trucks, railway transportation, mining equipment, aviation, and marine sectors owing to their high energy density, rapid refueling capability, long operational range, and potential for near-zero greenhouse gas emissions [25].

Fuel Cell Electric Vehicles (FCEVs)

Fuel cell electric vehicles (FCEVs) utilize hydrogen fuel cells to generate electricity electrochemically for powering electric motors. Unlike battery–electric vehicles, FCEVs continuously produce electricity as long as hydrogen fuel is supplied, enabling significantly longer driving ranges and faster refueling times [165]. In PEMFC-based vehicles, hydrogen is oxidized at the anode while oxygen reduction occurs at the cathode, producing electricity and water as the primary by-product. The overall electrochemical reaction in FCEVs is:

2*H*_2_ + *O*_2_ → 2*H*_2_*O* + *electrical energy* + *heat* (66)

FCEVs offer several important advantages, including high gravimetric energy density, quiet operation, reduced environmental impact, and rapid refueling typically within 3–5 min. These characteristics make hydrogen vehicles highly competitive for long-distance transportation and commercial fleet applications. Major automotive manufacturers including Toyota, Hyundai, Honda, BMW, and Mercedes-Benz have significantly expanded investments in fuel cell mobility technologies. Commercially available vehicles such as the Toyota Mirai and Hyundai NEXO have demonstrated the technical feasibility of hydrogen-powered passenger transportation [166]. Furthermore, advances in lightweight hydrogen storage tanks, Pt-alloy electrocatalysts, and high-performance membranes have substantially improved driving range and system durability. Despite these advantages, large-scale adoption of FCEVs remains limited by the high cost of hydrogen infrastructure, insufficient refueling networks, and the continued reliance on precious metal catalysts [167]. The economic competitiveness of FCEVs also strongly depends on the development of low-cost green hydrogen production pathways and widespread hydrogen distribution systems.

Heavy-Duty Transport

Hydrogen fuel cells are particularly promising for heavy-duty transportation applications including buses, trucks, rail systems, and mining equipment because these sectors require high energy density, rapid refueling, and long operational range. Battery–electric systems often face challenges related to battery weight, charging duration, and limited range under heavy loads, whereas hydrogen fuel cells can provide continuous high-power operation with comparatively lower system weight [168]. Hydrogen-powered buses and freight trucks have already been deployed in several countries including Germany, China, Japan, South Korea, and the United States. Fuel cell trucks developed by companies such as Nikola, Hyundai, and Daimler are being actively tested for long-haul transportation and logistics operations.

In rail transportation, hydrogen-powered trains such as Alstom’s Coradia iLint have demonstrated successful zero-emission operation on non-electrified railway networks [169]. These systems eliminate diesel emissions while reducing noise pollution and improving operational efficiency. Heavy-duty fuel cell systems generally require highly durable catalysts, large-capacity hydrogen storage systems, and efficient thermal management because of continuous high-load operation. Consequently, ongoing research focuses on improving stack durability, reducing platinum loading, and optimizing fuel cell integration for commercial transport applications.

Aviation and Marine Applications

The aviation and maritime sectors represent some of the most difficult industries to decarbonize because of their extremely high energy demands and reliance on fossil fuels. Hydrogen and fuel cell technologies are therefore increasingly being investigated as potential pathways toward low-carbon aviation and marine transportation. In aviation, hydrogen can be utilized either directly in hydrogen combustion turbines or indirectly through fuel cells powering electric propulsion systems. Liquid hydrogen is particularly attractive for aviation because of its exceptionally high gravimetric energy density. Several aerospace companies including Airbus and Rolls-Royce are actively developing hydrogen-powered aircraft concepts for future commercial deployment. Fuel cell-based electric propulsion systems are especially promising for short-range regional aircraft, drones, and unmanned aerial vehicles because of their high efficiency and low emissions [170]. However, major challenges remain regarding cryogenic hydrogen storage, aircraft redesign, fuel infrastructure, and safety certification.

Similarly, the maritime sector is increasingly exploring hydrogen fuel cells and hydrogen-derived fuels such as ammonia and methanol for decarbonizing shipping operations. Fuel cell-powered ships can significantly reduce CO_2_, SO_x_, and NO_x_ emissions compared to conventional marine diesel engines. Several pilot-scale hydrogen ferries and fuel cell vessels have already demonstrated promising operational performance [171]. Nevertheless, large-scale marine hydrogen deployment still faces substantial challenges related to fuel storage volume, hydrogen liquefaction, infrastructure development, and economic feasibility. Consequently, ammonia and liquid organic hydrogen carriers are increasingly being explored as alternative hydrogen transport fuels for maritime applications.

### 7.2. Stationary Power Generation

**Figure 12:**
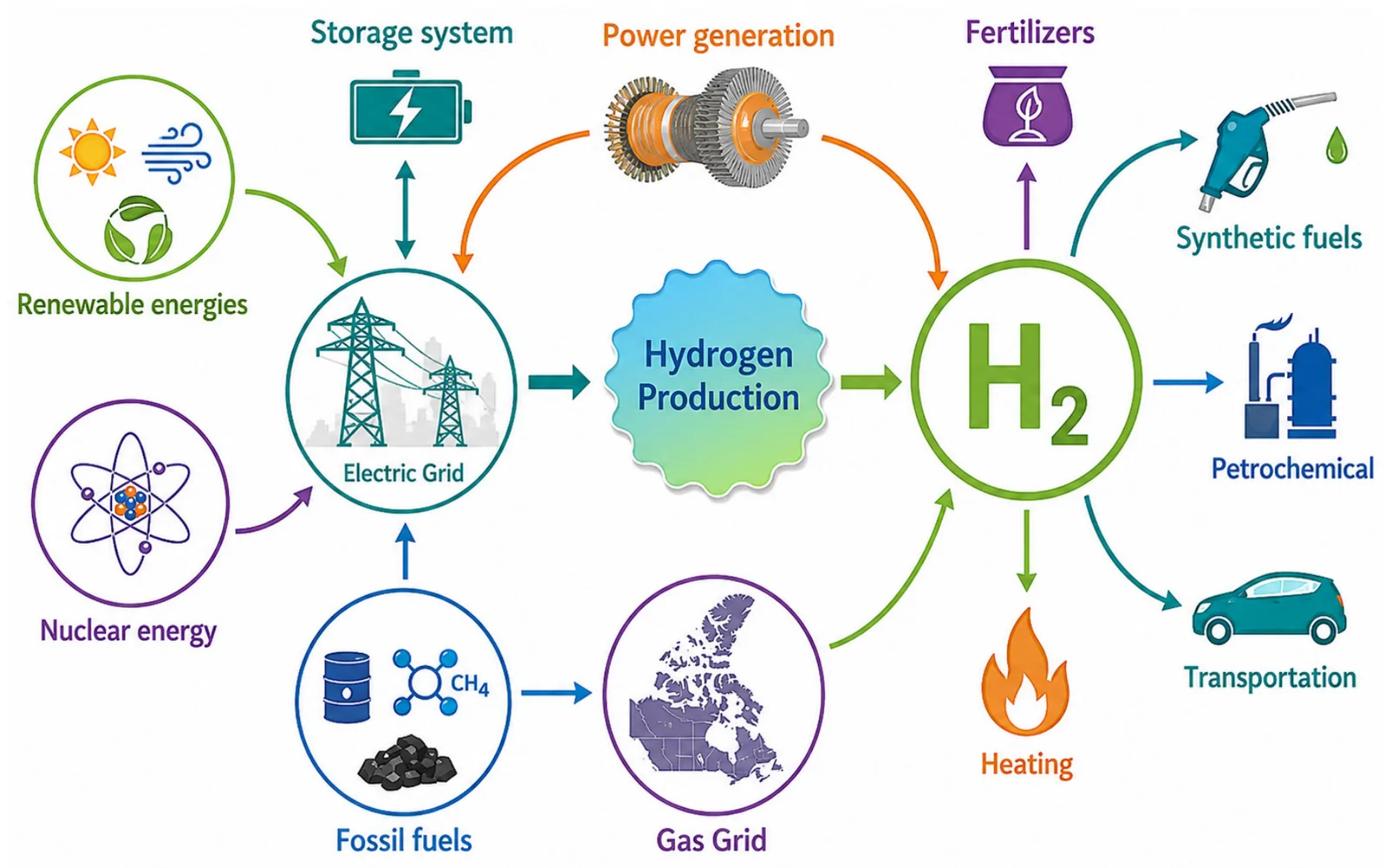
Integrated Hydrogen Production, Storage, and End-Use Applications.

Fuel cells are highly attractive for stationary power generation because they provide high efficiency, low emissions, quiet operation, and continuous electricity production. Stationary fuel cell systems can operate using hydrogen, natural gas, biogas, syngas, or other hydrogen-rich fuels, making them highly versatile energy conversion technologies [174]. The diverse sustainable applications of hydrogen and fuel cell technologies are illustrated in Figure 12. Beyond transportation, hydrogen energy systems are increasingly being deployed in stationary power generation, backup power systems, combined heat and power (CHP) applications, portable and wearable electronics, industrial decarbonization, and renewable energy integration. These emerging applications highlight the versatility of hydrogen as a clean energy carrier capable of supporting decarbonization across multiple sectors.

Distributed Energy Systems

Distributed fuel cell energy systems enable decentralized electricity generation close to end users, thereby reducing transmission losses and improving grid resilience. PEMFCs, SOFCs, and MCFCs are increasingly being utilized for residential, commercial, and industrial distributed power applications. Distributed fuel cell systems offer several advantages, including modularity, high reliability, low noise levels, and efficient integration with renewable energy systems. In addition, fuel cells can provide stable electricity generation independent of intermittent renewable energy fluctuations [175]. SOFC-based distributed systems are particularly attractive because of their high electrical efficiency and fuel flexibility. Their high operating temperatures also enable internal reforming of natural gas and biogas, reducing external fuel processing requirements. Recent developments in microgrids and smart energy systems have further accelerated interest in distributed hydrogen-fuel-cell-based electricity generation because these systems can improve energy security and grid stability in remote or disaster-prone regions.

Backup Power Systems

Fuel cells are increasingly being deployed as backup and emergency power systems for hospitals, telecommunications infrastructure, data centers, military facilities, and critical industrial operations. Unlike diesel generators, fuel cell backup systems produce minimal noise and emissions while providing highly reliable electricity generation. Hydrogen fuel cells offer particularly important advantages for backup applications because they can operate continuously for extended durations as long as hydrogen fuel is available. PEMFCs are especially attractive for backup systems because of their rapid startup and relatively low operating temperature [176]. Several telecommunications companies and data centers have already implemented hydrogen fuel cell systems to ensure uninterrupted power supply during grid outages. Furthermore, fuel cells can significantly improve energy resilience during natural disasters and extreme weather events where conventional power systems may fail.

Combined Heat and Power (CHP) Systems

Combined heat and power (CHP) fuel cell systems simultaneously generate electricity and utilize waste heat for heating or industrial processes, thereby significantly improving overall energy efficiency. The total CHP efficiency can exceed 80% because thermal energy generated during electrochemical operation is recovered rather than wasted. SOFCs and MCFCs are particularly suitable for CHP applications because of their elevated operating temperatures and high-quality heat generation [177]. Fuel-cell-based CHP systems are increasingly being adopted in residential buildings, hospitals, universities, and industrial facilities to reduce energy consumption and greenhouse gas emissions. Furthermore, integration with renewable hydrogen production technologies can enable nearly carbon-neutral distributed energy systems. Despite their advantages, widespread CHP deployment remains limited by high capital costs and infrastructure requirements. Nevertheless, continued improvements in stack durability, system integration, and hydrogen availability are expected to accelerate commercialization.

### 7.3. Portable and Wearable Electronics

Hydrogen fuel cells are increasingly being explored for portable electronics and wearable energy systems because of their high energy density, lightweight design, and extended operational duration compared to conventional batteries.

Micro Fuel Cells

Micro fuel cells are miniaturized electrochemical devices designed for low-power applications including portable electronics, sensors, medical devices, and military systems. These systems often utilize PEMFC or direct methanol fuel cell architectures because of their compact size and relatively low operating temperatures. Micro fuel cells provide several advantages over traditional lithium-ion batteries, including longer operational lifetime, rapid refueling capability, and reduced environmental impact [178]. Their high energy density makes them particularly attractive for applications requiring continuous long-duration power supply. Advances in microfabrication technologies, nanostructured catalysts, and flexible membranes have significantly improved the performance of miniaturized fuel cell systems. However, challenges related to fuel storage, water management, and manufacturing cost remain major barriers to widespread commercialization.

Portable Hydrogen Devices

Portable hydrogen-powered devices are increasingly being developed for military equipment, remote sensing systems, emergency power supplies, and consumer electronics. Portable fuel cell systems can provide lightweight, quiet, and long-duration energy generation under conditions where battery replacement or recharging is impractical. Hydrogen cartridges, chemical hydrides, and miniature storage systems are commonly investigated for portable hydrogen supply. In wearable electronics, flexible fuel cells integrated with lightweight proton-conducting membranes and nanostructured electrodes have shown considerable promise for powering smart textiles, biosensors, and healthcare monitoring devices [179]. Although still at an early stage of development, portable fuel cell technologies are expected to become increasingly important for next-generation autonomous electronic systems and remote energy applications.

### 7.4. Industrial Decarbonization

Hydrogen is increasingly recognized as one of the most important pathways for decarbonizing energy-intensive industrial sectors that are difficult to electrify directly. Industries such as steel production, cement manufacturing, chemical processing, and ammonia synthesis currently account for substantial global CO_2_ emissions because of their heavy reliance on fossil fuels and high-temperature thermal processes.

Steel and Cement Industries

The steel industry is one of the largest industrial emitters of carbon dioxide because conventional blast furnace operations rely heavily on coke as both a reducing agent and energy source. Hydrogen-based direct reduction of iron ore has therefore emerged as a highly promising low-carbon alternative. The reduction reaction can be expressed as:

*Fe*_2_*O*_3_ + 3*H*_2_ → 2*Fe* + 3*H*_2_*O* (67)

Unlike conventional carbon-based reduction processes, hydrogen reduction produces water vapor rather than CO_2_ emissions. Several pilot-scale hydrogen steel production projects are currently being developed in Europe and Asia, including the HYBRIT initiative in Sweden [180]. Similarly, hydrogen can contribute to cement industry decarbonization through clean thermal energy generation and integration with carbon capture technologies. Since cement production involves extremely high-temperature processes, hydrogen combustion and hydrogen-derived fuels may significantly reduce fossil-fuel dependence in cement kilns.

Chemical Manufacturing

Hydrogen is already extensively utilized in chemical manufacturing processes including petroleum refining, methanol production, hydrogenation reactions, and synthetic fuel generation. Transitioning from fossil-fuel-derived hydrogen to green hydrogen can therefore substantially reduce industrial carbon emissions [38]. Green hydrogen integration is particularly important for sustainable synthetic fuel production, carbon-neutral chemicals, and circular carbon economies. In addition, hydrogen can facilitate CO_2_ utilization pathways through catalytic hydrogenation reactions for producing methanol, methane, and hydrocarbon fuels.

Ammonia Production

Ammonia synthesis currently relies heavily on hydrogen derived from natural gas reforming, making the Haber–Bosch process a major source of global CO_2_ emissions. Replacing fossil-fuel-derived hydrogen with renewable green hydrogen could dramatically reduce the carbon footprint of ammonia production. The ammonia synthesis reaction is:

*N*_2_ + 3*H*_2_ → 2*NH*_3_ (68)

Green ammonia is increasingly being explored not only as fertilizer feedstock but also as a potential carbon-free fuel and hydrogen carrier because of its relatively high volumetric hydrogen density and established transportation infrastructure.

### 7.5. Renewable Energy Integration

Hydrogen technologies play a critically important role in integrating intermittent renewable energy sources such as solar and wind into modern energy systems. Because renewable electricity generation is inherently variable, efficient long-duration energy storage technologies are essential for maintaining grid stability and ensuring continuous energy supply [181].

Hydrogen for Grid Balancing

Excess renewable electricity generated during periods of low demand can be utilized for hydrogen production through water electrolysis. The generated hydrogen can subsequently be stored and reconverted into electricity using fuel cells during periods of high energy demand. This approach enables large-scale grid balancing by effectively coupling renewable electricity generation with chemical energy storage. Hydrogen-based grid stabilization is particularly attractive for supporting high renewable-energy penetration while minimizing curtailment losses [182].

Power-to-Gas Technologies

Power-to-gas (P2G) technologies convert renewable electricity into hydrogen or synthetic methane through electrochemical and catalytic processes. Hydrogen generated through electrolysis may either be injected directly into natural gas networks or further reacted with captured CO_2_ to produce methane. The methanation reaction is:

*CO*_2_ + 4*H*_2_ → *CH*_4_ + 2*H*_2_*O* (69)

P2G technologies provide highly attractive pathways for sector coupling because they integrate renewable electricity, gas infrastructure, transportation, and industrial applications into unified energy systems.

Seasonal Energy Storage

One of the most important advantages of hydrogen is its capability for long-duration and seasonal energy storage. Unlike batteries, which are more suitable for short-term storage, hydrogen can store large quantities of renewable energy for weeks or months with relatively low self-discharge losses. Seasonal hydrogen storage can therefore address the mismatch between renewable energy generation and long-term energy demand [183]. Underground hydrogen storage in salt caverns, depleted gas reservoirs, and geological formations is increasingly being investigated for grid-scale energy storage applications. Overall, hydrogen and fuel cell technologies are expected to play transformative roles in enabling sustainable transportation, industrial decarbonization, renewable energy integration, and global carbon-neutral energy systems [184]. Continued advancements in catalyst design, electrolyzer efficiency, hydrogen infrastructure, and fuel cell durability will be essential for accelerating the worldwide transition toward integrated hydrogen economies and future sustainable energy landscapes.

## 8. Techno-Economic Analysis and Sustainability Assessment

The global transition toward hydrogen-based energy systems is strongly influenced not only by scientific and technological advancements but also by economic feasibility, environmental sustainability, policy frameworks, and market readiness. Although hydrogen and fuel cell technologies possess enormous potential for achieving carbon neutrality and sustainable energy transformation, their widespread commercialization depends on reducing production costs, improving lifecycle sustainability, expanding infrastructure, and establishing supportive regulatory environments. Consequently, techno-economic analysis (TEA) and sustainability assessment have become essential tools for evaluating the long-term viability and competitiveness of hydrogen technologies compared with conventional fossil-fuel-based energy systems.

Techno-economic analysis provides a comprehensive understanding of capital investment, operational expenditure, system efficiency, energy consumption, infrastructure requirements, and market competitiveness across the entire hydrogen value chain. Simultaneously, sustainability assessment and life cycle analysis evaluate environmental impacts associated with hydrogen production, storage, transportation, fuel cell manufacturing, and end-use applications [10]. These assessments are critically important because hydrogen technologies are only environmentally beneficial when integrated with low-carbon energy sources and sustainable material supply chains. Furthermore, international hydrogen strategies, government incentives, industrial investments, and carbon neutrality policies are increasingly accelerating global hydrogen deployment and shaping future hydrogen markets.

Despite rapid progress in electrolyzer technologies, fuel cell systems, renewable energy integration, and hydrogen infrastructure development, several major barriers continue to hinder commercialization. Challenges related to high production cost, limited infrastructure, scalability constraints, material availability, and long-term system durability remain significant obstacles that must be addressed before hydrogen can achieve widespread global adoption [185]. Therefore, integrated techno-economic and sustainability analyses are essential for identifying viable hydrogen pathways and guiding future research, policy, and industrial investment strategies.

### 8.1. Cost Analysis of Hydrogen Technologies

**Figure 13:**
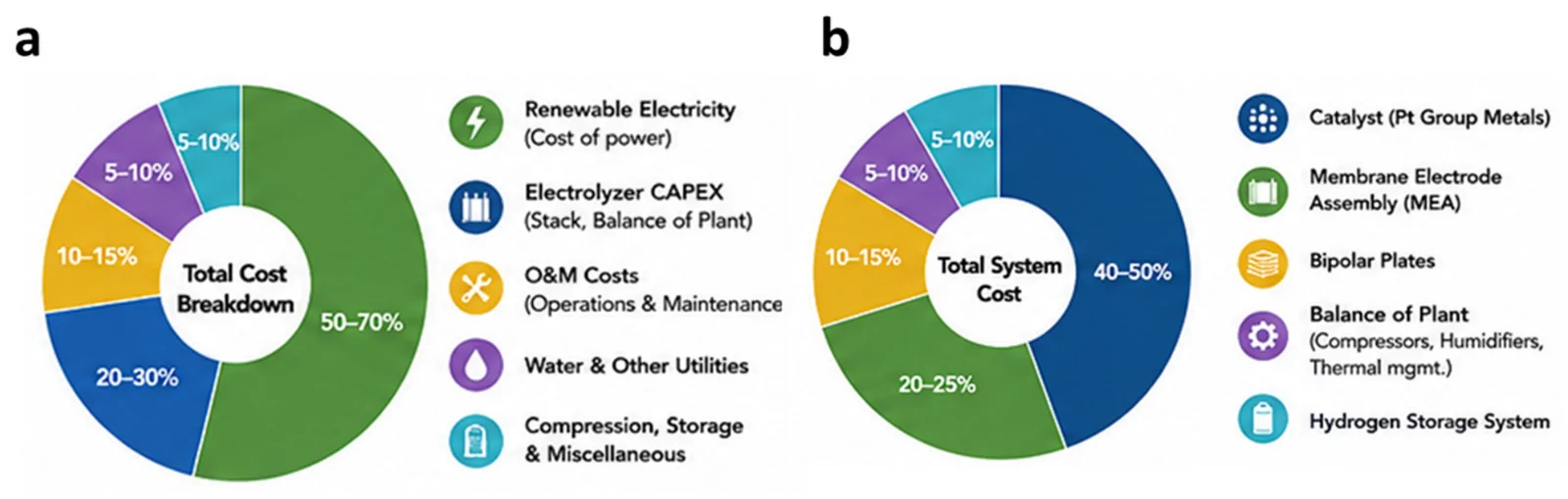
(**a**) Cost distribution of green hydrogen production via electrolysis; (**b**) fuel cell system cost breakdown highlighting major cost-contributing components [192,193].

The economic competitiveness of hydrogen technologies remains one of the most critical factors determining their commercial adoption and large-scale deployment. Hydrogen production, storage, transportation, and fuel cell systems currently remain more expensive than many conventional fossil-fuel-based technologies in several regions of the world. However, declining renewable electricity costs, improvements in electrolyzer efficiency, advancements in catalyst development, and increasing industrial-scale production are gradually improving the economic feasibility of hydrogen energy systems. The major economic factors influencing hydrogen commercialization are illustrated in Figure 13a,b, including the cost structure of green hydrogen production, fuel cell system cost distribution, and comparative levelized hydrogen production costs for different hydrogen pathways.

Hydrogen production cost strongly depends on feedstock availability, electricity price, electrolyzer efficiency, infrastructure investment, operating conditions, and carbon management strategies. Conventional grey hydrogen produced through steam methane reforming remains the least expensive production route because of mature industrial infrastructure and low natural gas prices. However, this pathway generates substantial carbon dioxide emissions and therefore lacks long-term environmental sustainability [186]. Blue hydrogen partially reduces emissions through carbon capture and storage integration, although the overall economics strongly depend on carbon capture efficiency, methane leakage mitigation, and transportation infrastructure. In contrast, green hydrogen produced via renewable-energy-powered water electrolysis offers the most environmentally sustainable pathway but currently exhibits higher production costs because of the electricity-intensive nature of electrolysis and the relatively high capital cost of electrolyzers. The economic performance of hydrogen production systems is commonly evaluated using the levelized cost of hydrogen (LCOH):

(70) $$\mathrm{LCOH}=\frac{\mathrm{CAPEX}+\mathrm{OPEX}+\mathrm{Energy}\;\mathrm{Costs}}{\mathrm{Total}\;\mathrm{Hydrogen}\;\mathrm{Produced}}$$

where CAPEX represents capital expenditure, OPEX denotes operational expenditure, and energy costs correspond to electricity or fuel consumption during hydrogen production.

Renewable electricity price is one of the most dominant factors affecting green hydrogen economics because electrolysis requires substantial electrical energy input. Consequently, regions possessing abundant solar and wind resources are expected to become major global hydrogen production hubs in the future [187]. Electrolyzer technology also significantly influences overall hydrogen cost. Alkaline electrolyzers currently offer lower capital costs because of technological maturity and the use of non-precious metal catalysts, whereas proton exchange membrane electrolyzers provide superior operational flexibility and high current density but require expensive platinum-group metals such as platinum and iridium. Solid oxide electrolysis cells demonstrate exceptionally high theoretical efficiencies because elevated operating temperatures reduce electrical energy demand; however, thermal degradation and material instability still limit commercial deployment [188].

Fuel cell system cost similarly remains a major barrier to widespread commercialization, particularly in transportation and stationary power generation applications. Fuel cell economics are strongly influenced by catalyst materials, electrolyte membranes, bipolar plates, thermal management systems, hydrogen storage units, and stack manufacturing complexity. Among these, platinum-based catalysts contribute substantially to PEMFC cost because of their high catalytic activity and limited global supply. Although catalyst loading has decreased significantly in recent years through nanostructured catalyst engineering and improved catalyst utilization efficiency, precious metal dependence continues to present economic challenges for large-scale deployment. Fuel cell system economics are commonly evaluated using cost-per-power metrics:

(71) $$\mathrm{Cost}\;\mathrm{per}\;\mathrm{kW}=\frac{\mathrm{Total}\;\mathrm{System}\;\mathrm{Cost}}{\mathrm{Power}\;\mathrm{Output}}$$

SOFC systems additionally face economic challenges associated with high-temperature ceramic processing, thermal insulation, interconnect materials, and sealing technologies. Nevertheless, their superior efficiency and fuel flexibility provide important long-term economic advantages for stationary and combined heat and power applications. Operational durability strongly affects overall system economics because degradation-induced maintenance and replacement costs significantly increase lifecycle expenditure [189]. Consequently, improving long-term electrochemical stability is critically important for reducing total ownership cost and improving market competitiveness. Future cost reductions are expected through mass production, manufacturing automation, modular stack design, improved recycling technologies, and the development of low-platinum or platinum-free electrocatalysts.

### 8.2. Life Cycle Assessment (LCA)

Life cycle assessment has become one of the most important methodologies for evaluating the environmental sustainability of hydrogen and fuel cell technologies because it provides a comprehensive analysis of energy consumption, emissions, resource utilization, and ecological impact throughout the entire lifecycle of the system. Unlike conventional operational emission analysis, life cycle assessment considers all stages including raw material extraction, catalyst synthesis, component manufacturing, hydrogen production, transportation, operation, and end-of-life disposal or recycling.

Hydrogen technologies are frequently considered environmentally benign because fuel cells primarily produce water during operation. However, the overall environmental performance strongly depends on the hydrogen production pathway, electricity source, infrastructure requirements, and material processing conditions. Consequently, lifecycle analysis is critically important for distinguishing truly sustainable hydrogen pathways from those associated with significant indirect environmental impacts.

Life cycle assessment studies commonly evaluate several environmental indicators to comprehensively assess sustainability performance. Among these, global warming potential is one of the most important parameters because it quantifies the greenhouse gas emissions generated throughout the hydrogen value chain in terms of CO_2_ equivalent emissions [190]. In addition, acidification potential evaluates the contribution of industrial emissions such as sulfur oxides and nitrogen oxides to acid rain formation and ecosystem degradation. Eutrophication potential is similarly important because nutrient-rich emissions associated with energy generation and industrial processing may lead to excessive algal growth and severe aquatic ecosystem imbalance. Water consumption has also become an increasingly critical concern, particularly for electrolysis-based hydrogen production systems that require substantial quantities of purified water and electricity. Resource depletion analysis further evaluates the sustainability of raw material utilization because many hydrogen technologies rely on critical materials including platinum-group metals, rare-earth elements, cobalt, nickel, and zirconium. Furthermore, human toxicity assessments analyze the potential health risks associated with mining activities, catalyst synthesis, industrial emissions, and material processing across the hydrogen lifecycle. Consequently, comprehensive lifecycle assessment methodologies are essential for identifying environmentally sustainable hydrogen technologies while minimizing ecological damage, resource depletion, and long-term environmental risk [191].

The carbon footprint of hydrogen production pathways varies substantially depending on energy source and technological configuration. Grey hydrogen generated from fossil fuels exhibits high carbon intensity because large quantities of carbon dioxide are released during methane reforming and coal gasification. Blue hydrogen partially reduces emissions through carbon capture integration; however, methane leakage and incomplete carbon capture remain significant environmental concerns. In contrast, green hydrogen produced through renewable-energy-powered electrolysis demonstrates the lowest lifecycle carbon emissions and offers the greatest potential for achieving carbon-neutral energy systems.

The carbon intensity of hydrogen production is commonly expressed as:

(72) $$\mathrm{Carbon}\;\mathrm{Intensity}=\frac{{\mathrm{CO}}_{2}\;\mathrm{Emissions}}{\mathrm{kg}\;H_{2}\;\mathrm{Produced}}$$

Hydrogen storage and transportation systems also contribute significantly to lifecycle emissions because compression, liquefaction, cryogenic storage, and long-distance transport require substantial energy input. Similarly, manufacturing of fuel cells and electrolyzers requires energy-intensive processing of ceramics, metals, catalysts, and advanced membranes. Consequently, future sustainable hydrogen systems must emphasize not only low-carbon hydrogen production but also recyclable materials, energy-efficient manufacturing, and circular economy integration. Recent lifecycle assessment studies have highlighted the importance of integrating renewable electricity with hydrogen production to maximize environmental benefits. In addition, catalyst recycling, sustainable material sourcing, and closed-loop manufacturing strategies are increasingly recognized as essential approaches for minimizing long-term ecological impact and improving overall sustainability.

### 8.3. Policy, Regulations, and Market Trends

Government policies, international regulations, industrial investments, and market strategies are playing increasingly important roles in accelerating the commercialization of hydrogen technologies. Because hydrogen infrastructure development requires substantial capital investment and long-term planning, supportive policy frameworks are essential for reducing economic risk, stimulating market growth, and encouraging private-sector participation.

Many countries and international organizations have introduced comprehensive hydrogen roadmaps and national hydrogen strategies aimed at achieving carbon neutrality and energy transition objectives. These roadmaps generally focus on renewable hydrogen production, fuel cell deployment, industrial decarbonization, renewable energy integration, hydrogen mobility, and infrastructure expansion. The European Union has emerged as a global leader in hydrogen policy through initiatives such as the European Hydrogen Strategy and REPowerEU framework, both of which prioritize large-scale green hydrogen deployment and renewable energy integration. Similarly, Japan and South Korea have implemented national hydrogen strategies emphasizing hydrogen mobility, fuel cell commercialization, and hydrogen import infrastructure [194]. China has also rapidly expanded investment in fuel cell transportation, electrolyzer manufacturing, and industrial hydrogen applications, while the United States has accelerated clean hydrogen deployment through initiatives such as the Hydrogen Shot program and Inflation Reduction Act incentives.

Government support mechanisms are critically important during the early stages of hydrogen commercialization because they help overcome high capital costs and market uncertainty. Several policy instruments are currently being implemented worldwide, including carbon pricing systems, hydrogen production subsidies, tax incentives, infrastructure funding programs, renewable energy mandates, and public–private partnerships. Massive investments are being directed toward electrolyzer manufacturing facilities, hydrogen pipelines, fuel cell production plants, and refueling infrastructure. Hydrogen valleys and integrated hydrogen hubs are increasingly being established to demonstrate large-scale hydrogen ecosystems involving production, storage, transportation, and end-use integration within coordinated regional networks. These projects aim to accelerate technological validation, infrastructure development, and industrial collaboration while creating economically viable hydrogen supply chains. Private-sector participation in hydrogen technologies is also expanding rapidly [195]. Major energy companies, automotive manufacturers, chemical industries, steel producers, and utility providers are investing heavily in hydrogen production, fuel cell systems, renewable ammonia, and synthetic fuel technologies as part of long-term decarbonization strategies. Market projections indicate substantial growth in global hydrogen demand over the coming decades, particularly in transportation, heavy industry, renewable energy storage, and chemical manufacturing sectors.

### 8.4. Challenges Toward Commercialization

Despite remarkable technological progress and increasing global investment, several major barriers continue to hinder the widespread commercialization of hydrogen and fuel cell technologies. These challenges involve infrastructure limitations, scalability constraints, material availability, economic competitiveness, and long-term system durability. Infrastructure development remains one of the largest obstacles to establishing hydrogen economies because comprehensive hydrogen systems require extensive production plants, storage facilities, pipelines, transportation networks, and refueling stations. Currently, hydrogen refueling infrastructure remains insufficient in many countries, limiting practical deployment of fuel cell vehicles and hydrogen mobility systems. Similarly, large-scale hydrogen transportation and storage infrastructure require substantial investment and coordinated international planning. Hydrogen pipelines face challenges related to hydrogen embrittlement, leakage, material compatibility, and safety regulation, while cryogenic hydrogen systems require highly energy-intensive liquefaction and advanced thermal insulation technologies. The absence of globally standardized hydrogen regulations and certification systems further complicates international hydrogen trade and infrastructure harmonization.

Scalability also represents a major challenge because expanding hydrogen technologies from laboratory research to industrial-scale deployment requires massive growth in electrolyzer manufacturing capacity, renewable electricity generation, fuel cell production volume, and hydrogen distribution infrastructure. Green hydrogen economies are particularly dependent on the large-scale expansion of solar, wind, hydroelectric, and other renewable energy systems capable of supplying sufficient low-cost electricity for electrolysis. Simultaneously, fuel cell manufacturing requires substantial improvements in production scalability, catalyst utilization efficiency, stack durability, and system integration before large-scale commercialization can be achieved.

Resource availability presents another critical challenge because many electrolyzers and fuel cells rely on scarce or geopolitically sensitive materials such as platinum, iridium, cobalt, nickel, and rare-earth elements. PEM electrolyzers and PEM fuel cells are especially dependent on platinum-group metals whose limited global supply and high market volatility may create long-term supply-chain constraints. Iridium availability is similarly considered a major concern for future PEM electrolyzer deployment because global iridium production remains extremely limited. Consequently, extensive research efforts are currently focused on developing platinum-free catalysts, earth-abundant electrocatalysts, recyclable materials, high-entropy compounds, and low-criticality electrochemical systems. Overall, although hydrogen and fuel cell technologies possess transformative potential for achieving sustainable energy systems and global decarbonization, their widespread commercialization requires overcoming significant economic, infrastructural, technological, and resource-related barriers. Continued advancements in catalyst engineering, renewable energy integration, scalable manufacturing, lifecycle sustainability, and policy support will therefore play decisive roles in accelerating the global transition toward integrated hydrogen economies and carbon-neutral energy systems.

## 9. Emerging Trends and Future Perspectives

Hydrogen energy and fuel cell technologies are rapidly evolving toward highly integrated, intelligent, and digitally optimized energy systems capable of supporting future carbon-neutral societies. In recent years, substantial progress in artificial intelligence, machine learning, advanced materials engineering, smart energy management, and digitalization has fundamentally transformed the development and optimization of hydrogen technologies. Simultaneously, increasing global commitments toward decarbonization, renewable energy integration, and sustainable industrial transformation are accelerating the deployment of next-generation hydrogen infrastructures with improved efficiency, durability, and economic viability. Future hydrogen economies are therefore expected to emerge through the convergence of electrochemistry, materials science, computational modeling, artificial intelligence, renewable energy systems, and digital engineering.

Traditional approaches for catalyst development, electrochemical optimization, and system integration have often relied heavily on time-consuming experimental trial-and-error methodologies. However, recent advancements in data-driven modeling, computational materials discovery, and intelligent energy management are enabling significantly faster development of highly efficient hydrogen systems. Furthermore, future hydrogen infrastructures are increasingly expected to integrate renewable electricity generation, digital energy platforms, large-scale energy storage, autonomous control systems, and hybrid electrochemical architectures into highly adaptive and interconnected smart energy ecosystems.

Despite remarkable progress, major scientific and technological challenges remain unresolved. Issues related to catalyst durability, hydrogen storage, electrolyzer cost, infrastructure scalability, and long-term operational stability continue to limit widespread commercialization. Consequently, future research efforts are increasingly focusing on ultra-durable catalytic systems, low-cost electrolyzers, intelligent hydrogen networks, sustainable hydrogen storage materials, and integrated renewable hydrogen technologies capable of enabling large-scale global energy transformation.

### 9.1. Artificial Intelligence and Machine Learning in Materials Discovery

Artificial intelligence and machine learning are increasingly revolutionizing the discovery and optimization of electrochemical energy materials by enabling rapid identification of highly efficient catalysts, electrolytes, membranes, and functional energy systems. Conventional experimental approaches for catalyst development are often expensive, labor-intensive, and limited by slow material screening processes. In contrast, machine learning algorithms can rapidly analyze enormous experimental and computational datasets to identify hidden relationships between material composition, crystal structure, defect chemistry, electronic configuration, and electrochemical performance.

Data-driven catalyst design has emerged as one of the most transformative developments in modern hydrogen energy research. By integrating experimental databases with density functional theory calculations, machine learning models can predict catalytic activity, adsorption energies, stability, electronic structure, and reaction kinetics for thousands of candidate materials within a relatively short computational timeframe. These approaches are significantly accelerating the discovery of advanced electrocatalysts including atomically dispersed single-site catalytic systems, entropy-stabilized oxide materials, MXene-derived catalytic interfaces, perovskite-based mixed ionic–electronic conductors, transition-metal phosphides and nitrides, and defect-engineered nanostructured electrocatalysts with optimized adsorption characteristics and enhanced reaction kinetics.

Machine learning frameworks are also increasingly used to establish predictive relationships between electrochemical activity and material descriptors such as d-band center position, coordination environment, vacancy concentration, surface strain, and electronic density distribution. These predictive models enable rational optimization of catalyst composition and surface structure for electrochemical reactions including hydrogen evolution, oxygen evolution, and oxygen reduction processes. Consequently, AI-assisted catalyst screening substantially reduces experimental workload by prioritizing only the most promising materials for synthesis and electrochemical validation. In addition to catalyst discovery, predictive modeling using artificial intelligence is becoming increasingly important for understanding electrochemical degradation, system aging, and long-term operational stability. Neural networks, deep learning algorithms, random forest models, and Gaussian process regression techniques are now widely employed to predict fuel cell degradation behavior, electrolyzer efficiency, hydrogen production rates, thermal instability, and electrochemical impedance characteristics under realistic operating conditions. Artificial intelligence can also optimize operational parameters such as temperature, humidity, pressure, gas flow rates, and current density to maximize electrochemical efficiency while minimizing degradation.

Recent developments involving autonomous robotic laboratories integrated with machine learning algorithms are further transforming hydrogen materials research. These self-optimizing systems are capable of independently synthesizing, characterizing, testing, and optimizing electrochemical materials through closed-loop experimentation with minimal human intervention. Although challenges related to data quality, dataset standardization, model interpretability, and experimental reproducibility still remain, artificial intelligence is expected to become one of the most influential technological drivers shaping the future of hydrogen energy systems and electrochemical materials discovery.

### 9.2. Digitalization and Smart Hydrogen Systems

Digitalization is increasingly transforming hydrogen energy systems through the integration of advanced sensing technologies, real-time monitoring platforms, cloud computing, smart control algorithms, and artificial intelligence-assisted energy management. Future hydrogen infrastructures are expected to operate as highly interconnected smart systems capable of autonomously optimizing hydrogen production, storage, transportation, and utilization in response to dynamic energy demand and renewable electricity availability. The integration of digital technologies with hydrogen systems enables continuous monitoring of electrochemical performance, thermal behavior, gas flow dynamics, degradation rates, and infrastructure reliability. Real-time operational data obtained from sensors and monitoring platforms can be processed using machine learning algorithms to improve system efficiency, identify abnormal operating conditions, predict component failure, and optimize maintenance scheduling. Consequently, digitalization significantly improves operational reliability, reduces downtime, and enhances overall system durability.

One of the most promising developments in this area is the emergence of digital twin technology. Digital twins are virtual representations of physical hydrogen systems that continuously integrate real-time operational data with computational simulations and predictive algorithms. In hydrogen and fuel cell applications, digital twins can simulate electrochemical reactions, thermal transport, catalyst degradation, hydrogen consumption patterns, gas diffusion, and mechanical stress evolution under realistic operating conditions. These virtual models enable predictive maintenance, fault diagnosis, performance optimization, and operational forecasting before actual system failure occurs. Digital twins are increasingly being explored for fuel cell vehicles, hydrogen refueling stations, electrolyzer plants, renewable hydrogen hubs, and industrial hydrogen infrastructures because they offer powerful tools for improving reliability, reducing operational cost, and maximizing system lifetime.

Simultaneously, smart hydrogen energy networks are emerging as critical components of future carbon-neutral energy infrastructures. These intelligent networks integrate hydrogen production, renewable electricity generation, energy storage systems, fuel cells, transportation infrastructure, and digital energy management into interconnected and adaptive energy ecosystems. Smart hydrogen networks can dynamically balance renewable electricity fluctuations, optimize hydrogen generation scheduling, coordinate distributed power systems, and manage long-duration energy storage requirements in real time. Hydrogen is particularly attractive for future smart grids because it enables efficient coupling between electricity generation, industrial processing, transportation systems, and thermal energy sectors. Advanced digital energy platforms utilizing artificial intelligence, industrial internet-of-things technologies, blockchain systems, and cloud-based optimization algorithms are increasingly being developed to enable decentralized energy trading, secure hydrogen distribution, predictive grid balancing, and autonomous infrastructure management. Consequently, digitalization is expected to play a decisive role in enabling highly efficient, resilient, and flexible hydrogen economies capable of supporting large-scale renewable energy integration and future sustainable energy systems.

### 9.3. Integrated Hydrogen Energy Systems

Integrated hydrogen energy systems are increasingly recognized as fundamental components of future sustainable energy infrastructures because they enable direct coupling between renewable electricity generation, hydrogen production, energy storage, transportation, and industrial utilization. These integrated systems provide highly flexible pathways for overcoming renewable energy intermittency while simultaneously supporting decarbonization across multiple sectors of the global economy. The coupling of hydrogen production with solar and wind energy has become one of the most promising approaches for achieving carbon-neutral hydrogen generation. Renewable electricity generated during periods of excess solar irradiation or strong wind conditions can be utilized for water electrolysis to produce green hydrogen. The electrochemical reaction associated with renewable hydrogen production is:

2*H*_2_*O* → *H*_2_ + *O*_2_ (73)

This approach enables efficient conversion of intermittent renewable electricity into stable chemical energy carriers that can be stored, transported, and utilized when energy demand increases or renewable electricity generation decreases. Consequently, renewable hydrogen systems provide important solutions for grid balancing, long-duration energy storage, and renewable energy curtailment reduction. Integrated renewable hydrogen systems are particularly attractive because hydrogen can subsequently be utilized in multiple sectors including transportation, fuel cell electricity generation, industrial decarbonization, synthetic fuel production, and chemical manufacturing. In regions with abundant solar and wind resources, decentralized renewable hydrogen production can additionally improve energy independence while reducing dependence on fossil-fuel-based infrastructure.

Hybrid fuel cell–battery systems are also emerging as highly promising integrated energy architectures because they combine the complementary advantages of both electrochemical technologies. Fuel cells provide continuous high-energy-density electricity generation, whereas batteries offer rapid transient response, high-power-density output, and efficient regenerative energy storage. In transportation systems, hybrid architectures enable efficient management of varying power demand during acceleration, cruising, regenerative braking, and transient loading conditions. Batteries can supply short-duration peak power while fuel cells maintain stable long-term electricity generation, thereby reducing electrochemical degradation and improving overall energy efficiency. Hybrid fuel cell–battery systems are increasingly being explored for transportation, aerospace systems, marine propulsion, microgrids, distributed power generation, and renewable energy storage applications. Advanced energy management algorithms, artificial intelligence-assisted optimization, and high-performance power electronics are becoming critically important for maximizing the operational efficiency, durability, and reliability of these integrated hydrogen energy systems.

### 9.4. Future Research Opportunities

Although remarkable progress has been achieved in hydrogen production, fuel cells, electrolyzers, and electrochemical energy systems, several major scientific and technological challenges remain unresolved. Future research efforts must therefore focus on improving catalyst durability, reducing system cost, enhancing electrochemical efficiency, developing sustainable hydrogen storage technologies, and enabling scalable hydrogen infrastructures. One of the most important future research directions involves the development of ultra-durable electrocatalysts capable of maintaining high electrochemical activity under aggressive operating conditions for prolonged operational periods. Catalyst degradation remains one of the primary factors limiting the durability of fuel cells and electrolyzers because catalyst dissolution, nanoparticle agglomeration, surface reconstruction, and corrosion progressively reduce electrochemical activity. Consequently, future catalyst research is increasingly emphasizing advanced atomic-scale engineering strategies including defect modulation, strain engineering, entropy stabilization, self-reconstructing catalytic interfaces, and atomically dispersed active sites designed to maximize catalytic utilization while improving structural stability. Particular attention is also being directed toward replacing expensive platinum-group metals with earth-abundant catalytic alternatives capable of delivering comparable electrochemical performance. Another critically important research priority involves the development of low-cost electrolyzers for large-scale green hydrogen production. Current electrolyzer technologies remain relatively expensive because of costly catalyst materials, membrane components, and manufacturing complexity. Future electrolyzer systems must therefore achieve significantly lower capital cost, improved durability, high-current-density operation, and superior compatibility with fluctuating renewable electricity sources. Emerging technologies such as anion exchange membrane electrolyzers are attracting considerable interest because they combine advantages of alkaline and PEM systems while potentially reducing reliance on precious metal catalysts. Advanced manufacturing approaches including additive manufacturing, roll-to-roll processing, automated fabrication, and scalable nanostructured electrode synthesis are also expected to play important roles in reducing future electrolyzer cost.

Sustainable hydrogen storage solutions similarly represent one of the most significant future research challenges because hydrogen possesses extremely low volumetric density under ambient conditions. Conventional compressed and cryogenic storage methods remain energy-intensive and economically challenging for large-scale deployment. Consequently, future hydrogen storage research is increasingly focused on advanced solid-state storage systems, metal hydrides, chemical hydrides, liquid organic hydrogen carriers, ammonia-based storage technologies, and nanostructured porous materials capable of reversible hydrogen adsorption and high storage capacity. Materials such as metal–organic frameworks, graphene-derived nanostructures, and high-surface-area porous carbons are particularly promising because of their potential for lightweight, reversible, and energy-efficient hydrogen storage. Future hydrogen research is also expected to increasingly emphasize circular economy integration, recyclable electrochemical materials, low-criticality catalyst systems, sustainable manufacturing, and environmentally benign energy infrastructures. Furthermore, interdisciplinary collaboration involving artificial intelligence, digitalization, electrochemistry, renewable energy engineering, and advanced materials science will be essential for overcoming current technological limitations and enabling the widespread commercialization of integrated hydrogen energy systems capable of supporting future carbon-neutral societies.

## 10. Conclusions

Hydrogen energy and fuel cell technologies have emerged as some of the most promising solutions for addressing the interconnected global challenges of climate change, greenhouse gas emissions, fossil fuel depletion, and long-term energy sustainability. The growing international emphasis on carbon neutrality, renewable energy integration, and sustainable industrial transformation has significantly accelerated scientific research, technological innovation, and industrial investment in hydrogen-based energy systems. As discussed throughout this review, substantial advancements have been achieved in hydrogen production technologies, electrocatalyst engineering, electrochemical reaction mechanisms, proton- and oxygen-ion-conducting materials, fuel cell architectures, system durability, digitalization, and integrated renewable hydrogen infrastructures. These developments collectively demonstrate the enormous potential of hydrogen technologies for enabling future clean energy economies and supporting large-scale decarbonization across transportation, power generation, industrial processing, and energy storage sectors.

One of the most significant advances in recent years has been the remarkable progress in electrocatalyst design and electrochemical materials engineering. Advanced catalyst systems involving nanostructured noble metals, transition-metal phosphides, MXene-derived materials, perovskite oxides, defect-engineered electrocatalysts, and single-atom catalytic architectures have demonstrated substantial improvements in hydrogen evolution, oxygen evolution, and oxygen reduction reaction kinetics. Simultaneously, major developments in proton-conducting ceramics, solid oxide electrolytes, mixed ionic–electronic conductors, and membrane technologies have significantly enhanced ionic conductivity, electrochemical efficiency, and intermediate-temperature fuel cell performance. The integration of operando characterization techniques, synchrotron analysis, advanced microscopy, and computational modeling has further transformed the understanding of electrochemical reaction pathways, catalyst reconstruction mechanisms, degradation phenomena, and ionic transport behavior under realistic operating conditions.

Significant progress has also been achieved in renewable hydrogen production technologies and integrated energy systems. Green hydrogen generated through renewable-energy-powered water electrolysis is increasingly recognized as one of the most sustainable pathways for achieving carbon-neutral energy systems. Coupling hydrogen production with solar and wind energy offers highly attractive solutions for renewable energy storage, grid balancing, and long-duration energy management. Furthermore, advances in fuel cell electric vehicles, distributed fuel cell systems, industrial hydrogen utilization, and hydrogen-powered heavy transportation demonstrate the growing technological maturity and practical feasibility of hydrogen-based energy applications. The emergence of hybrid fuel cell–battery systems, digital hydrogen infrastructures, artificial intelligence-assisted optimization, and smart energy networks further highlights the ongoing transition toward highly interconnected and intelligent hydrogen ecosystems.

Despite these remarkable achievements, several critical scientific, technological, economic, and infrastructural challenges still remain unresolved. One of the most significant limitations continues to be the high cost associated with hydrogen production, electrolyzer systems, fuel cell stacks, hydrogen storage infrastructure, and precious metal catalysts. Although substantial reductions in catalyst loading and manufacturing cost have been achieved, large-scale commercialization still requires economically competitive alternatives to platinum-group metals and other critical raw materials. The limited availability and geopolitical sensitivity of materials such as platinum, iridium, cobalt, and rare-earth elements may also create long-term supply-chain constraints for future hydrogen economies.

Durability and long-term operational stability remain equally important challenges. Catalyst degradation, carbon corrosion, electrolyte decomposition, thermal instability, interfacial degradation, and mechanical failure continue to limit the operational lifetime of fuel cells and electrolyzers under realistic working conditions. In particular, the dynamic reconstruction of catalyst surfaces, nanoparticle agglomeration, redox instability, and degradation induced by startup–shutdown cycles require further mechanistic understanding and advanced mitigation strategies. Consequently, future research must continue emphasizing ultra-durable electrocatalysts, self-healing materials, interface stabilization, defect engineering, and advanced protective architectures capable of maintaining electrochemical performance over extended operational lifetimes.

Hydrogen storage and transportation also remain among the most critical technological barriers limiting large-scale deployment. The inherently low volumetric density of hydrogen under ambient conditions necessitates energy-intensive compression, liquefaction, or advanced storage systems. Although solid-state hydrogen storage materials, metal hydrides, chemical hydrides, liquid organic hydrogen carriers, and ammonia-based storage systems have demonstrated considerable promise, further improvements in storage capacity, reversibility, safety, and economic feasibility are still required. In parallel, large-scale hydrogen distribution infrastructure involving pipelines, refueling stations, storage terminals, and integrated hydrogen hubs requires substantial global investment and coordinated international policy support. Another major challenge involves the scalability and sustainability of future hydrogen systems. Large-scale green hydrogen production will require enormous renewable electricity generation capacity together with highly durable, efficient, and low-cost electrolyzers. Simultaneously, ensuring lifecycle sustainability and minimizing environmental impact require careful consideration of material extraction, manufacturing processes, water consumption, recycling strategies, and circular economy integration. Consequently, future hydrogen technologies must not only demonstrate high electrochemical performance but also achieve long-term environmental compatibility and resource sustainability.

Artificial intelligence, machine learning, digitalization, and smart energy management are expected to play transformative roles in addressing many of these challenges. Data-driven catalyst discovery, predictive degradation modeling, digital twins, autonomous optimization systems, and intelligent hydrogen networks are increasingly accelerating materials development and system-level optimization. The integration of advanced computational tools with experimental electrochemistry and renewable energy systems is therefore likely to significantly shorten development cycles and improve the overall efficiency and reliability of future hydrogen infrastructures.

Looking forward, the future hydrogen economy will likely depend on the successful integration of renewable energy systems, advanced electrochemical technologies, intelligent digital infrastructures, sustainable manufacturing, and global policy coordination. Hydrogen is uniquely positioned to serve as a universal clean energy carrier capable of linking electricity generation, transportation, industrial manufacturing, chemical synthesis, and large-scale energy storage into unified carbon-neutral energy systems. Continued interdisciplinary collaboration among materials scientists, electrochemists, engineers, policymakers, computational researchers, and industrial stakeholders will therefore be essential for overcoming current technological and economic barriers. Overall, hydrogen and fuel cell technologies possess extraordinary potential to fundamentally reshape future global energy systems and accelerate the worldwide transition toward sustainable and low-carbon societies. Continued advancements in catalyst engineering, electrolyzer development, fuel cell durability, hydrogen storage, renewable integration, artificial intelligence-assisted optimization, and infrastructure expansion are expected to drive the emergence of highly efficient, economically viable, and environmentally sustainable hydrogen economies in the coming decades.

## Figures and Tables

**Table 1:** Comparison of Major Water Electrolysis Technologies for Hydrogen Production.

Electrolysis Technology	Electrolyte Type	Operating Temperature	Charge Carrier
Alkaline Water Electrolysis (AWE)	Liquid alkaline solution (KOH/NaOH)	60–90 °C	OH^−^ ions
Proton Exchange Membrane Water Electrolysis (PEMWE)	Solid polymer membrane	50–80 °C	H^+^ ions
Solid Oxide Electrolysis Cells (SOECs)	Ceramic oxide electrolyte	700–1000 °C	O^−2^ ions
Anion Exchange Membrane Water Electrolysis (AEMWE)	Solid anion exchange membrane	40–70 °C	OH^−^ ions

**Table 2:** Comparative Overview of Hydrogen Storage Technologies.

Storage Technology	Operating Conditions	Operating Conditions
Compressed Hydrogen	350–700 bar, ambient temperature	350–700 bar, ambient temperature
Cryogenic Liquid Hydrogen	−253 °C	−253 °C (20 K), ~1–10 bar
Metal Hydrides	Moderate temperature and pressure	25–350 °C; 1–100 bar
Chemical Hydrides	Moderate conditions	Moderate temperature (80–300 °C)
Metal–Organic Frameworks (MOFs)	Often low temperature	Often 77 K; moderate pressure
Carbon-Based Materials	Variable	Variable temperature and pressure
Liquid Organic Hydrogen Carriers (LOHCs)	Moderate temperatures	Hydrogenation: 120–200 °C; Dehydrogenation: 250–320 °C
Ammonia-Based Storage	Moderate pressure	Moderate pressure (8–10 bar at 25 °C) or refrigerated

**Table 3:** Comparative Overview of Major Fuel Cell Technologies.

Fuel Cell Type	Electrolyte	Operating Temperature
Proton Exchange Membrane Fuel Cell (PEMFC)	Solid polymer membrane	60–80 °C
Solid Oxide Fuel Cell (SOFC)	Ceramic oxide electrolyte	600–1000 °C
Proton Ceramic Fuel Cell (PCFC)	Proton-conducting ceramic	400–700 °C
Alkaline Fuel Cell (AFC)	KOH alkaline electrolyte	60–250 °C
Molten Carbonate Fuel Cell (MCFC)	Molten carbonate salts	~650 °C
Direct Methanol Fuel Cell (DMFC)	Polymer membrane	50–130 °C
Anion Exchange Membrane Fuel Cell (AEMFC)	Anion exchange membrane	40–80 °C

## Data Availability

No new data were created or analyzed in this study. Data sharing is not applicable to this article.

## Abbreviations

Abbreviation	Full Form
AEMWE	Anion Exchange Membrane Water Electrolysis
AWE	Alkaline Water Electrolysis
HER	Hydrogen Evolution Reaction
OER	Oxygen Evolution Reaction
ORR	Oxygen Reduction Reaction
PEMFC	Proton Exchange Membrane Fuel Cell
PEMWE	Proton Exchange Membrane Water Electrolysis
PCFC	Proton Ceramic Fuel Cell
SOFC	Solid Oxide Fuel Cell
SOEC	Solid Oxide Electrolysis Cell
FCEV	Fuel Cell Electric Vehicle
LOHC	Liquid Organic Hydrogen Carrier
